# How active are our children? Findings from the Millennium Cohort Study

**DOI:** 10.1136/bmjopen-2013-002893

**Published:** 2013-07-25

**Authors:** Lucy J Griffiths, Mario Cortina-Borja, Francesco Sera, Theodora Pouliou, Marco Geraci, Carly Rich, Tim J Cole, Catherine Law, Heather Joshi, Andrew R Ness, Susan A Jebb, Carol Dezateux

**Affiliations:** 1 MRC Centre of Epidemiology for Child Health, UCL Institute of Child Health, London, UK; 2 Department of Quantitative Social Science, Institute of Education, University of London, London, UK; 3 School of Oral and Dental Sciences, Bristol Dental School, Bristol, UK; 4 MRC Human Nutrition Research, Elsie Widdowson Laboratory, Cambridge, UK

**Keywords:** Public Health, Sports Medicine, Paediatrics

## Abstract

**Objective:**

To describe levels of physical activity, sedentary time and adherence to Chief Medical Officers (CMO) physical activity guidelines among primary school-aged children across the UK using objective accelerometer-based measurements.

**Design:**

Nationally representative prospective cohort study.

**Setting:**

Children born across the UK, between 2000 and 2002.

**Participants:**

6497 7-year-old to 8-year-old singleton children for whom reliable accelerometer data were available for at least 10 h a day for at least 2 days.

**Main outcome measures:**

Physical activity in counts per minute (cpm); time spent in sedentary and moderate-to-vigorous intensity physical activity (MVPA); proportion of children meeting CMO guidelines (≥60 min/day MVPA); average daily steps.

**Explanatory measures:**

Gender, ethnicity, maternal current/most recent occupation, lone parenthood status, number of children in the household and country/region of residence.

**Results:**

The median daily physical activity level was 595 cpm (IQR 507, 697). Children spent a median of 60 min (IQR 47–76) in MVPA/day and were sedentary for a median of 6.4 h/day (IQR 6–7). Only 51% met CMO guidelines, with girls (38%) less active than boys (63%). Children took an average of 10 229 (95% CI (8777 to 11 775)) steps each day. Children of Indian ethnicity were significantly less active overall than all other ethnic groups. Children of Bangladeshi origin and those living in Northern Ireland were least likely to meet CMO guidelines.

**Conclusions:**

Only half of 7-year-old children in the UK achieve recommended levels of physical activity, with significant gender, ethnic and geographic variations. Longitudinal studies are needed to better understand the relevance of these (in)activity patterns for long-term health and well-being. In the meantime population-wide efforts to boost physical activity among young people are needed which are likely to require a broad range of policy interventions.

**Article summary**

**Article focus**

- This paper describes levels of physical activity (PA), sedentary time and adherence to Chief Medical Officers PA guidelines among primary school-aged children across the UK using objective accelerometer-based measurements.

**Key messages**

- Fifty-one per cent of 7-year-old UK children achieve current recommendations for daily PA; this is significantly lower in girls (38%) than in boys (63%). This is also lowest in children living in Northern Ireland.
- Half of all UK 7-year-olds are sedentary for 6.4 h or more each day.
- Social and demographic variations in physical (in) activity levels are otherwise smaller.
- A comprehensive policy response is needed to boost PA and decrease sedentary time among all young children to the levels appropriate for good health.

**Strengths and limitations of this study**

- This is the first UK-wide study of children's objectively measured physical (in) activity.
- Linkage of the accelerometer data to a range of social and demographic information enabled examination of ethnic, social and geographical variations in activity levels and adherence to policy recommendations.
- While accelerometers provide higher levels of measurement precision relative to self-report methods of PA, they can underestimate certain activities like swimming.
- The prevalence of adherence to current recommendations for PA is sensitive to the thresholds used to define moderate and vigorous PA.

## Introduction

The physical and psychological benefits of physical activity (PA) for children and adolescents include reduced adiposity^1^ and cardiometabolic risk factors,^2^ improvements in musculoskeletal health^3^ and psychological well-being.^4^ Conversely, and independently of PA levels, high levels of sedentary behaviours such as TV viewing may also predict a poor cardiometabolic risk profile.^5^ As PA as well as sedentary behaviour track over time,^6^ ^7^ children with low levels of activity may be at risk of continuing at this level in later life.

Guidelines for PA in children have recently been revised in the USA,^8^ Canada^9^ and the UK.^10^ The latter were launched in July 2011 as a UK-wide consensus from the four nations’ Chief Medical Officers (CMO) on the amount and type of PA for good health at each stage of the lifecourse.^10^ It is now recommended that all young people engage in moderate-to-vigorous intensity physical activity (MVPA; table 1) for at least 60 min and up to several hours every day. A greater emphasis is now placed on vigorous intensity activities and, for the first time, it is recommended that extended sedentary time should be reduced (although a daily limit is not specified).

**Table 1:** Examples of sedentary, moderate and vigorous intensity physical activities

Activity level	Age-adjusted metabolic equivalent (MET)*	Examples^10^
Sedentary	1.0–1.5	Lying, sitting, watching TV, playing video games
Moderate	3.0–5.9	Bike riding and playground activities. These activities will cause children to get warmer and breathe harder and their hearts to beat faster, but they should still be able to carry on a conversation
Vigorous	6.0–8.9	Fast running and swimming. These activities will cause children to get warmer, breathe much harder and their hearts to beat rapidly, making it more difficult to carry on a conversation

*MET, metabolic equivalent; 1 MET is defined as the energy expenditure of sitting quietly, equivalent in children to a resting oxygen intake of ∼6 mL/kg/min.^11^

The development of accelerometer-based activity monitors has enabled the frequency, intensity and duration of free-living activity to be measured objectively in large-scale population studies^12–14^ documenting the proportion of children meeting recommended guidelines.^15^ To date however, accelerometers have not been used in a representative sample of children from all four UK countries.

We report on the time spent in PA and sedentary behaviour in a UK-wide representative sample of primary school-aged children participating in a cohort study. We also evaluate adherence to CMO guidelines and variation according to a range of sociodemographic characteristics.

## Methods

The Millennium Cohort Study (MCS) is a prospective study of the social, economic and health-related circumstances of children born in the UK between September 2000 and January 2002.^16^ The original cohort comprised 18 818 children (72% of those approached) whose parents were first interviewed at home when their child was aged 9 months. Since then data have been collected when the children were aged 3, 5 and 7 years, with follow-up currently being conducted at 11 years and planned for 14 years and beyond.

### PA data

PA and sedentary time were measured using the Actigraph GT1M accelerometer (Actigraph, Pensacola, Florida), which has been demonstrated to measure PA reliably in children when compared with measures derived from heart rate monitoring,^17^ indirect and room calorimetry^18^ ^19^ and doubly labelled water.^20^

A total of 14 043 children (13 681 singletons) were interviewed at age 7 and invited to participate in the accelerometry study. Those who consented were subsequently sent an accelerometer in the post, programmed to use a 15 s sampling epoch and to record activity as counts and steps. Participants were instructed to start wearing their accelerometer, on an elastic belt round the waist, the morning after they received it and to continue doing so during waking hours for seven consecutive days. They were asked to remove the accelerometer when bathing or during other aquatic activities.

**Figure 1:**
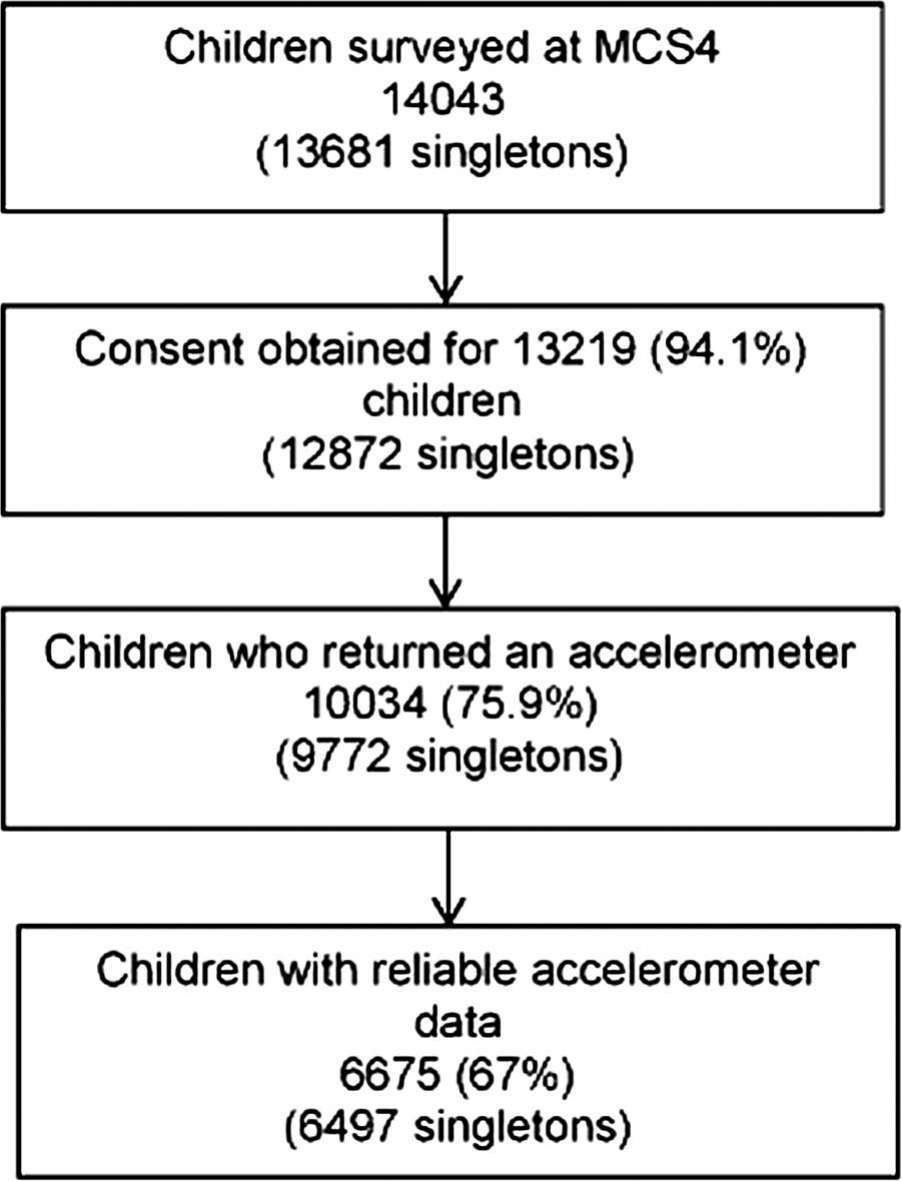
Flow diagram of study participants.

Data were collected between May 2008 and August 2009. Accelerometers were returned by 9772 singleton children. Data were downloaded using Actigraph software V.3.8.3 (Actigraph, Pensacola, Florida, USA) and subsequently processed in-house,^21^ according to predetermined processing criteria.^22^ Non-wear time, defined as time periods of consecutive zero activity counts of 20 min or more, was excluded from analysis. Extreme count values above a threshold of ≥11 715 counts/min (cpm) were removed. A study was performed to discriminate between days when the accelerometer was worn and days when it was likely not worn (eg, during postal delivery) on the basis of the minimum number of valid minutes/day. The threshold was set to ≥150 min/day. The first day with registered time exceeding the threshold was set as start date. The last day with registered time above the threshold was set as end date.^22^ Participants with registered time of ≥10 h on at least ≥2 days were included in analyses; only days with ≥10 h of recorded time were included as this has been shown to produce reliable estimates of PA.^23^ This resulted in a final sample size of 6497 singleton children (3176 boys; figure 1) with a total of 36 309 days (nearly 449 000 h) of observation.

Small differences were found in the demographic characteristics of the sample of children with reliable accelerometer data relative to the whole cohort sample (n=13 681) interviewed at age 7 years (see online supplementary eTable 1). In particular, boys, children living in Wales, those belonging to the Indian, Pakistan/Bangladeshi and Black Caribbean/African ethnic groups, as well as children belonging to families with a single respondent at the interview showed a lower probability of having a valid accelerometer file. Information on predictors of reliable data acquisition in this study is available elsewhere.^24^

For each child the following variables were derived: total PA (average cpm) over the period worn, mean daily steps and minutes of sedentary time (defined as <100 cpm) and MVPA (>2241 cpm). These cut-off values were established to identify sedentary behaviour and light, moderate and vigorous PA using linear discriminant analysis and evaluated using receiver-operating characteristic curve analysis, and were taken from a calibration study in 7-year-olds, carried out specifically for this larger study.^25^ They are similar to those reported by Evenson *et al*.^26^ The start and end dates were detected based on a specified amount of daily waking time (defined as between 07:00 and 21:59) required to be different from zero counts: the threshold for the observational period was again set to at least 150 min/day. Total registered time is not constant across days. As a result, the total amount of counts and wearing time for each child will depend on how long the accelerometer was worn. These measures were standardised by introducing the notion of a standard day with equal duration (ie, 735 min, equal to mean wear time across all reliable days).^27^ For each child time spent in MVPA was used to determine adherence to the CMO PA guideline (≥60 min daily) and was based on the standardised average minutes/day spent in MVPA, where the objective of standardisation was to standardise wearing time across days.

### Explanatory variables

We examined the association of the following social and demographic factors, collected in the home-based interview at 7 years: child's gender; age; ethnicity;^28^ maternal current or most recent occupation by child age 7^29^; family structure (lone parent and number of children) and country of residence (England, Scotland, Wales and Northern Ireland) and (within England) Government Office Region of residence. Yearly seasons of measurement were defined astronomically, that is, spring (21 March–20 June), summer (21 June–20 September), autumn (21 September–20 December) and winter (21 December–20 March).

### Statistical analyses

Analyses were conducted using STATA/SE V.11.2 (Stata Corporation, Texas, USA) and the R software environment for statistical computing V.2.15.0.^30^ ^31^ Sampling weights were used to account for the stratified clustered design of the MCS. Weights were adjusted for attrition between contacts at successive MCS sweeps and for missing accelerometer data. Details on the adjustment for non-response and non-compliance are given elsewhere.^27^ Prevalences of the children meeting the CMO current PA guideline were derived, as were medians and IQRs for derived activity variables (given their non-normal distributions). Quantile regression models for medians and quartiles were fitted using the R package quantreg^32^ to examine differences between sociodemographic groups in terms of time spent in physical (in)activity adjusting for season of measurement. These models do not make assumptions about the shape of the distribution. Therefore they are robust to departures from normality, as for example, skewness, heavy-tailedness and heteroscedasticity, which affected all PA outcomes in this study. The Benjamini-Hochberg^33^ correction for false discovery rate in multiple comparisons was also applied setting the point-wise threshold for the correction at 0.05. We used the R package multtest^34^ to adjust p values for multiple comparisons using this correction. Multivariable logistic regression models were fitted to analyse differences between sociodemographic groups for prevalences of children meeting the MVPA guideline. Prevalences were adjusted for gender, ethnicity, maternal current or most recent occupation, lone parenthood status, number of children in the household, country of residence and season of measurement. Moran's I test^35^ was used to assess spatial autocorrelation among regions within England using a weighted matrix with a second-order nearest neighbourhood structure.^36^

## Results

The majority of children in the sample (85%) were white (see online supplementary eTable 1), 49% were boys, 88% were living in households including at least one other child and 65% lived in England. The current or most recent occupation of mothers by child age 7 years was semiroutine and routine in 36%; 22% of mothers were lone parents. Mean age when wearing the accelerometer was 7.5 years.

Seasonally adjusted medians of derived PA variables are summarised in table 2.

**Table 2:** Description of physical activity (PA) measures; adjusted^(^*^)^ median and IQR, unless otherwise stated (N=6497)

	N	Overall activity (counts/min)	Sedentary (hours/day)	Moderate and vigorous (min/day)	Steps/day	Percentage of children meeting recommended PA (SE^(†)^)
All children	6497	595 507, 697	6.4 5.9, 6.9	60.1 47.1, 76.2	10 229 8777, 11 775	50.8 (0.009)
Child's gender						
Male	3176	630^a (§)^ 541, 757	6.4^a^ 5.9, 6.9	67.1^a^ 53.9, 83.7	10739^a^ 9213, 12 499	63.3^a^ (0.012)
Female	3321	561^a^ 481, 656	6.5^a^ 6.0, 7.2	54.4^a^ 44.4, 67.1	9699^a^ 8389, 11 063	37.8^a^ (0.011)
Child's ethnicity^(‡)^						
White	5710	597^a^ 512, 701	6.5^a,b^ 6.0, 7.1	60.2^a^ 47.8, 75.9	10343^a–f^ 8926, 11 940	51.4 (0.010)
Mixed	168	572^b^ 505, 643	6.8^a^ 6.3, 7.3	62.2^b^ 49.9, 66.7	10071^a,g–i^ 8553, 11 167	51.5 (0.040)
Indian	139	511^a–e^ 437, 680	6.9^b–f^ 6.4, 7.4	52.6^a–d^ 40.9, 66.7	8699^b,g^ 7477, 9667	40.0 (0.049)
Pakistani	177	563^c^ 484, 680	6.4^c^ 5.9, 7.0	58.2 46.4, 74.8	9419^c,h^ 7906, 11 120	45.2 (0.058)
Bangladeshi	70	538 450, 639	6.5^d^ 6.0, 7.0	52.9 42.1, 70.6	8894^d,i^ 6889, 10 013	32.8 (0.087)
Black	142	598^d^ 517, 737	6.5^e^ 6.0, 7.0	63.1^c^ 52.6, 86.3	9468^d^ 7878, 10 857	52.0 (0.054)
Other	90	581^e^ 476, 650	6.5^f^ 6.1, 7.7	58.2 45.8, 74.1	9814^f^ 8270, 10 798 11 710 440	53.9 (0.081)
Maternal current or most recent occupation by child age 7						
Managerial and professional occupations	2190	585 502, 690	6.6^a^ 6.0, 7.1	59.4 46.8, 74.6	10 135 8685, 11 662	48.8^a^ (0.013)
Intermediate occupations	1202	597 511, 697	6.5^b^ 6.1, 7.0	58.39.2 47.8, 75.8	10 239 8843, 11 722	48.7^b^ (0.017)
Small employers and own account workers	442	596 516, 671	6.6 6.1, 7.0	60.5^c^ 48.3, 73.6	10 237 8901, 11 598	48.6^c^ (0.031)
Lower supervisory and technical occupations	288	598 505, 675	6.6^c^ 6.1, 7.0	60.5 48.3, 73.6	10 152 8596, 11 710	51.0 (0.033)
Semiroutine and routine occupations	1865	597 508, 710	6.5^d^ 5.9, 7.0	61.5 47.3, 77.2	10 380 8891, 12 127	52.3 (0.015)
Never worked and long-term unemployed	268	603 529,, 607	6.3^a–d^ 5.9, 6.9	62.2 49.1, 77.0	10 116 8702, 11 017	59.7^a–c^ (0.040)
Lone parenthood status						
No	5536	589^a^ 504, 693	6.54^a^ 6.0, 7.1	59.6^a^ 47.1, 75.0	10127^a^ 8683, 11 703	49.7^a^ (0.010)
Yes	961	606^a^ 521, 712	6.48^a^ 5.9, 7.0	63.0^a^ 50.3, 78.2	10471^a^ 9026, 12 242	54.7^a^ (0.021)
No. of children in the household						
Only child	726	594 510, 679	6.6^a^ 6.1, 7.1	61.9 48.0, 74.9	10 259 8920, 11 950	51.9 (0.023)
At least one other child	5771	593 507, 697	6.5^a^ 6.0, 7.0	60.0 47.5, 75.9	10 222 8761, 11 762	50.7 (0.009)
Country						
England	4204	589^a,b^ 504, 692	6.6^a,b^ 6.0, 7.1	60.6^a^ 46.9, 75.3	10147^a,b^ 8743, 11 763	50.9^a^ (0.011)
Wales	898	606^a^ 509, 711	6.5^a^ 5.9, 7.0	61.6^b^ 47.0, 77.8	10357^a,c^ 8931, 11 922	51.7^b^ (0.021)
Scotland	761	615^b,c^ 578, 715	6.4^b,c^ 5.9, 6.9	61.8^c^ 50.5, 78.1	10521^b,d^ 9022, 12 027	52.5^c^ (0.024)
Northern Ireland	634	586^c^ 486, 617	6.6^c^ 6.0, 7.1	57.6^a–c^ 45.5, 73.4	9895^c,d^ 8031, 11 351	43.4^a–c^ (0.021)

(*) Medians and IQR adjusted by seasons of measurement.

(†) The prevalences of children meeting the recommended MVPA guidelines are adjusted for gender, ethnicity, maternal current or most recent occupation, lone parenthood status, number of children in the household, country of residence and season of measurement.

(‡) This variable was based on a derived variable from the 2001 Census ethnicity categories indicating the ethnic identity of the children in the MCS data. Aggregate groupings were imposed on these categories to create a smaller manageable number of categories. The names of the groups were also adjusted for reporting. The final ethnic groups were: White (including White-British, White-Irish, White-Welsh, White-Scottish and other white background); Mixed; Indian; Pakistani; Bangladeshi; Black (including Black-Caribbean, Black-African and Black-British) and other (including other Asian, other Black, Chinese and other).

(§) As there are many multiple comparisons among levels of factors included in the models, this table does not show any p values and only indicates those pairwise differences which were significantly different after applying the Benjamini-Hochberg correction for false discovery rate. We used superindices (a–i) to indicate pairs of groups within a factor that differed significantly pairwise (p<0.05). For example, medians of minutes of overall activity significantly differed pairwise between England and Wales (a), England and Scotland (b), and Scotland and Northern Ireland (c).

Median PA was 595 (IQR 507, 696) cpm. Median daily minutes spent in MVPA was 60. (IQR 47.1, 76.2) and 50% of children were sedentary for 6.4 h (IQR 5.9, 6.9) or more each day. Children took a median of 10 229 (IQR 8777, 11 775) daily steps. We estimated that half of all children (50.8%) achieved ≥60 min of daily MVPA.

Girls engaged less in total PA and MVPA, and took fewer steps than boys. They were also more sedentary and were less likely than boys to meet the daily MVPA recommendation (37.8% vs 63.3%).

**Figure 2:**
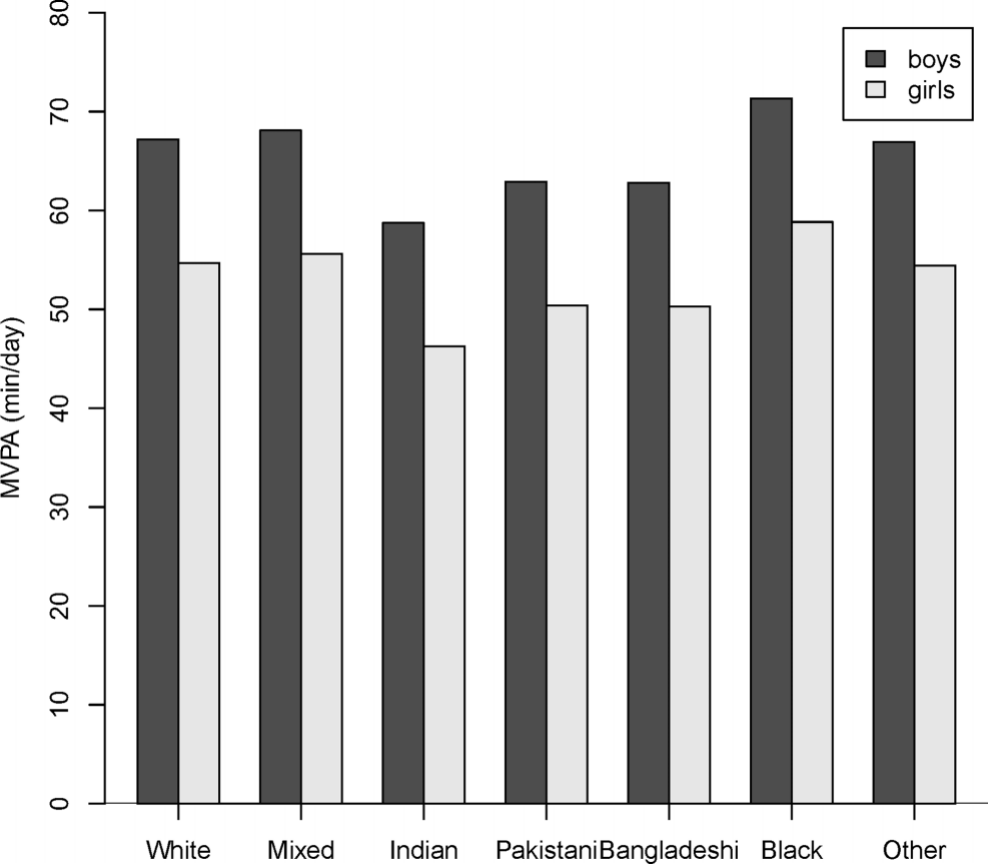
Median minutes/day of moderate-to-vigorous intensity physical activity by gender and ethnicity adjusting by season of measurement.

Overall activity levels were lowest in children of Indian origin, who also recorded the least MVPA, were among the most sedentary and took the fewest steps. MVPA levels were lower in girls than in boys across all ethnic groups (figure 2). Bangladeshi children were the least likely (32.8%) to meet the MVPA recommendation. In pairwise comparisons children of Indian ethnicity were significantly less active overall than all other ethnic groups. Those children whose mothers have never worked or had been long-term unemployed were the most likely to meet recommended levels of daily PA and had the lowest median of daily hours of sedentary behaviour.

Children from couple parent families engaged in less total PA and daily MVPA than those whose mothers did not live with partners. They also took the least steps and were least likely to meet the MVPA recommendation. Little variation was found in PA levels according to the number of children living in the household, although lone children in the household were slightly more sedentary.

**Figure 3:**
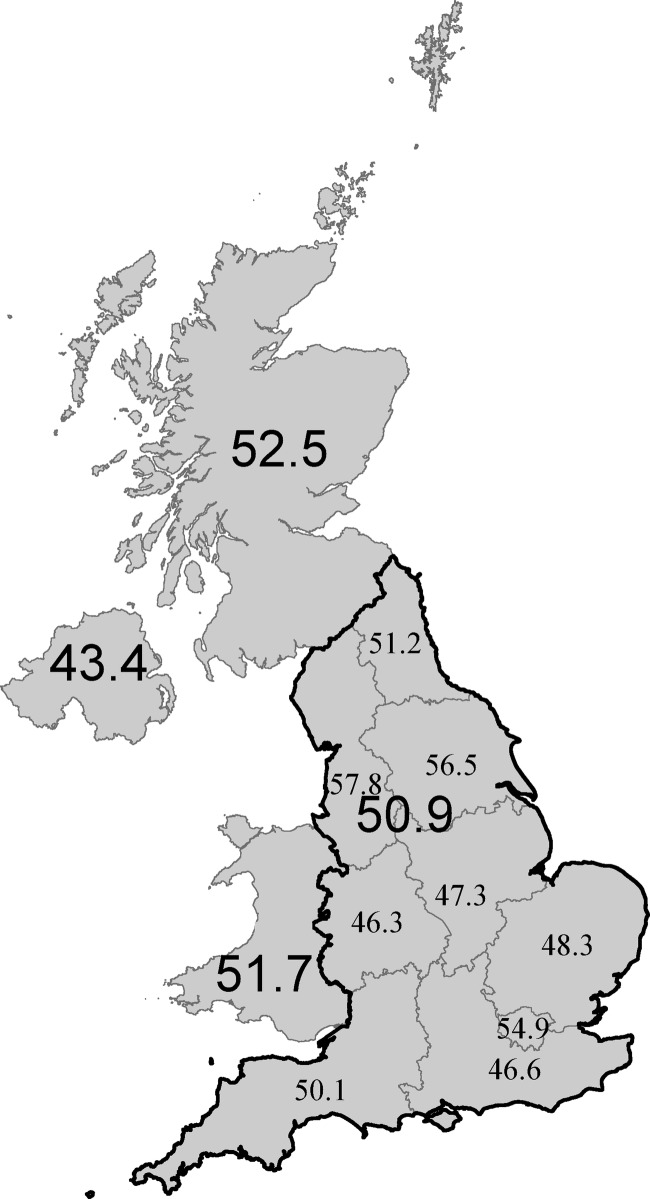
Prevalence of children meeting physical activity recommendation of ≥60 min/day moderate-to-vigorous intensity physical activity (%).

There were significant differences between UK countries: those living in Northern Ireland engaged in the least total PA, daily MVPA and took the fewest steps. Children in Scotland were the least sedentary, Children in Northern Ireland had the lowest prevalence of the recommended amount of daily MVPA (43.4%), while 50.9% of those living in England, 51.7% of those in Wales and 52.5% of those in Scotland, achieved the recommended amount of daily MVPA. Though spatial autocorrelation was not statistically significant (I=0.05, p=0.822) some regional differences within England were also identified (figure 3); children in the North West were the most likely (57.8%) and those in the Midlands the least likely (46%) to meet the CMO guideline.

## Discussion

### Statement of principal findings

This is the first population-based UK-wide study of objectively measured PA and sedentary time in primary-school aged children. We found that when measured in 2008–2009, 51% of children met the CMO recommendation of 60 min or more of daily MVPA.^10^ ^37^ More than half of all children spent a substantial part of the day being sedentary.

This study highlights social and demographic variation in the UK children's PA and inactivity. Girls were more sedentary than boys and less active across all intensities of PA. Ethnic differences were observed with children of Indian origin generally the least active across all outcomes and Bangladeshi children were least likely to achieve recommended levels of PA. There were no clear socioeconomic gradients in PA levels and adherence to guidelines, although there was a tendency for children whose mothers were never employed or unemployed to be slightly more active than children whose mothers were employed. Children of lone parents were slightly more active and more likely to meet the MVPA recommendations than those from couple parent families. There were differences between UK countries with children in Northern Ireland less active and least likely to achieve recommended activity levels than those from other countries. Nevertheless, with the exception of gender, the group differences observed in this study were relatively small and the most striking finding is the low level of activity across all groups.

### Strengths and limitations

The strengths of this study include the large UK-wide and representative sample, the use of accelerometers to provide objective measures of PA and sedentary time, and the data processing undertaken to exclude any data that might be unreliable. As there is limited consensus on data reduction strategies,^38^ most commonly relating to the best threshold with which to define MVPA, we derived the thresholds used in our study from a calibration study we conducted specifically in this age group and consider the thresholds used to be reliable.^25^ The ability to link accelerometer data to a rich range of social and demographic information has enabled examination, for the first time, of ethnic, social and geographical variations in adherence to policy recommendations. Acknowledged limitations include potential underestimation, of PA as a result of the accelerometers used, the position in which they are worn or their removal during some activities, such as aquatic activities or contact sports.

### Comparison with literature

Findings from the large (defined as ≥400 participants) UK-based accelerometry studies reporting adherence to PA guidelines are summarised in table 3.

**Table 3:** Summary of accelerometry studies in the UK-based school-aged children

					Prevalence sufficiently active* (%)	
Reference and study name/size	Setting	Year measured	Mean age (years)	Threshold used to define MVPA	Boys	Girls
National probability samples						
MCS (current study) (n=6497)	England, Scotland, Wales and Northern Ireland	2008–2009	7.5	2240 cpm^25^	63	38
HSE 2008^39^ (n=1286)	England	2008	9.9	2802 cpm^39^	33	21
Regional samples						
Basterfield *et al*^40^ Gateshead Millennium Study (n=405)	England (Gateshead)	2006–2007 and 2008–2009	7.4 and 9.3	3200 cpm^19^	7 years: 10.1 9 years: 8.6	7 years: 2.9 9 years: 2.9
Owen *et al*^41^ CHASE (n=2071)	England (London, Birmingham and Leicester)	2006–2007	9.9	2000 cpm^18^	76	53
Steele *et al*^42^ SPEEDY (n=1862)	England (Norfolk)	2007	10.2	2000 cpm^18^	81.5	59.4
Gidlow *et al*^43^ CHAMPS UK (n=503)	England (Stoke-on-Trent)	2006–2007	10.4	Age-specific thresholds^44^ and 3200 cpm^19^	94^44^ 5.2^19^	89^44^ 1.4^19^
Riddoch *et al*^45^ ALSPAC (n=5595)	England (Bristol)	2003–2005	11	3600 cpm^46^	5.1	0.4

*Accumulating ≥60 min/day MVPA. The exact measure used to define adherence to this guideline varies across the studies tabulated: in MCS, Gateshead, SPEEDY and ALSPAC studies a mean of 60 min or more was used; in the CHASE study only the first day of measurement, and in the Health Survey only a subsample with 7 days of observation each with at least 60 min of MVPA/day.

ALSPAC, Avon Longitudinal Study of Parents and Children; CHAMPS, Children's Health and Activity Monitoring Programme in Schools; CHASE, Child Heart and Health Study; cpm, counts per minute; MCS, Millennium Cohort Study; MPVA, moderate-to-vigorous intensity physical activity; SPEEDY, Sport, Physical Activity and Eating Behaviour, Environmental Determinants in Young People.

In none were children measured in population-based samples from all four UK countries. The Millennium Gateshead Study measured children of a similar age to those in our study, although in a much smaller sample. The results show wide variation in the proportions reported to adhere to recommended guidelines on activity but are consistent with our finding that boys are more active than girls. These differences may in part be due to the use of different MVPA intensity thresholds, ranging from 2000 to 3600 cpm. We examined this by recalculating our data using different thresholds: the percentage of children meeting recommendations increased to 80.4% (SE of proportion: 0.010) and 59.4% (0.011) for boys and girls, respectively, when applying a threshold of 2000 cpm and fell to 13.7% (0.008) and 0.4% (0.005) when applying a higher threshold of 3000 cpm. Clearly, therefore, our estimates are similar to some of these other studies^41^ ^42^ when a similar threshold of 2000 cpm is applied. However, other differences may reflect variation in regions, sampling methods and settings for recruitment as well as in approaches to adjustment for missing data. Further work is needed to clearly define the best thresholds for different intensities of activity in children incorporating novel statistical techniques.^47^ Clarity over thresholds will be important for routine surveillance measures to monitor the achievement of policy objectives relating to PA. Differences in estimates of sedentary time and numbers of steps between studies may reflect measurement period, while decisions made about removal of zero values may also influence sedentary time. These parameters are not consistently reported in published reports.

Only one other large-scale study in the UK has examined ethnic differences in objectively measured PA. Owen *et al*^41^ reported lower levels in South Asian children than European whites and black African-Caribbeans, with 54%, 70% and 69%, respectively, meeting the recommendations. We also found that children of Bangladeshi origin were the least likely to meet these recommendations. Further research is however needed to explore the different cultural and social factors that determine these ethnic differences in activity levels.

Although activity levels differed between children from different socioeconomic circumstances, with those less advantaged slightly more active, we did not find a strong social gradient; this is consistent with other published evidence.^45^ ^48^ This is the only study to report country and regional differences across the UK and so direct comparisons with other studies cannot be made.

The children in our study engaged in slightly less sedentary time than observed in older children in other UK-based accelerometry studies. For example, in the Avon Longitudinal Study of Parents and Children 7 and 7.3 h of daily sedentary time were reported in 12-year-old boys and girls, respectively, increasing at older ages.^49^ In a cross-sectional study of English 10-year-olds, Steele *et al*^42^ reported 7.5 and 7.7 h for boys and girls, respectively.

Tudor-Locke *et al*^50^ recommend that school-aged boys and girls should be encouraged to undertake 13 000–15 000 and 11 000–12 000 steps/day, respectively. The children in this study accumulated fewer steps than this (boys10 732; girls 9675); however, our estimates are very similar to those reported by Owen *et al*^41^ (boys 10 570; girls 9123) who also reported higher step numbers in white compared to black and Asian children, which is consistent with our findings.

### Unanswered questions and future research

We are currently examining influences on PA and sedentary time in this representative cohort of school-aged children in more detail using longitudinal data to examine their prior determinants. Once follow-up data become available from subsequent contacts with cohort members we propose to examine the consequences for health in later childhood and adolescence. Objective measures of PA and sedentary time can allow these influences to be characterised with greater precision and to be compared with subjective and proxy reports of engagement in PA and sedentary behaviours, also collected within this cohort. We have documented the accelerometer data and derived variables, and these are accessible to other researchers through the UK Data Service.

### Implications for practice and policy

Last year the London 2012 Olympic Games promised to inspire a generation to take part in sport. The UK sporting success in August 2012 provided a platform for the Government's plans for an Olympic and Paralympic sporting legacy,^51^ to encourage and enable pupils to engage in competitive sports and activities. The results of our study provide a useful baseline and strongly suggest that contemporary UK children are insufficiently active, implying that effort is needed to boost PA among young people to the level appropriate for good health. This is likely to require population-wide interventions across the range of PA domains. There are many opportunities to increase PA through sport but also in other areas.^52^ The journey to school has been recognised as an important opportunity for increasing total as well as more intense PA. Urban environments are also important for children’s PA,^53^ and factors from available green space to perceptions of safety all impact on children's activity. The full potential to boost PA will only be realised with a comprehensive policy response that increases time spent in more intense PA and decreases the time spent being sedentary. Investing in this area is a vital component to deliver the Olympic legacy and improve the short and long-term health of our children.

## Supplementary Material

Author's manuscript

Reviewer comments
