# Supplementary material for: How active are our children? Findings from the Millennium Cohort Study
Source: BMJ Open. 2013 Jul 25;3(8):e002893. doi: 10.1136/bmjopen-2013-002893 (PMC3752053; doi:10.1136/bmjopen-2013-002893)
Supplement: Author's manuscript [file bmjopen-2013-002893.draft_revisions.pdf]

How active are our children? Findings from the Millennium Cohort Study

Journal:	*BMJ Open*
Manuscript ID:	bmjopen-2013-002893
Article Type:	Research
Date Submitted by the Author:	15-Mar-2013

Complete List of Authors:	Griffiths, Lucy; UCL Institute of Child Health, MRC Centre of Epidemiology for Child Health<br>Cortina Borja, Mario; UCL Institute of Child Health,<br>Sera, Francesco; UCL Institute of Child Health,<br>Pouliou, Theodora; UCL Institute of Child Health,<br>Geraci, Marco; UCL Institute of Child Health,<br>Rich, Carly; UCL Institute of Child Health,<br>Cole, Tim; UCL Institute of Child Health,<br>Law, Catherine; UCL Institute of Child Health,<br>Joshi, Heather; Institute of Education,<br>Ness, Andy; School of Oral Dental Science,<br>Jebb, Susan; MRC Human Nutrition Research<br>Dezateux, Carol; Institute of Child Health, MRC Centre of Epidemiology for Child Health

**Primary Subject Heading**:	Epidemiology
Secondary Subject Heading:	Public health, Sports and exercise medicine
Keywords:	PUBLIC HEALTH, SPORTS MEDICINE, PAEDIATRICS

SCHOLARONE™
Manuscripts

How active are our children? Findings from the Millennium Cohort Study

Lucy J Griffiths (senior research fellow)[1], Mario Cortina-Borja (senior lecturer)[1], Francesco Sera (senior research associate)[1], Theodora Pouliou (research associate)[1], Marco Geraci (lecturer)[1], Carly Rich (research associate)[1], Tim J Cole (professor)[1], Catherine Law (professor)[1], Heather Joshi (professor)[2], Andrew R Ness (professor)[3], Susan A Jebb (head of diet and population health)[4] and Carol Dezateux (professor)[1]

[1] MRC Centre of Epidemiology for Child Health, UCL Institute of Child Health, 30 Guilford Street, London, WC1N 1EH.

[2] Quantitative Social Science, Institute of Education, University of London, 20 Bedford Way, London, WC1H 0AL.

[3] School of Oral and Dental Sciences, Bristol Dental School, Lower Maudlin Street, Bristol BS1 2LY.

[4] MRC Human Nutrition Research, Elsie Widdowson Laboratory, 120 Fulbourn Road, Cambridge, CB1 9NL.

Corresponding author: Lucy Griffiths; address as above; lucy.griffiths@ucl.ac.uk

Study design: Prospective cohort study

Keywords: physical activity, sedentary, accelerometry, children, cohort study

For peer review only - http://bmjopen.bmj.com/site/about/guidelines.xhtml

ABSTRACT

Objective To describe levels of physical activity, sedentary time and adherence to Chief Medical Officers (CMO) physical activity guidelines among primary school-aged children across the UK using objective accelerometer-based measurements.

Design Nationally representative prospective cohort study.

Setting Children born across the UK, between 2000 and 2002.

Participants 6497 7-8 year old singleton children for whom reliable accelerometer data were available for at least 10 hours a day for at least two days

Main outcome measures Physical activity in counts per minute (cpm); time spent in sedentary and moderate to vigorous intensity physical activity (MVPA); proportion of children meeting CMO guidelines (≥60 min/day MVPA); average daily steps.

Explanatory measures: Gender, ethnicity, maternal socioeconomic circumstances, lone parenthood status, number of children in the household, and country / region of residence.

Results The median daily physical activity level was 594 cpm (interquartile range (IQR): 504, 697). Children spent a median of 61 minutes (IQR: 47-76) in MVPA per day and were sedentary for a median of six and a half hours per day (IQR: 6-7). Only 51% met CMO guidelines, with girls (38%) less active than boys (63%). Children took an average of 10180 (95% CI (8712, 11897)) steps each day. Children of Indian origin and those living in Northern Ireland were least likely to meet CMO guidelines.

Conclusions Only half of seven year old children in the UK achieve recommended levels of physical activity, with significant gender, social, demographic and geographic variations. Population-wide efforts are needed to boost physical activity among young people to the level appropriate for good health. This is likely to require a broad range of policy interventions.

For peer review only - http://bmjopen.bmj.com/site/about/guidelines.xhtml

ARTICLE SUMMARY

Article focus

- This paper describes levels of physical activity, sedentary time and adherence to Chief Medical Officers (CMO) physical activity guidelines among primary school-aged children across the UK using objective accelerometer-based measurements.

Key messages

- 51% of seven year old UK children achieve current recommendations for daily physical activity; this is significantly lower in girls (38%) than in boys (63%). This is also lowest in children living in Northern Ireland.
- Half of all UK seven year olds are sedentary for six and a half hours or more each day.
- Social and demographic variations in physical (in) activity levels are otherwise smaller.
- A comprehensive policy response is needed to boost physical activity and decrease sedentary time among all young children to the levels appropriate for good health.

Strengths and limitations of this study

- This is the first UK-wide study of children's objectively measured physical (in) activity.
- Linkage of the accelerometer data to a range of social and demographic information enabled examination of ethnic, social and geographical variations in activity levels and adherence to policy recommendations.
- While accelerometers provide higher levels of measurement precision relative to self-report methods of physical activity, they can underestimate activities that do not include vertical movement of the trunk (e.g. cycling) and aquatic activities (e.g. swimming).

For peer review only - http://bmjopen.bmj.com/site/about/guidelines.xhtml

INTRODUCTION

The physical and psychological benefits of physical activity for children and adolescents include reduced adiposity[1] and cardiometabolic risk factors,[2] improvements in musculoskeletal health[3] and psychological well-being.[4] Conversely, and independently of physical activity levels, high levels of sedentary behaviours such as TV viewing may also predict a poor cardiometabolic risk profile.[5] As both physical activity and sedentary behaviour track over time,[6;7] children with low levels of activity may be at risk of continuing at this level in later life.

Guidelines for physical activity in children have recently been revised in the United States,[8] Canada[9] and the United Kingdom (UK).[10] The latter were launched in July 2011 as a UK-wide consensus from the four nations' Chief Medical Officers (CMO) on the amount and type of physical activity for good health at each stage of the lifecourse.[10] It is now recommended that all young people engage in moderate to vigorous intensity physical activity (MVPA) (Box) for at least 60 minutes and up to several hours every day. A greater emphasis is now placed on vigorous intensity activities and, for the first time, it is recommended that extended sedentary time should be reduced (although a daily limit is not specified).

Box Examples of sedentary, moderate and vigorous intensity physical activities

Activity Level	Definition	Examples[10]
Sedentary	1.0 MET*s	Lying, sitting, watching TV, playing video games
Moderate	3.0 – 5.9 METs	Bike riding and playground activities. These activities will cause children to get warmer and breathe harder and their hearts to beat faster, but they should still be able to carry

For peer review only - http://bmjopen.bmj.com/site/about/guidelines.xhtml

		on a conversation
Vigorous	6.0 - 8.9 METs	Fast running and swimming. These activities will cause children to get warmer, breathe much harder and their hearts to beat rapidly, making it more difficult to carry on a conversation.

*MET: metabolic equivalent; 1 MET of energy is used by the body at rest.

The development of accelerometer-based activity monitors has enabled the frequency, intensity and duration of free-living activity to be measured objectively in large scale population studies,[11-13] documenting the proportion of children meeting recommended guidelines.[14] To date however, accelerometers have not been used in a representative sample of children from all four UK countries.

We report on the time spent in physical activity and sedentary behaviour in a UK-wide representative sample of primary school-aged children participating in a cohort study. We also evaluate adherence to CMO guidelines and variation according to a range of socio-demographic characteristics.

METHODS

The Millennium Cohort Study (MCS) is a prospective study of the social, economic and health-related circumstances of children born in the UK between September 2000 and January 2002.[15] The original cohort comprised 18,818 children (72% of those approached) whose parents were first interviewed at home when their child was aged nine months. Since then data have been collected when the children were aged three, five and seven years, with follow-up currently being conducted at 11 years and planned for 14 years and beyond. This study received research ethics

For peer review only

For peer review only - http://bmjopen.bmj.com/site/about/guidelines.xhtml

approval from the Northern and Yorkshire Research Ethics Committee (07/MRE03/32).

Physical activity data

Physical activity and sedentary time were measured using the Actigraph GT1M accelerometer (Actigraph, Pensacola, Florida), which has been demonstrated to measure physical activity reliably in children when compared to measures derived from heart rate monitoring,[16] indirect and room calorimetry [17;18] and doubly labelled water.[19]

A total of 14,043 children (13,681 singletons) were interviewed at age seven years and invited to participate in the accelerometry study. Those who consented were subsequently sent an accelerometer in the post, programmed to use a 15 second sampling epoch and to record activity as counts and steps. Participants were instructed to start wearing their accelerometer, on an elastic belt round the waist, the morning after they received it and to continue doing so during waking hours for seven consecutive days. They were asked to remove the accelerometer when bathing or during other aquatic activities.

Data were collected between May 2008 and August 2009. Accelerometers were returned by 9772 singleton children. Data were downloaded using Actigraph software version 3.8.3 (Actigraph, Pensacola, Florida) and subsequently processed in-house,[20] according to pre-determined processing criteria.[21] Non-wear time, defined as time periods of consecutive zero activity counts of 20 minutes or more, was excluded from analysis. Extreme count values above a threshold of ≥11,715 cpm were removed. Participants with registered time of ≥10 hours on at least ≥2 days were included in analyses; only days with ≥10 hours of recorded time were included. This resulted in a final sample size of 6497 singleton children (3176 boys) (Figure 1) with a total of 36,309 days (nearly 449000 hours) of observation.

For peer review only - http://bmjopen.bmj.com/site/about/guidelines.xhtml

Small differences were found in the demographic characteristics of the sample of children with reliable accelerometer data relative to the whole cohort sample (*n*=13681) interviewed at age seven years (eTable1). Information on predictors of reliable data acquisition in this study is available elsewhere.[22]

For each child the following variables were derived: total physical activity (average counts per minute (cpm)) over the period worn, mean daily steps and minutes of sedentary time (defined as <100 cpm) and MVPA (>2241 cpm). These cut-offs were chosen to optimise the discrimination between activity levels and were taken from a calibration study in seven year olds, carried out specifically for this larger study.[23] For the mean daily steps and minutes of sedentary time and MVPA, which were related to the wear time period of the accelerometer, the summary measures for each child were calculated as a weighted mean of the daily values, with weight calculated as the powered inverse of the ratio between observed wear time and standard wear time. Standard wear time was fixed at 735 minutes, which was the mean wear time across all reliable days. The power values were chosen to minimize the correlation between summary variables and daily wear time. Time spent in MVPA was used to determine adherence to the CMO physical activity guideline (≥60 minutes daily).

Explanatory variables

We examined the association of the following social and demographic factors, collected in the home-based interview at seven years: child's gender; age; ethnicity;[24] maternal socioeconomic status;[25] family structure (lone parent and number of children); and country of residence (England, Scotland, Wales, Northern Ireland) and (within England) Government Office Region of residence.

Statistical analysis

For peer review only - http://bmjopen.bmj.com/site/about/guidelines.xhtml

Analyses were conducted using STATA/SE 11.2 (Stata Corporation, TX) and the R software environment for statistical computing version 2.15.0.[26;27] Sampling weights were used to account for the stratified clustered design of the MCS. Weights were adjusted for attrition between contacts at successive MCS sweeps and for missing accelerometer data. Percentages were derived, as were medians and interquartile ranges for derived activity variables (given their non-normal distributions). Median regression models were fitted using the R package `quantreg`[28] to examine differences between socio-demographic groups in terms of time spent in physical (in)activity. These nonparametric models provide a flexible framework to analyse skewed distributions. The Benjamini-Hochberg[29] correction for false discovery rate in multiple comparisons was also applied setting the point-wise threshold for the correction at 0.05. We used the R package `multtest`[30] to compute this correction. Adjusted logistic regression models were fitted to analyse differences between socio-demographic groups for percentages meeting the MVPA guideline. Moran's *I* test[31] was used to assess spatial autocorrelation among regions within England using a weighted matrix with a second-order nearest neighbourhood structure.[32]

RESULTS

The majority of children in the sample (85%) were white (eTable1), 49% were boys, 88% were living in households including at least one other child and 65% lived in England. The mothers of 35% of children were in semi-routine and routine occupations, and 22% were lone parents. Mean age when wearing the accelerometer was 7.5 years.

Absolute levels of derived physical activity variables and their differences are summarised according to the socio-demographic characteristics in Table 1.

Median physical activity was 594 (interquartile range (IQR): 504, 697) cpm. Median daily minutes spent in MVPA was 61 (IQR: 47-76) and fifty per cent of

For peer review only - http://bmjopen.bmj.com/site/about/guidelines.xhtml

children were sedentary for six and a half hours (6, 7) or more each day. Children took an average of 10180 (8712, 11897) daily steps. We estimated that half of all children (51%) achieved ≥60 minutes of daily MVPA.

Girls engaged less in total physical activity and MVPA, and took fewer steps than boys. They were also more sedentary and were less likely than boys to meet the daily MVPA recommendation (38% versus 63%).

Overall activity levels were lowest in children of Indian origin, who also recorded the least MVPA, were among the most sedentary and took the fewest steps. MVPA levels were lower in girls than in boys across all ethnic groups (Figure 2). Black children were the most likely (57%), and children of Indian origin the least likely (36%), to meet the MVPA recommendation; the difference between these two groups was significant in unadjusted (p=0.013) but not in adjusted analyses (p=0.176).

Children with mothers in lower supervisory and technical occupations had the lowest overall physical activity levels and took the least steps. Children whose mothers were small employers and own account workers recorded the least daily MVPA and were least likely to meet the MVPA recommendation. Conversely, those whose mothers have never worked or had been long-term unemployed were the most likely to meet this recommendation.

Children from couple parent families engaged in less total physical activity and daily MVPA than those whose mothers did not live with partners. They also took the least steps and were least likely to meet the MVPA recommendation. Both groups engaged in similar amounts of sedentary time.

Little variation was found in physical activity levels according to the number of children living in the household, although lone children in the household were slightly more sedentary.

Significant differences between UK countries were found: those living in Northern Ireland engaged in the least total physical activity, daily MVPA and took the

For peer review only - http://bmjopen.bmj.com/site/about/guidelines.xhtml

For peer review only

fewest steps. Children in Northern Ireland and Scotland were more sedentary than those in England and Wales. 52% of children in Wales and England, and 42% in Northern Ireland, achieved the recommended amount of daily MVPA. Though spatial autocorrelation was not statistically significant (I = 0.095, p = 0.249) some interesting regional differences within England were also identified (Figure 3); children in the North West were the most active (60% met the recommended level) and those in the Midlands the least active (47%).

For peer review only

For peer review only - http://bmjopen.bmj.com/site/about/guidelines.xhtml

Table 1: Description of physical activity (PA) measures; weighted median and interquartile range, unless otherwise stated (n = 6497)

	Overall activity (counts/min)	Sedentary (hours/day)	Moderate and vigorous (min/day)	Steps / day	% of children meeting recommended PA (standard error of proportions)
All children	594<br>504, 697	6.5<br>6.0, 7.1	60.6<br>47.2, 76.2	10180<br>8712, 11897	50.9<br>(0.009)
**Child's gender**					
Male	629[a]<br>537, 736	6.4[a]<br>5.8, 6.9	67.4[a]<br>53.4, 84.0	10732[a]<br>9125, 12495	63.2[a]<br>(0.012)
Female	556[a]<br>477, 657	6.7[a]<br>6.1, 7.2	54.0[a]<br>42.7, 67.2	9675[a]<br>8283, 11127	38.1[a]<br>(0.011)
**Child's ethnicity**					
White	597[a,b]<br>506, 702	6.5[a,b]<br>6.0, 7.1	60.6[a]<br>47.4, 76.4	10294[a,b,c,d,e]<br>8828, 12051	51.0[a]<br>(0.009)

For peer review only

Mixed	571[a,c]<br>507, 671	6.8[a,c]<br>6.3, 7.4	62.8[b]<br>51.3, 70.7	9895[f]<br>8553, 11167	54.5<br>(0.040)
Indian	527[b,c,d,e,f]<br>448, 632	6.8[b]<br>6.4, 7.3	52.2[a,b,c,d]<br>40.3, 66.9	8728[a,f]<br>7554, 9991	36.4[a]<br>(0.050)
Pakistani	604[d]<br>501, 693	6.4[c]<br>5.8, 7.0	60.7[c]<br>46.8, 75.2	9441[b]<br>7895, 11212	53.2<br>(0.051)
Bangladeshi	546<br>423, 671	6.5<br>5.8, 7.2	53.2<br>40.1, 74.6	8825[c]<br>6709, 10331	40.7<br>(0.068)
Black	596[e]<br>515, 711	6.5<br>5.9, 7.0	65.7[d]<br>49.4, 86.1	9114[d]<br>8112, 10784	56.6<br>(0.057)
Other	588[f]<br>480, 655	6.5<br>5.9, 7.4	61.4<br>46.1, 74.8	9582[e]<br>8486, 11310	50.9<br>(0.068)
**Maternal socioeconomic circumstances**					
Managerial & professional occupations	587[a]<br>498, 689	6.6<br>6.0, 7.1	59.2[a]<br>46.9, 75.9	10101<br>8655, 11667	48.9[a,b]<br>(0.013)

For peer review only - http://bmjopen.bmj.com/site/about/guidelines.xhtml

Intermediate occupations	591[b] 509, 700	6.6 6.0, 7.1	59.2[b] 46.7, 75.9	10151 8818, 11771	48.8[c,d] (0.018)
Small employers & own account workers	585[e] 499, 672	6.6 6.0, 7.1	58.5[c] 47.6, 69.9	10124 8701, 11561	48.0[e] (0.030)
Lower supervisory & technical occupations	573[c,d] 504, 682	6.6 6.1, 7.1	59.8 48.6, 71.4	9845[a] 8598, 11440	49.9 (0.036)
Semi-routine & routine occupations	608[a,b,c,e] 506, 711	6.5 5.9, 7.0	61.8[a,b,c] 47.1, 77.9	10371[a] 8752, 12181	53.1[a,c] (0.015)
Never worked & long-term unemployed	601[d] 512, 688	6.4 5.8, 6.9	62.8 47.9, 76.6	10112 8593, 12148	56.3[b,d,e] (0.042)
**Lone parenthood status**					
No	589[a] 500, 693	6.5 6.0, 7.1	59.7 [a] 46.6, 75.6	10097[a] 8655, 11795	49.3[a] (0.009)
Yes	607[a] 520, 710	6.5 5.9, 7.0	63.4[a] 49.6, 78.8	10418[a] 8946, 12280	56.6[a] (0.020)
**No. of children in the household**					

For peer review only - http://bmjopen.bmj.com/site/about/guidelines.xhtml

Only child	596<br>499, 691	6.6[a]<br>6.1, 7.2	61.6<br>46.3, 74.8	10155<br>8775, 11962	52.1<br>(0.025)
At least one other child	594<br>505, 699	6.5[a]<br>6.0, 7.1	60.5<br>47.3, 76.3	10182<br>8699, 11892	50.7<br>(0.009)
**Country**					
England	597[a,b]<br>506, 700	6.5[a,b,c]<br>6.0, 7.1	61.0[a,b]<br>47.5, 76.4	10206[a,b]<br>8746, 11949	51.8[a,b]<br>(0.010)
Wales	597[c,d]<br>507, 711	6.4[a,d,e]<br>5.9, 6.9	61.4[c,d]<br>46.4, 77.9	10369[c,d]<br>8791, 12122	52.0[c,d]<br>(0.020)
Scotland	575[a,c]<br>494, 680	6.6[b,d]<br>6.1, 7.1	57.3[a,c]<br>45.8, 73.2	9911[a,c]<br>8563, 11285	44.1[a,c]<br>(0.021)
Northern Ireland	558[b,d]<br>475, 662	6.6[c,e]<br>6.1, 7.1	55.9[b,d]<br>43.1, 72.1	9545[b,d]<br>8048, 11057	41.2[b,d]<br>(0.020)

Table footnote: As there are many multiple comparisons among levels of factors included in the models, this table does not show any *p*-values and only indicates those pairwise differences which were significantly different after applying the Benjamini-Hochberg correction for false discovery rate in multiple comparisons. We have used super indices (a-e) to indicate pairs of groups within a factor that differed significantly ($p<0.05$). Percentage of children meeting recommended PA column: differences are adjusted for gender, ethnicity, maternal socioeconomic circumstances, lone parenthood status, no. of children in the household, and country.

For peer review only - http://bmjopen.bmj.com/site/about/guidelines.xhtml

For peer review only

DISCUSSION

Statement of principal findings

This is the first population-based UK-wide study of objectively measured physical activity and sedentary time in primary-school aged children. We found that when measured in 2008 - 2009 51% of children met the CMO recommendation of 60 minutes or more of daily MVPA.[10,33] More than half of all children spent a substantial part of the day being sedentary.

This study highlights social and demographic variation in UK children's physical activity and inactivity. Girls were more sedentary than boys and less active across all intensities of physical activity. Ethnic differences were observed with children of Indian origin generally the least active across all outcomes and Black children were more likely than other groups to meet the MVPA recommendation. There were no clear socioeconomic gradients in physical activity levels and adherence to guidelines, although some it appeared that less advantaged children were slightly more active than more advantaged children. Children of lone parents were more active and likely to meet the MVPA recommendation than those from couple parent families. There were differences between UK countries across all sedentary and physical activity outcomes with children in Northern Ireland less active and least likely to achieve recommended activity levels than those from other countries. Nevertheless, with the exception of gender, the group differences observed in this study were relatively small and the most striking finding is the low level of activity across all groups.

Strengths and limitations

The strengths of this study include the large UK-wide and representative sample, the use of accelerometers to provide objective measures of physical activity and sedentary time, and the data processing undertaken to exclude any data that might be unreliable. As there is limited consensus on data reduction strategies,[34] most commonly relating to the best threshold with which to define MVPA, we derived the thresholds used in our study from a calibration study we conducted specifically in this age group and consider the thresholds used to be reliable.[23] The

For peer review only - http://bmjopen.bmj.com/site/about/guidelines.xhtml

ability to link accelerometer data to a rich range of social and demographic information has enabled examination, for the first time, of ethnic, social and geographical variations in adherence to policy recommendations. Acknowledged limitations include underestimation, as a result of the accelerometers used, of activities that do not include vertical movement of the trunk, like cycling, and aquatic activities, such as swimming.

Comparison with literature

Findings from large (defined as ≥400 participants) UK-based accelerometry studies reporting adherence to physical activity guidelines are summarised in Table 2.

Table 2: Summary of accelerometry studies in UK-based school-aged children.

Reference and study name / size	Setting	Year measured	Mean age (Years)	Threshold used to define MVPA	Prevalence sufficiently active* (%)	
					Boys	Girls
National Probability Samples						
MCS (current study) (*n*=6497)	England, Scotland, Wales and Northern Ireland	2008-09	7.5	2240 cpm[23]	63	38
HSE 2008[35] (*n*=1286)	England	2008	9.9	2802 cpm[35]	33	21

For peer review only - http://bmjopen.bmj.com/site/about/guidelines.xhtml

Regional Samples						
Basterfield et al[36]<br><br>Gateshead Millennium Study<br><br>(*n*=405)	England (Gateshead)	2006-07 and 2008-09	7.4 and 9.3	3200 cpm[18]	7yrs: 10.1<br>9yrs: 8.6	7yrs: 2.9<br>9yrs: 2.9
Owen et al[37]<br><br>CHASE<br><br>(*n*=2071)	England (London, Birmingham and Leicester)	2006-07	9.9	2000 cpm[17]	76	53
Steele et al[38]<br><br>SPEEDY<br><br>(*n*=1862)	England (Norfolk)	2007	10.2	2000 cpm[17]	81.5	59.4
Gidlow et al[39]	England (Stoke-on-Trent)	2006-07	10.4	Age-specific thresholds[40]	94[40]	89[40]

For peer review only - http://bmjopen.bmj.com/site/about/guidelines.xhtml

CHAMPS UK (*n*=503)				and 3200 cpm[18]	5.2[18]	1.4[18]
Wheeler et al[41] PEACH (*n*= 1053)	England (Bristol)	2006-09	10.9	3200 cpm[18]	6.8	5.3
Riddoch et al[42] ALSPAC (*n*=5595)	England (Bristol)	2003-05	11	3600 cpm[43]	5.1	0.4

Table footnote: *Accumulating ≥60 min/day MVPA. Table abbreviations: MCS: Millennium Cohort Study; ALSPAC: Avon Longitudinal Study of Parents and Children; SPEEDY: Sport, Physical Activity and Eating Behaviour, Environmental Determinants in Young People; CHASE: Child Heart and Health Study; CHAMPS: Children's Health and Activity Monitoring Programme in Schools; PEACH project: Personal and Environmental Associations with Children's Health; cpm, counts per minute.

For peer review only - http://bmjopen.bmj.com/site/about/guidelines.xhtml

In none were children measured in population-based samples from all four UK countries. The Millennium Gateshead Study measured children of a similar age to those in our study, although in a much smaller sample. The results show wide variation in the proportions reported to adhere to recommended guidelines on activity but are consistent with our finding that boys are more active than girls. These differences may in part be due to the use of different MVPA intensity thresholds, ranging from 2000 to 3600 cpm. We examined this by recalculating our data using different thresholds: the percentage of children meeting recommendations increased to 80.4% (standard error of proportion: 0.010) and 59.4% (0.011) for boys and girls, respectively, when applying a threshold of 2000 cpm and fell to 13.7% (0.008) and 0.4% (0.005) when applying a higher threshold of 3000 cpm. Clearly, therefore, our estimates are similar to some of these other studies[37;38] when a similar threshold of 2000 cpm is applied. However, other differences may reflect variation in regions, sampling methods and settings for recruitment as well as in approaches to adjustment for missing data. Further work is needed to clearly define the best thresholds for different intensities of activity in children incorporating novel statistical techniques.[44] Clarity over thresholds will be important for routine surveillance measures to monitor the achievement of policy objectives relating to physical activity.

Only one other large-scale study in the UK has examined ethnic differences in objectively measured physical activity. Owen et al[37] reported lower levels in South Asian children than European whites and black African-Caribbeans, with 54%, 70% and 69% respectively meeting the recommendations. We also found that children of Indian origin were the least, and those of Black ethnic origin among the most, likely to meet these recommendations. Although activity levels differed between children from different socioeconomic circumstances, with those less advantaged slightly more active, we did not find a strong social gradient; this is consistent with other published evidence.[42;45] This is the only study to report country and regional differences across the UK and so direct comparisons with other studies cannot be made.

For peer review only - http://bmjopen.bmj.com/site/about/guidelines.xhtml

The children in our study engaged in slightly less sedentary time than observed in older children in other UK-based accelerometry studies. For example, in the Avon Longitudinal Study of Parents and Children (ALSPAC) 7.0 and 7.3 hours of daily sedentary time were reported in 12-year old boys and girls respectively, increasing at older ages.[46] In a cross sectional study of English ten year olds, Steele et al[38] reported 7.5 and 7.7 hours for boys and girls, respectively.

Tudor-Locke et al[47] recommend that school-aged boys and girls should be encouraged to undertake 13,000-15,000 and 11,000-12,000 steps/day, respectively. The children in this study accumulated fewer steps than this (boys: 10732; girls: 9675); however, our estimates are very similar to those reported by Owen et al[37] (boys: 10570; girls: 9123) who also reported higher step numbers in white compared to black and Asian children, which is consistent with our findings.

Unanswered questions and future research

We are currently examining influences on physical activity and sedentary time in this representative cohort of school-aged children in more detail using longitudinal data to examine their prior determinants. Once follow up data become available from subsequent contacts with cohort members we propose to examine the consequences for health in later childhood and adolescence. Objective measures of physical activity and sedentary time can allow these influences to be characterised with greater precision and to be compared with subjective and proxy reports of engagement in physical activity and sedentary behaviours, also collected within this cohort. We have documented the accelerometer data and derived variables and are making these accessible to other researchers through the UK Data Archive.

Implications for practice and policy

The London 2012 Olympic Games promised to inspire a generation to take part in sport. The UK success in August provides a platform for the Government's plans for an Olympic and Paralympic sporting legacy,[48] to encourage and enable pupils to engage in competitive sports and activities. The results of our study provide a useful baseline and emphasise that

For peer review only - http://bmjopen.bmj.com/site/about/guidelines.xhtml

For peer review only

contemporary UK children are insufficiently active, implying that effort is needed to boost physical activity among young people to the level appropriate for good health. This is likely to require population-wide interventions across the range of physical activity domains. There are many opportunities to increase physical activity through sport but also in other areas.[49] The journey to school has been recognised as an important opportunity for increasing total as well as more intense physical activity. Urban environments are also important for children's physical activity,[41] and factors from available green space to perceptions of safety all impact on children’s activity. The full potential to boost physical activity will only be realised with a comprehensive policy response that increases time spent in more intense physical activity and decreases the time spent being sedentary. Investing in this area is a vital component to deliver the Olympic legacy and improve the short and long-term health of our children.

For peer review only

For peer review only - http://bmjopen.bmj.com/site/about/guidelines.xhtml

Declaration of interests: None

Authors Contributions

CR, MG, MCB, LJG, CD, FS and TP contributed to data processing. TP and MCB conducted the data analysis and, with LJG and CD, interpreted the data and drafted the article. All authors contributed to the study conception/design, critically revised the article and reviewed the final draft of the article. LJG is guarantor.

Acknowledgements

The Centre for Paediatric Epidemiology and Biostatistics is supported in part by the Medical Research Council in its capacity as the MRC Centre of Epidemiology for Child Health (Grant reference G0400546). Research at the UCL Institute of Child Health and Great Ormond Street Hospital for Children receives a proportion of the funding from the Department of Health's National Institute for Health Research Biomedical Research Centres funding scheme. The fourth sweep of the Millennium Cohort Study was funded by grants to Professor Health Joshi, former director of the study, from the Economic and Social Research Council and a consortium of government funders. The current director is Professor Lucinda Platt. The co-operation of the participating families is gratefully acknowledged. Accelerometer data collection was funded by the Wellcome Trust (principal investigator Professor Carol Dezateux, grant 084686/Z/08/A). The study sponsors played no part in the design, data analysis and interpretation of this study, the writing of the manuscript, or the decision to submit the paper for publication and the authors' work was independent of their funders.

Data sharing statement:

The MCS data for surveys 1 to 4 are currently available via the Economic and Social Data Service; the MCS accelerometer data will also shortly be made available.

For peer review only

FIGURES

Figure 1 Flow diagram of study participants

Figure 2 Median minutes per day of moderate to vigorous physical activity by gender (1: boys; 2: girls) and ethnicity

Figure 3: Children meeting physical activity recommendation of ≥60 min/day MVPA (%)

For peer review only

For peer review only - http://bmjopen.bmj.com/site/about/guidelines.xhtml

Reference List

(1) Hills AP, Andersen LB, Byrne NM. Physical activity and obesity in children. *Br J Sports Med* 2011; 45(11):866-870.

(2) Owen CG, Nightingale CM, Rudnicka AR, Sattar N, Cook DG, Ekelund U et al. Physical activity, obesity and cardiometabolic risk factors in 9- to 10-year-old UK children of white European, South Asian and black African-Caribbean origin: the Child Heart And health Study in England (CHASE). *Diabetologia* 2010; 53(8):1620-1630.

(3) Tobias JH, Steer CD, Mattocks CG, Riddoch C, Ness AR. Habitual levels of physical activity influence bone mass in 11-year-old children from the United Kingdom: findings from a large population-based cohort. *J Bone Miner Res* 2007; 22(1):101-109.

(4) Ekeland E, Heian F, Hagen KB. Can exercise improve self esteem in children and young people? A systematic review of randomised controlled trials. *Br J Sports Med* 2005; 39(11):792-798.

(5) Ekelund U, Brage S, Froberg K, Harro M, Anderssen SA, Sardinha LB et al. TV viewing and physical activity are independently associated with metabolic risk in children: the European Youth Heart Study. *PLoS Med* 2006; 3(12):e488.

(6) Kwon S, Janz KF. Tracking of accelerometry-measured physical activity during childhood: ICAD pooled analysis. *Int J Behav Nutr Phys Act* 2012; 9(1):68.

(7) Biddle SJ, Pearson N, Ross GM, Braithwaite R. Tracking of sedentary behaviours of young people: a systematic review. *Prev Med* 2010; 51(5):345-351.

(8) U.S.Department of Health and Human Services. 2008 Physical Activity Guidelines for Americans. 2008. Washington, U.S. Department of Health and Human Services.

(9) Tremblay MS, Warburton DER, Janssen I, Paterson DH, Latimer AE, Rhodes RE et al. New Canadian Physical Activity Guidelines. *Appl Physiol Nutr Metab* 2011; 36(1):36-46.

(10) Department of Health. Start active, stay active: a report on physical activity from the four home countries' Chief Medical Officers. 2011. London: Department of Health.

(11) Reilly JJ, Ness AR, Sherriff A. Epidemiological and physiological approaches to understanding the etiology of pediatric obesity: finding the needle in the haystack. *Pediatr Res* 2007; 61(6):646-652.

(12) Troiano RP. Large-scale applications of accelerometers: new frontiers and new questions. *Med Sci Sports Exerc* 2007; 39(9):1501.

(13) Trost SG, McIver KL, Pate RR. Conducting accelerometer-based activity assessments in field-based research. *Med Sci Sports Exerc* 2005; 37(11 Suppl):S531-S543.

(14) Hamer M, Fisher A. Are interventions to promote physical activity in children a waste of time? *BMJ* 2012; 345:e6320.

(15) Hansen K. Millennium Cohort Study First, Second, Third and Fourth Surveys: A Guide to the Datasets (Sixth Edition). 2012. London, Centre for Longitudinal Studies, University of London.

For peer review only - http://bmjopen.bmj.com/site/about/guidelines.xhtml

(16) Ott AE, Pate RR, Trost SG, Ward DS, Saunders R. The use of uniaxial and triaxial accelerometers to measure children's "free-play" physical activity. *Pediatric Exercise Science* 2000; 12(4):360-370.

(17) Trost SG, Ward DS, Moorehead SM, Watson PD, Riner W, Burke JR. Validity of the computer science and applications (CSA) activity monitor in children. *Med Sci Sports Exerc* 1998; 30(4):629-633.

(18) Puyau MR, Adolph AL, Vohra FA, Butte NF. Validation and calibration of physical activity monitors in children. *Obes Res* 2002; 10(3):150-157.

(19) Ekelund U, Yngve A, Brage S, Westerterp K, Sjostrom M. Body movement and physical activity energy expenditure in children and adolescents: how to adjust for differences in body size and age. *Am J Clin Nutr* 2004; 79(5):851-856.

(20) Geraci M. 2012. pawacc: Physical activity with accelerometers. R package version 1.0. http://r-forge r-project org/projects/pawacc/

(21) Geraci M, Rich C, Sera F, Cortina-Borja M, Griffiths LJ, Dezateux C. 2012. Technical report on accelerometry data processing in the Millennium Cohort Study. http://discovery ucl ac uk/1361699/

(22) Rich C, Cortina-Borja M, Dezateux C, Geraci M, Sera F, Calderwood C et al. Predictors of non-response in a UK-wide cohort study of children's accelerometer-determined physical activity using postal methods. *BMJ Open* 2013; 3 (issue 3).

(23) Pulsford RM, Cortina-Borja M, Rich C, Kinnafick FE, Dezateux C, Griffiths LJ. Actigraph accelerometer-defined boundaries for sedentary behaviour and physical activity intensities in 7 year old children. *PLoS One* 2011; 6(8):e21822.

(24) Office for National Statistics. Ethnic Group Statistics: A Guide for the Collection and Classification of Ethnicity Data. 2003. London, The Stationery Office.

(25) Rose D, Pevalin D. A Researcher's Guide to the National Statistics Socio-economic Classification. 2003. London, Sage Publications.

(26) R Core Team. R: A language and environment for statistical computing. Vienna, Austria.: R Foundation for Statistical Computing; 2012.

(27) Geraci M. Estimation of regression quantiles in complex surveys with data missing at random: An application to birthweight determinants. Statistical Methods in Medical Research 2013; in press.

(28) Koenker R. 2012. Quanteg: Quantile Regression R package version 4.79. http://CRAN R-project org/package=quanteg

(29) Benjamini Y, Hochberg Y. Controlling the False Discovery Rate: A Practical and Powerful Approach to Multiple Testing. *Journal of the Royal Statistical Society Series B (Methodological)* 1995; 57(1):289-300.

(30) Pollard KS, Gilbert HN, Ge Y, Taylor S, Dudoit S. Multtest: Resampling-based multiple hypothesis testing. 2012. R package version 2.12.0. [Accessed January 2013]

(31) Cliff AD, Ord JK. Spatial processes. London: Pion; 1981.

For peer review only - http://bmjopen.bmj.com/site/about/guidelines.xhtml

(32) Anselin L. Exploratory spatial data analysis and geographic information systems. In: Longley P, Brooks S, Macmillan B, McDonnell R, editors. GeoComputation: A primer. New York: Willey; 1994. 77-94.

(33) Department of Health. At least 5 a week: physical activity and health outcomes: a review of the Chief Medical Officer. 2004. London, Department of Health.

(34) Ekelund U, Tomkinson G, Armstrong N. What proportion of youth are physically active? Measurement issues, levels and recent time trends. *Br J Sports Med* 2011; 45(11):859-865.

(35) The NHS Information Centre. The Health Survey for England – 2008: Physical Activity and Fitness. 2009.

(36) Basterfield L, Adamson AJ, Frary JK, Parkinson KN, Pearce MS, Reilly JJ. Longitudinal study of physical activity and sedentary behavior in children. *Pediatrics* 2011; 127(1):e24-e30.

(37) Owen CG, Nightingale CM, Rudnicka AR, Cook DG, Ekelund U, Whincup PH. Ethnic and gender differences in physical activity levels among 9-10-year-old children of white European, South Asian and African-Caribbean origin: the Child Heart Health Study in England (CHASE Study). *Int J Epidemiol* 2009; 38(4):1082-1093.

(38) Steele RM, van Sluijs EM, Cassidy A, Griffin SJ, Ekelund U. Targeting sedentary time or moderate- and vigorous-intensity activity: independent relations with adiposity in a population-based sample of 10-y-old British children. *Am J Clin Nutr* 2009; 90(5):1185-1192.

(39) Gidlow CJ, Cochrane T, Davey R, Smith H. In-school and out-of-school physical activity in primary and secondary school children. *J Sports Sci* 2008; 26(13):1411-1419.

(40) Trost SG, Pate RR, Sallis JF, Freedson PS, Taylor WC, Dowda M et al. Age and gender differences in objectively measured physical activity in youth. *Med Sci Sports Exerc* 2002; 34(2):350-355.

(41) Wheeler BW, Cooper AR, Page AS, Jago R. Greenspace and children's physical activity: a GPS/GIS analysis of the PEACH project. *Prev Med* 2010; 51(2):148-152.

(42) Riddoch CJ, Mattocks C, Deere K, Saunders J, Kirkby J, Tilling K et al. Objective measurement of levels and patterns of physical activity. *Arch Dis Child* 2007; 92(11):963-969.

(43) Mattocks C, Leary S, Ness A, Deere K, Saunders J, Tilling K et al. Calibration of an accelerometer during free-living activities in children. *Int J Pediatr Obes* 2007; 2(4):218-226.

(44) Crouter SE, Clowers KG, Bassett DR, Jr. A novel method for using accelerometer data to predict energy expenditure. *J Appl Physiol* 2006; 100(4):1324-1331.

(45) Ruiz JR, Ortega FB, Martinez-Gomez D, Labayen I, Moreno LA, De B, I et al. Objectively measured physical activity and sedentary time in European adolescents: the HELENA study. *Am J Epidemiol* 2011; 174(2):173-184.

(46) Mitchell JA, Pate RR, Dowda M, Mattocks C, Riddoch C, Ness AR et al. A Prospective Study of Sedentary Behavior in a Large Cohort of Youth. *Med Sci Sports Exerc* 2011.

For peer review only - http://bmjopen.bmj.com/site/about/guidelines.xhtml

(47) Tudor-Locke C, Craig CL, Beets MW, Belton S, Cardon GM, Duncan S et al. How many steps/day are enough? for children and adolescents. *Int J Behav Nutr Phys Act* 2011; 8:78.

(48) Department of Culture MaS. Beyond 2012 – The London 2012 Legacy Story. 2012. London, Department of Culture, Media and Sport.

(49) NICE. Promoting physical activity, active play and sport for pre-school and school-age children and young people in family, pre-school, school and community settings. 2009. London, NICE.

For peer review only

For peer review only - http://bmjopen.bmj.com/site/about/guidelines.xhtml

eTable1 Description of sample; percentages, unless otherwise stated

	All singleton children in MCS4 N (weighted %)			Singleton children with reliable accelerometer data (≥2 days for at least ≥10 hours per day) N (weighted %)		
	Male n=6950	Female n=6731	Total n=13681	Male n=3176	Female n=3321	Total n=6497
Child's ethnic group						
White	5828 (85.6)	5610 (85.3)	11438 (85.4)	2795 (85.0)	2915 (85.3)	5710 (85.2)
Mixed	178 (3.1)	194 (3.3)	372 (3.3)	85 (3.5)	83 (3.0)	168 (3.2)
Indian	179 (2.1)	162 (1.8)	341 (1.9)	61 (1.8)	78 (2.2)	139 (2.0)
Pakistani	308 (3.4)	321 (3.7)	629 (3.5)	93 (4.2)	84 (3.4)	177 (3.8)
Bangladeshi	113 (1.1)	141 (1.3)	254 (1.2)	30 (1.2)	40 (1.4)	70 (1.3)
Black	221 (3.2)	193 (2.8)	414 (3.0)	71 (2.9)	71 (2.8)	142 (2.8)
Other ethnic group	121 (1.6)	106 (1.7)	227 (1.6)	41 (1.4)	49 (1.9)	90 (1.6)
Maternal socioeconomic circumstances						
Managerial and professional occupations	1942 (28.1)	1906 (29.6)	3848 (28.8)	1098 (28.2)	1092 (27.8)	2190 (28.0)

For peer review only

Intermediate occupations	1229 (18.7)	1115 (17.9)	2344 (18.3)	592 (18.6)	610 (17.9)	1202 (18.3)
Small employers and own account workers	422 (7.0)	403 (6.5)	825 (6.7)	217 (7.0)	225 (6.7)	442 (6.8)
Lower supervisory and technical occupations	293 (4.5)	296 (4.6)	589 (4.6)	138 (5.0)	150 (5.5)	288 (5.2)
Semi-routine and routine occupations	2263 (35.8)	2197 (34.8)	4460 (35.3)	893 (34.7)	972 (35.2)	1865 (34.9)
Never worked and long-term unemployed	426 (6.0)	466 (6.6)	892 (6.3)	118 (6.5)	150 (7.0)	268 (6.7)
Lone parenthood						
No	5530 (77.9)	5375 (78.9)	10905 (78.4)	2718 (78.3)	2818 (77.7)	5536 (78.0)
Yes	1420 (22.1)	1356 (21.1)	2776 (21.6)	458 (21.7)	503 (22.3)	961 (22.0)
No. children in the household						
Only child	903 (13.2)	868 (13.4)	1771 (13.3)	340 (11.7)	386 (13.0)	726 (12.4)
At least one other child	6047 (86.8)	5863 (86.6)	11910 (86.7)	2836 (88.3)	2935 (87.0)	5771 (87.6)
Country of residence						
England	4451 (63.9)	4335 (64.6)	8786 (64.2)	2064 (64.7)	2140 (65.2)	4204 (65.0)

For peer review only

Wales	1015 (14.8)	930 (13.6)	1945 (14.2)	450 (14.8)	448 (13.3)	898 (14.0)
Scotland	809 (11.3)	790 (12.0)	1599 (11.6)	363 (11.1)	398 (11.9)	761 (11.5)
Northern Ireland	675 (10.0)	676 (9.8)	1351 (9.9)	299 (9.4)	335 (9.6)	634 (9.5)

Missing data: Child's ethnic group, 6 for all children in MCS4 and 1 for children with activity data; Maternal socioeconomic class, 723 for all children in MCS4 and 259 for children with activity data.

For peer review only

Figure 1 Flow diagram of study participants

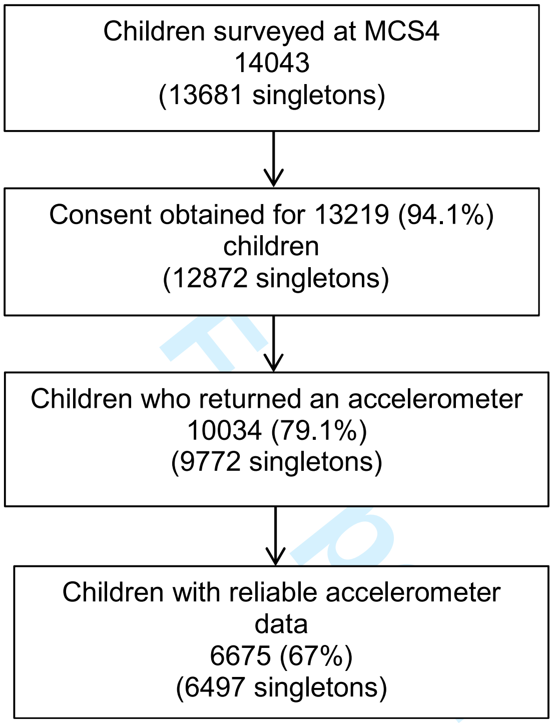

For peer review only

Figure 2

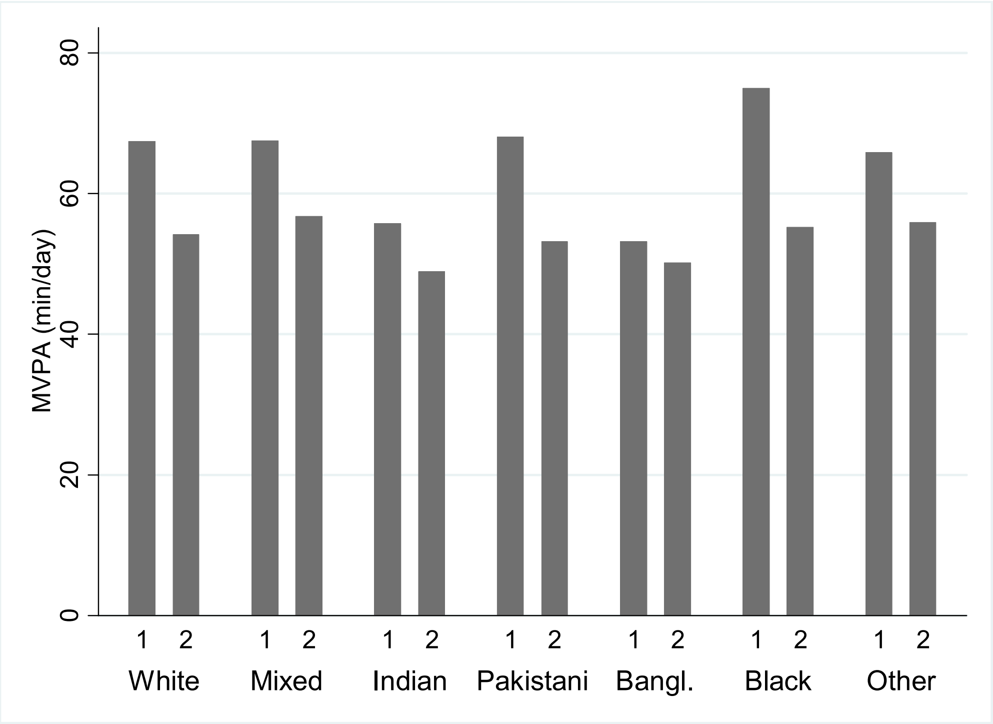

For peer review only

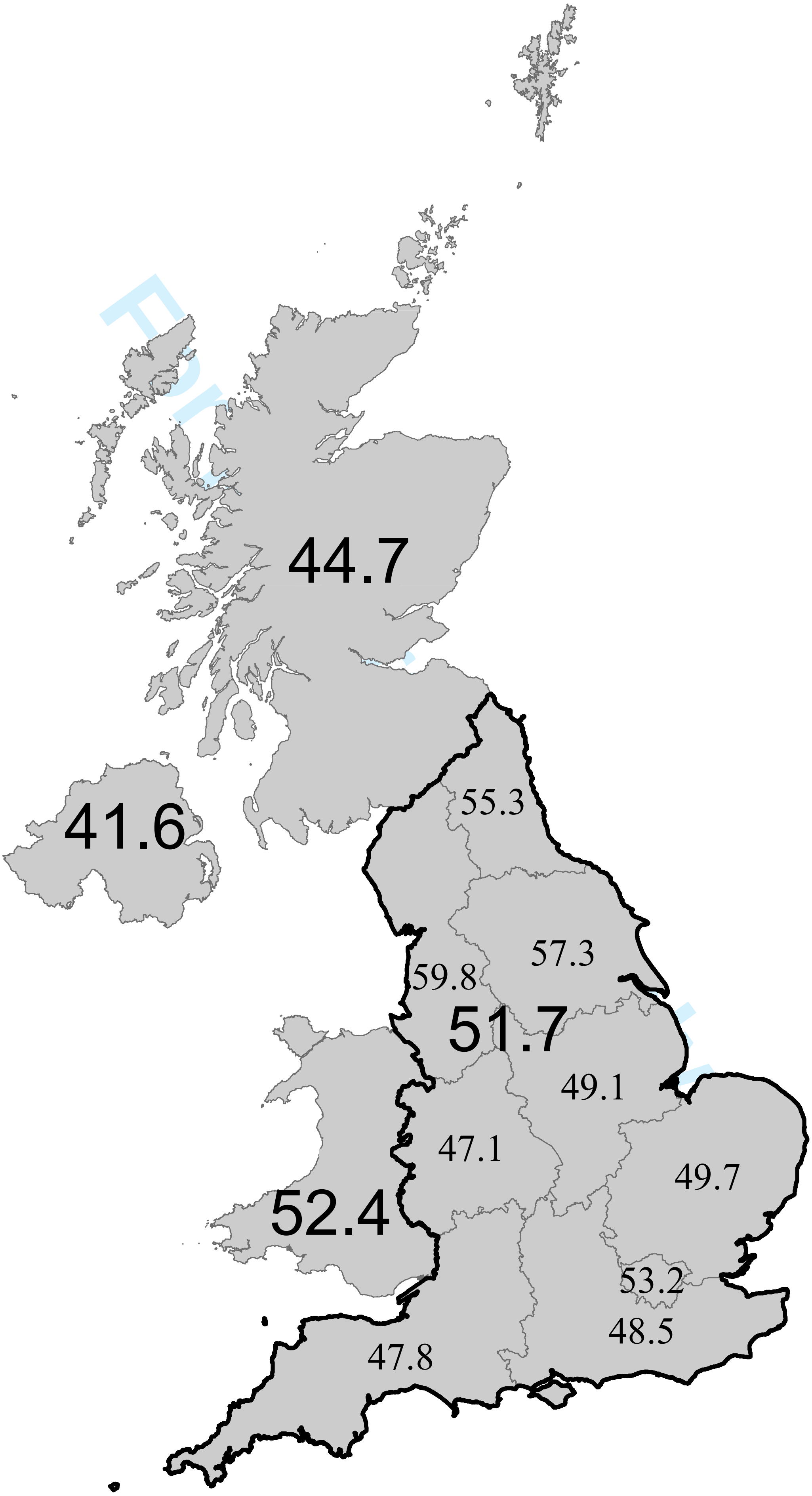
44.7
41.6
55.3
57.3
59.8
51.7
49.1
47.1
49.7
52.4
53.2
48.5
47.8

STROBE Statement—Checklist of items that should be included in reports of ***cohort studies***

	Item No	Recommendation
**Title and abstract**	1 √	(*a*) Indicate the study's design with a commonly used term in the title or the abstract
		(*b*) Provide in the abstract an informative and balanced summary of what was done and what was found
**Introduction**		
Background/rationale	2 √	Explain the scientific background and rationale for the investigation being reported
Objectives	3 √	State specific objectives, including any prespecified hypotheses
**Methods**		
Study design	4 √	Present key elements of study design early in the paper
Setting	5 √	Describe the setting, locations, and relevant dates, including periods of recruitment, exposure, follow-up, and data collection
Participants	6 √	(*a*) Give the eligibility criteria, and the sources and methods of selection of participants. Describe methods of follow-up
		(*b*) For matched studies, give matching criteria and number of exposed and unexposed
Variables	7 √	Clearly define all outcomes, exposures, predictors, potential confounders, and effect modifiers. Give diagnostic criteria, if applicable
Data sources/ measurement	8* √	For each variable of interest, give sources of data and details of methods of assessment (measurement). Describe comparability of assessment methods if there is more than one group
Bias	9 √	Describe any efforts to address potential sources of bias
Study size	10 √	Explain how the study size was arrived at
Quantitative variables	11 √	Explain how quantitative variables were handled in the analyses. If applicable, describe which groupings were chosen and why
Statistical methods	12 √	(*a*) Describe all statistical methods, including those used to control for confounding
		(*b*) Describe any methods used to examine subgroups and interactions
		(*c*) Explain how missing data were addressed
		(*d*) If applicable, explain how loss to follow-up was addressed
		(*e*) Describe any sensitivity analyses
**Results**		
Participants	13* √	(a) Report numbers of individuals at each stage of study—eg numbers potentially eligible, examined for eligibility, confirmed eligible, included in the study, completing follow-up, and analysed
		(b) Give reasons for non-participation at each stage
		(c) Consider use of a flow diagram
Descriptive data	14* √	(a) Give characteristics of study participants (eg demographic, clinical, social) and information on exposures and potential confounders
		(b) Indicate number of participants with missing data for each variable of interest
		(c) Summarise follow-up time (eg, average and total amount)
Outcome data	15* √	Report numbers of outcome events or summary measures over time
Main results	16 √	(*a*) Give unadjusted estimates and, if applicable, confounder-adjusted estimates and their precision (eg, 95% confidence interval). Make clear which confounders were adjusted for and why they were included
		(*b*) Report category boundaries when continuous variables were categorized
		(*c*) If relevant, consider translating estimates of relative risk into absolute risk for a meaningful time period

For peer review only - http://bmjopen.bmj.com/site/about/guidelines.xhtml
For peer review only

Other analyses	17	Report other analyses done—eg analyses of subgroups and interactions, and sensitivity analyses
**Discussion**		
Key results	18 √	Summarise key results with reference to study objectives
Limitations	19 √	Discuss limitations of the study, taking into account sources of potential bias or imprecision. Discuss both direction and magnitude of any potential bias
Interpretation	20 √	Give a cautious overall interpretation of results considering objectives, limitations, multiplicity of analyses, results from similar studies, and other relevant evidence
Generalisability	21 √	Discuss the generalisability (external validity) of the study results
**Other information**		
Funding	22 √	Give the source of funding and the role of the funders for the present study and, if applicable, for the original study on which the present article is based

*Give information separately for exposed and unexposed groups.

Note: An Explanation and Elaboration article discusses each checklist item and gives methodological background and published examples of transparent reporting. The STROBE checklist is best used in conjunction with this article (freely available on the Web sites of PLoS Medicine at http://www.plosmedicine.org/, Annals of Internal Medicine at http://www.annals.org/, and Epidemiology at http://www.epidem.com/). Information on the STROBE Initiative is available at http://www.strobe-statement.org.

For peer review only

For peer review only - http://bmjopen.bmj.com/site/about/guidelines.xhtml

How active are our children? Findings from the Millennium Cohort Study

Journal:	*BMJ Open*
Manuscript ID:	bmjopen-2013-002893.R1
Article Type:	Research
Date Submitted by the Author:	10-Jun-2013
Complete List of Authors:	Griffiths, Lucy; UCL Institute of Child Health, MRC Centre of Epidemiology for Child Health<br>Cortina Borja, Mario; UCL Institute of Child Health,<br>Sera, Francesco; UCL Institute of Child Health,<br>Pouliou, Theodora; UCL Institute of Child Health,<br>Geraci, Marco; UCL Institute of Child Health,<br>Rich, Carly; UCL Institute of Child Health,<br>Cole, Tim; UCL Institute of Child Health,<br>Law, Catherine; UCL Institute of Child Health,<br>Joshi, Heather; Institute of Education,<br>Ness, Andy; School of Oral Dental Science,<br>Jebb, Susan; MRC Human Nutrition Research<br>Dezateux, Carol; Institute of Child Health, MRC Centre of Epidemiology for Child Health
**Primary Subject Heading**:	Epidemiology
Secondary Subject Heading:	Public health, Sports and exercise medicine, Paediatrics
Keywords:	PUBLIC HEALTH, SPORTS MEDICINE, PAEDIATRICS

SCHOLARONE™
Manuscripts

How active are our children? Findings from the Millennium Cohort Study

Lucy J Griffiths (senior research fellow)[1], Mario Cortina-Borja (senior lecturer)[1], Francesco Sera (senior research associate)[1], Theodora Pouliou (research associate)[1], Marco Geraci (lecturer)[1], Carly Rich (research associate)[1], Tim J Cole (professor)[1], Catherine Law (professor)[1], Heather Joshi (professor)[2], Andrew R Ness (professor)[3], Susan A Jebb (head of diet and population health)[4] and Carol Dezateux (professor)[1]

[1] MRC Centre of Epidemiology for Child Health, UCL Institute of Child Health, 30 Guilford Street, London, WC1N 1EH.

[2] Quantitative Social Science, Institute of Education, University of London, 20 Bedford Way, London, WC1H 0AL.

[3] School of Oral and Dental Sciences, Bristol Dental School, Lower Maudlin Street, Bristol BS1 2LY.

[4] MRC Human Nutrition Research, Elsie Widdowson Laboratory, 120 Fulbourn Road, Cambridge, CB1 9NL.

Corresponding author: Lucy Griffiths; address as above; lucy.griffiths@ucl.ac.uk

Study design: Prospective cohort study

Keywords: physical activity, sedentary, accelerometry, children, cohort study

For peer review only

ABSTRACT

Objective To describe levels of physical activity, sedentary time and adherence to Chief Medical Officers (CMO) physical activity guidelines among primary school-aged children across the UK using objective accelerometer-based measurements.

Design Nationally representative prospective cohort study.

Setting Children born across the UK, between 2000 and 2002.

Participants 6497 7-8 year old singleton children for whom reliable accelerometer data were available for at least 10 hours a day for at least two days

Main outcome measures Physical activity in counts per minute (cpm); time spent in sedentary and moderate to vigorous intensity physical activity (MVPA); proportion of children meeting CMO guidelines (≥60 min/day MVPA); average daily steps.

Explanatory measures: Gender, ethnicity, maternal current / most recent occupation, lone parenthood status, number of children in the household, and country / region of residence.

Results The median daily physical activity level was 595 cpm (interquartile range (IQR): **507** ~~504~~, 697). Children spent a median of 60 minutes (IQR: 47-76) in MVPA per day and were sedentary for a median of six and a half hours per day (IQR: 6-7). Only 51% met CMO guidelines, with girls (38%) less active than boys (63%). Children took an average of 10229 (95% CI (8777,11775)) steps each day. Children of Indian ethnicity were significantly less active overall than all other ethnic groups. Children of Bangladeshi origin and those living in Northern Ireland were least likely to meet CMO guidelines.

Conclusions Only half of seven year old children in the UK achieve recommended levels of physical activity, with significant gender, ethnic and geographic variations. Longitudinal studies are needed to better understand the relevance of these (in)activity patterns for long term health and well-being. In the meantime population-wide efforts to boost physical activity among young people are needed which are likely to require a broad range of policy interventions.

For peer review only

For peer review only - http://bmjopen.bmj.com/site/about/guidelines.xhtml

ARTICLE SUMMARY

Article focus

- This paper describes levels of physical activity, sedentary time and adherence to Chief Medical Officers (CMO) physical activity guidelines among primary school-aged children across the UK using objective accelerometer-based measurements.

Key messages

- 51% of seven year old UK children achieve current recommendations for daily physical activity; this is significantly lower in girls (38%) than in boys (63%). This is also lowest in children living in Northern Ireland.
- Half of all UK seven year olds are sedentary for six and a half hours or more each day.
- Social and demographic variations in physical (in) activity levels are otherwise smaller.
- A comprehensive policy response is needed to boost physical activity and decrease sedentary time among all young children to the levels appropriate for good health.

Strengths and limitations of this study

- This is the first UK-wide study of children's objectively measured physical (in) activity.
- Linkage of the accelerometer data to a range of social and demographic information enabled examination of ethnic, social and geographical variations in activity levels and adherence to policy recommendations.
- While accelerometers provide higher levels of measurement precision relative to self-report methods of physical activity, they can underestimate certain activities like swimming.

For peer review only

For peer review only - http://bmjopen.bmj.com/site/about/guidelines.xhtml

- The prevalence of adherence to current recommendations for physical activity is sensitive to the thresholds used to define moderate and vigorous physical activity.

For peer review only

For peer review only - http://bmjopen.bmj.com/site/about/guidelines.xhtml

INTRODUCTION

The physical and psychological benefits of physical activity for children and adolescents include reduced adiposity[1] and cardiometabolic risk factors,[2] improvements in musculoskeletal health[3] and psychological well-being.[4] Conversely, and independently of physical activity levels, high levels of sedentary behaviours such as TV viewing may also predict a poor cardiometabolic risk profile.[5] As both physical activity and sedentary behaviour track over time,[6;7] children with low levels of activity may be at risk of continuing at this level in later life.

Guidelines for physical activity in children have recently been revised in the United States,[8] Canada[9] and the United Kingdom (UK).[10] The latter were launched in July 2011 as a UK-wide consensus from the four nations' Chief Medical Officers (CMO) on the amount and type of physical activity for good health at each stage of the lifecourse.[10] It is now recommended that all young people engage in moderate to vigorous intensity physical activity (MVPA) (Box) for at least 60 minutes and up to several hours every day. A greater emphasis is now placed on vigorous intensity activities and, for the first time, it is recommended that extended sedentary time should be reduced (although a daily limit is not specified).

Box Examples of sedentary, moderate and vigorous intensity physical activities

Activity Level	Age-adjusted Metabolic equivalent (MET)*	Examples[10]
Sedentary	1.0 - 1.5	Lying, sitting, watching TV, playing video games
Moderate	3.0 – 5.9 METs	Bike riding and playground activities. These activities will cause children to get warmer and

For peer review only - http://bmjopen.bmj.com/site/about/guidelines.xhtml

		breathe harder and their hearts to beat faster, but they should still be able to carry on a conversation
Vigorous	6.0 - 8.9 METs	Fast running and swimming. These activities will cause children to get warmer, breathe much harder and their hearts to beat rapidly, making it more difficult to carry on a conversation.

*MET: metabolic equivalent; 1 MET is defined as the energy expenditure of sitting quietly, equivalent in children to a resting oxygen intake of ~6 $mL.kg^{-1}.min^{-1}$ [11]

The development of accelerometer-based activity monitors has enabled the frequency, intensity and duration of free-living activity to be measured objectively in large scale population studies,[12-14] documenting the proportion of children meeting recommended guidelines.[15] To date however, accelerometers have not been used in a representative sample of children from all four UK countries.

We report on the time spent in physical activity and sedentary behaviour in a UK-wide representative sample of primary school-aged children participating in a cohort study. We also evaluate adherence to CMO guidelines and variation according to a range of socio-demographic characteristics.

METHODS

The Millennium Cohort Study (MCS) is a prospective study of the social, economic and health-related circumstances of children born in the UK between September 2000 and January 2002.[16] The original cohort comprised 18,818 children (72% of those approached) whose parents were first interviewed at home when their child was aged nine months. Since then data have been collected when the children were aged three, five and seven years, with follow-up currently being conducted at 11 years and planned for 14 years and beyond. This study received research ethics

For peer review only - http://bmjopen.bmj.com/site/about/guidelines.xhtml

approval from the Northern and Yorkshire Research Ethics Committee (07/MRE03/32).

Physical activity data

Physical activity and sedentary time were measured using the Actigraph GT1M accelerometer (Actigraph, Pensacola, Florida), which has been demonstrated to measure physical activity reliably in children when compared to measures derived from heart rate monitoring,[17] indirect and room calorimetry [18;19] and doubly labelled water.[20]

A total of 14,043 children (13,681 singletons) were interviewed at age seven years and invited to participate in the accelerometry study. Those who consented were subsequently sent an accelerometer in the post, programmed to use a 15 second sampling epoch and to record activity as counts and steps. Participants were instructed to start wearing their accelerometer, on an elastic belt round the waist, the morning after they received it and to continue doing so during waking hours for seven consecutive days. They were asked to remove the accelerometer when bathing or during other aquatic activities.

Data were collected between May 2008 and August 2009. Accelerometers were returned by 9772 singleton children. Data were downloaded using Actigraph software version 3.8.3 (Actigraph, Pensacola, Florida) and subsequently processed in-house,[21] according to pre-determined processing criteria.[22] Non-wear time, defined as time periods of consecutive zero activity counts of 20 minutes or more, was excluded from analysis. Extreme count values above a threshold of ≥11,715 cpm were removed. A study was performed to discriminate between days when the accelerometer was worn and days when it was likely not worn (e.g., during postal delivery) on the basis of the minimum number of valid minutes per day. The threshold

For peer review only - http://bmjopen.bmj.com/site/about/guidelines.xhtml

was set to ≥ 150 minutes per day. The first day with registered time exceeding the threshold was set as start date. The last day with registered time above the threshold was set as end date.[22] Participants with registered time of ≥10 hours on at least ≥2 days were included in analyses; only days with ≥10 hours of recorded time were included as this has been shown to produce reliable estimates of PA.[23] This resulted in a final sample size of 6497 singleton children (3176 boys) (Figure 1) with a total of 36,309 days (nearly 449000 hours) of observation.

Small differences were found in the demographic characteristics of the sample of children with reliable accelerometer data relative to the whole cohort sample (*n*=13681) interviewed at age seven years (eTable1). In particular, boys, children living in Wales, those belonging to the Indian, Pakistan/Bangladeshi, and Black Caribbean/African ethnic groups, as well as children belonging to families with a single respondent at the interview showed a lower probability of having a valid accelerometer file. Information on predictors of reliable data acquisition in this study is available elsewhere.[24]

For each child the following variables were derived: total physical activity (average counts per minute (cpm)) over the period worn, mean daily steps and minutes of sedentary time (defined as <100 cpm) and MVPA (>2241 cpm). These cut-off values were established to identify sedentary behaviour and light, moderate and vigorous physical activity using linear discriminant analysis and evaluated using receiver operating characteristic curve analysis, and were taken from a calibration study in seven year olds, carried out specifically for this larger study.[25] They are similar to those reported by Evenson et al.[26] The start and end dates were detected based on a specified amount of daily waking time (defined as between 07:00 and 21:59) required to be different from zero counts: the threshold for the observational period was set to at least 150 minutes per day. Total registered time is not constant across days. As a

For peer review only - http://bmjopen.bmj.com/site/about/guidelines.xhtml

result, the total amount of counts and wearing time for each child will depend on how long the accelerometer was worn. These measures were standardised by introducing the notion of a standard day with equal duration (i.e., 735 minutes, equal to mean wear time across all reliable days).[27] For each child time spent in MVPA was used to determine adherence to the CMO physical activity guideline (≥60 minutes daily) and was based on the standardised average minutes/day spent in MVPA, where the objective of standardisation was to standardise wearing time across days.

Explanatory variables

We examined the association of the following social and demographic factors, collected in the home-based interview at seven years: child's gender; age; ethnicity;[28] maternal current or most recent occupation by child age 7;[29] family structure (lone parent and number of children); and country of residence (England, Scotland, Wales, Northern Ireland) and (within England) Government Office Region of residence**.** Yearly seasons of measurement were defined astronomically, i.e. spring (21st March –20th June), summer (21st June – 20th September), autumn (21st September – 20th December), winter (21st December – 20th March).

Statistical analyses

Analyses were conducted using STATA/SE 11.2 (Stata Corporation, TX) and the R software environment for statistical computing version 2.15.0.[30;31] Sampling weights were used to account for the stratified clustered design of the MCS. Weights were adjusted for attrition between contacts at successive MCS sweeps and for missing accelerometer data. Details on the adjustment for non-response and non-compliance are given elsewhere.[27] Prevalences of the children meeting the CMO current physical activity guideline were derived, as were medians and interquartile ranges for derived activity variables (given their non-normal distributions). Quantile regression models for medians and quartiles were fitted using the R package `quantreg`[32] to

For peer review only - http://bmjopen.bmj.com/site/about/guidelines.xhtml

examine differences between socio-demographic groups in terms of time spent in physical (in)activity adjusting for season of measurement. These models do not make assumptions about the shape of the distribution. Therefore they are robust to departures from normality, as for example skewness, heavy-tailedness and heteroscedasticity, which affected all PA outcomes in this study. The Benjamini-Hochberg[33] correction for false discovery rate in multiple comparisons was also applied setting the point-wise threshold for the correction at 0.05. We used the R package `multtest`[34] to adjust *p*-values for multiple comparisons using this correction. Multivariable logistic regression models were fitted to analyse differences between socio-demographic groups for prevalences of children meeting the MVPA guideline. Prevalences were adjusted for gender, ethnicity, maternal current or most recent occupation, lone parenthood status, number of children in the household, country of residence, and season of measurement. Moran's *I* test[35] was used to assess spatial autocorrelation among regions within England using a weighted matrix with a second-order nearest neighbourhood structure.[36]

RESULTS

The majority of children in the sample (85%) were white (eTable1), 49% were boys, 88% were living in households including at least one other child and 65% lived in England. The current or most recent occupation of mothers by child age 7 years was semi-routine and routine in 36%; 22% of mothers were lone parents. Mean age when wearing the accelerometer was 7.5 years.

Seasonally-adjusted medians of derived physical activity variables are summarised in Table 1.

Median physical activity was 5954 (interquartile range (IQR): 507, 696) cpm. Median daily minutes spent in MVPA was 60. (IQR: 47.1, 76.2) and fifty per cent of children

For peer review only - http://bmjopen.bmj.com/site/about/guidelines.xhtml

were sedentary for 6.4 hours (IQR: 5.9, 6.9) or more each day. Children took a median of 10229 (IQR: 8777, 11775) daily steps. We estimated that half of all children (50.8%) achieved ≥60 minutes of daily MVPA.

Girls engaged less in total physical activity and MVPA, and took fewer steps than boys. They were also more sedentary and were less likely than boys to meet the daily MVPA recommendation (38% versus 63%).

Overall activity levels were lowest in children of Indian origin, who also recorded the least MVPA, were among the most sedentary and took the fewest steps. MVPA levels were lower in girls than in boys across all ethnic groups (Figure 2). Bangladeshi children were the least likely (32.8%) to meet the MVPA recommendation. In pairwise comparisons children of Indian ethnicity were significantly less active overall than all other ethnic groups. Those children whose mothers have never worked or had been long-term unemployed were the most likely to meet recommended levels of daily physical activity and had the lowest median of daily hours of sedentary behaviour.

Children from couple parent families engaged in less total physical activity and daily MVPA than those whose mothers did not live with partners. They also took the least steps and were least likely to meet the MVPA recommendation. Little variation was found in physical activity levels according to the number of children living in the household, although lone children in the household were slightly more sedentary.

There were significant differences between UK countries: those living in Northern Ireland engaged in the least total physical activity, daily MVPA and took the fewest steps. Children in Scotland were the least sedentary, Children in Northern Ireland had the lowest prevalence of the recommended amount of daily MVPA (43.4%), whilst 50.9% of those living in England, 51.7% of those in Wales, and 52.5% of those in Scotland, achieved the recommended amount of daily MVPA. Though spatial autocorrelation was not statistically significant (I = 0.05, p = 0.822) some regional differences within England were also identified (Figure 3); children in the North West

For peer review only - http://bmjopen.bmj.com/site/about/guidelines.xhtml

For peer review only

were the most likely (57.8%) and those in the Midlands the least likely (46%) to meet the CMO guideline.

For peer review only

For peer review only - http://bmjopen.bmj.com/site/about/guidelines.xhtml

Table 1: Description of physical activity (PA) measures; adjusted[(1)] median and interquartile range, unless otherwise stated (*n* = 6497)

	n	Overall activity (counts/min)	Sedentary (hours/day)	Moderate and vigorous (min/day)	Steps / day	% of children meeting recommended PA (standard error [(2)]
All children	6497	595<br>507, 697	6.4<br>5.9, 6.9	60.1<br>47.1, 76.2	10229<br>8777, 11775	50.8<br>(0.009)
	**Child's gender**					
Male	3176	630[a] [(3)]<br>541, 757	6.4[a]<br>5.9, 6.9	67.1[a]<br>53.9, 83.7	10739[a]<br>9213, 12499	63.3[a]<br>(0.012)
Female	3321	561[a]<br>481, 656	6.5[a]<br>6.0, 7.2	54.4[a]<br>44.4, 67.1	9699[a]<br>8389, 11063	37.8[a]<br>(0.011)
	**Child's ethnicity** [(4)]					
White	5710	597[a]<br>512, 701	6.5[a,b]<br>6.0, 7.1	60.2[a]<br>47.8, 75.9	10343[a,b,c,d,e,f]<br>8926, 11940	51.4<br>(0.010)
Mixed	168	572[b]<br>505, 643	6.8[a]<br>6.3, 7.3	62.2[b]<br>49.9, 66.7	10071[a,g,h,i]<br>8553, 11167	51.5<br>(0.040)

For peer review only - http://bmjopen.bmj.com/site/about/guidelines.xhtml

Indian	139	511[a,b,,c,d,e]<br>437, 680	6.9[b,c,d,e,f]<br>6.4, 7.4	52.6[a,b,c,d]<br>40.9, 66.7	8699[b,g]<br>7477, 9667	40.0<br>(0.049)
Pakistani	177	563[c]<br>484, 680	6.4[c]<br>5.9, 7.0	58.2<br>46.4, 74.8	9419[c,h]<br>7906, 11120	45.2<br>(0.058)
Bangladeshi	70	538<br>450, 639	6.5[d]<br>6.0, 7.0	52.9<br>42.1, 70.6	8894[d,i]<br>6889, 10013	32.8<br>(0.087)
Black	142	598[d]<br>517, 737	6.5[e]<br>6.0, 7.0	63.1[c]<br>52.6, 86.3	9468[d]<br>7878, 10857	52.0<br>(0.054)
Other	90	581[e]<br>476, 650	6.5[f]<br>6.1, 7.7	58.2<br>45.8, 74.1	9814[f]<br>8270, 10798	53.9<br>(0.081)
	**Maternal current or most recent occupation by child age 7**					
Managerial & professional occupations	2190	585<br>502, 690	6.6[a]<br>6.0, 7.1	59.4<br>46.8, 74.6	10135<br>8685, 11662	48.8[a]<br>(0.013)
Intermediate occupations	1202	597<br>511, 697	6.5[b]<br>6.1, 7.0	58.39.2<br>47.8, 75.8	10239<br>8843, 11722	48.7[b]<br>(0.017)

For peer review only - http://bmjopen.bmj.com/site/about/guidelines.xhtml

Small employers & own account workers	442	596<br>516, 671	6.6<br>6.1, 7.0	60.5[c]<br>48.3, 73.6	10237<br>8901, 11598	48.6[c]<br>(0.031)
Lower supervisory & technical occupations	288	598<br>505, 675	6.6[c]<br>6.1, 7.0	60.5<br>48.3, 73.6	10152<br>8596, 11710440	51.0<br>(0.033)
Semi-routine & routine occupations	1865	597<br>508, 710	6.5[d]<br>5.9, 7.0	61.5<br>47.3, 77.2	10380<br>8891, 12127	52.3<br>(0.015)
Never worked & long-term unemployed	268	603<br>529,, 607	6.3[a,b,c,d]<br>5.9, 6.9	62.2<br>49.1, 77.0	10116<br>8702, 11017	59.7[a,b,c]<br>(0.040)
	**Lone parenthood status**					
No	5536	589[a]<br>504, 693	6.54[a]<br>6.0, 7.1	59.6 [a]<br>47.1, 75.0	10127[a]<br>8683, 11703	49.7[a]<br>(0.010)
Yes	961	606[a]<br>521, 712	6.48[a]<br>5.9, 7.0	63.0[a]<br>50.3, 78.2	10471[a]<br>9026, 12242	54.7[a]<br>(0.021)
	**No. of children in the household**					

For peer review only - http://bmjopen.bmj.com/site/about/guidelines.xhtml

Only child	726	594<br>510, 679	6.6[a]<br>6.1, 7.1	61.9<br>48.0, 74.9	10259<br>8920, 11950	51.9<br>(0.023)
At least one other child	5771	593<br>507, 697	6.5[a]<br>6.0, 7.0	60.0<br>47.5, 75.9	10222<br>8761, 11762	50.7<br>(0.009)
	**Country**					
England	4204	589[a,b]<br>504, 692	6.6[a,b]<br>6.0, 7.1	60.6[a]<br>46.9, 75.3	10147[a,b]<br>8743, 11763	50.9[a]<br>(0.011)
Wales	898	606[a]<br>509, 711	6.5[a]<br>5.9, 7.0	61.6[b]<br>47.0, 77.8	10357[a,c]<br>8931, 11922	51.7[b]<br>(0.021)
Scotland	761	615[b,c]<br>578, 715	6.4[b,c]<br>5.9, 6.9	61.8[c]<br>50.5, 78.1	10521[b,d]<br>9022, 12027	52.5[c]<br>(0.024)
Northern Ireland	634	586[c]<br>486, 617	6.6[c]<br>6.0, 7.1	57.6[a,b,c]<br>45.5, 73.4	9895[c,d]<br>8031, 11351	43.4[a,b,c]<br>(0.021)

(1) Medians and IQR adjusted by seasons of measurement
(2) The prevalences of children meeting the recommended MVPA guidelines are adjusted for gender, ethnicity, maternal current or most recent occupation, lone parenthood status, number of children in the household, country of residence, and season of measurement.
(3) This variable was based on a derived variable from the 2001 Census ethnicity categories indicating the ethnic identity of the children in the MCS data. Aggregate groupings were imposed on these categories to create a smaller manageable number of categories. The names of the groups were also adjusted for reporting. The final ethnic groups were: white (including white-British, -Irish, -Welsh, -Scottish and other white background); Mixed; Indian; Pakistani; Bangladeshi; Black

For peer review only - http://bmjopen.bmj.com/site/about/guidelines.xhtml

(including Black-Caribbean, Black-African and Black- British); and other (including other Asian, other Black, Chinese and other).

(4) As there are many multiple comparisons among levels of factors included in the models, this table does not show any *p*-values and only indicates those pairwise differences which were significantly different after applying the Benjamini-Hochberg correction for false discovery rate. We used superindices (a-i) to indicate pairs of groups within a factor that differed significantly pairwise ($p<0.05$). For example medians of minutes of MVPA significantly differed pairwise between England and Wales (a), England and Scotland (b), and Scotland and Northern Ireland (c).

For peer review only

For peer review only - http://bmjopen.bmj.com/site/about/guidelines.xhtml

DISCUSSION

Statement of principal findings

This is the first population-based UK-wide study of objectively measured physical activity and sedentary time in primary-school aged children. We found that when measured in 2008 - 2009 51% of children met the CMO recommendation of 60 minutes or more of daily MVPA.[10,37] More than half of all children spent a substantial part of the day being sedentary.

This study highlights social and demographic variation in UK children's physical activity and inactivity. Girls were more sedentary than boys and less active across all intensities of physical activity. Ethnic differences were observed with children of Indian origin generally the least active across all outcomes and Bangladeshi children were least likely to achieve recommended levels of physical activity. There were no clear socioeconomic gradients in physical activity levels and adherence to guidelines, although there was a tendency for children whose mothers were never employed or unemployed to be slightly more active than children whose mothers were employed. Children of lone parents were slightly more active and more likely to meet the MVPA recommendation than those from couple parent families. There were differences between UK countries with children in Northern Ireland less active and least likely to achieve recommended activity levels than those from other countries. Nevertheless, with the exception of gender, the group differences observed in this study were relatively small and the most striking finding is the low level of activity across all groups.

Strengths and limitations

The strengths of this study include the large UK-wide and representative sample, the use of accelerometers to provide objective measures of physical activity and sedentary time, and the data processing undertaken to exclude any data that might be unreliable. As there is limited consensus on data reduction strategies,[38] most commonly relating to the best threshold with which to define MVPA, we derived the thresholds used in our study from a calibration study we conducted specifically in this age group and consider the thresholds used to be reliable.[25] The ability to link accelerometer data to a rich range of social and demographic information has

For peer review only - http://bmjopen.bmj.com/site/about/guidelines.xhtml

For peer review only

enabled examination, for the first time, of ethnic, social and geographical variations in adherence to policy recommendations. Acknowledged limitations include potential underestimation, of physical activity as a result of the accelerometers used, the position in which they are worn, or their removal during some activities, such as aquatic activities or contact sports.

Comparison with literature

Findings from large (defined as ≥400 participants) UK-based accelerometry studies reporting adherence to physical activity guidelines are summarised in Table 2.

Table 2: Summary of accelerometry studies in UK-based school-aged children.

Reference and study name / size	Setting	Year measured	Mean age (Years)	Threshold used to define MVPA	Prevalence sufficiently active* (%)	
					Boys	Girls
National Probability Samples						
MCS (current study) (*n*=6497)	England, Scotland, Wales and Northern Ireland	2008-09	7.5	2240 cpm[25]	63	38
HSE 2008[39] (*n*=1286)	England	2008	9.9	2802 cpm[39]	33	21
Regional Samples						

For peer review only - http://bmjopen.bmj.com/site/about/guidelines.xhtml

Basterfield et al[40]<br><br>Gateshead Millennium Study<br><br>(*n*=405)	England (Gateshead)	2006-07 and 2008-09	7.4 and 9.3	3200 cpm[19]	7yrs: 10.1<br>9yrs: 8.6	7yrs: 2.9<br>9yrs: 2.9
Owen et al[41]<br><br>CHASE<br><br>(*n*=2071)	England (London, Birmingham and Leicester)	2006-07	9.9	2000 cpm[18]	76	53
Steele et al[42]<br><br>SPEEDY<br><br>(*n*=1862)	England (Norfolk)	2007	10.2	2000 cpm[18]	81.5	59.4
Gidlow et al[43]	England (Stoke-on-Trent)	2006-07	10.4	Age-specific thresholds[44] and 3200 cpm[19]	94[44]<br>5.2[19]	89[44]<br>1.4[19]

For peer review only - http://bmjopen.bmj.com/site/about/guidelines.xhtml

CHAMPS UK (*n*=503)						
Riddoch et al[45] ALSPAC (*n*=5595)	England (Bristol)	2003-05	11	3600 cpm[46]	5.1	0.4

Table abbreviations: MCS: Millennium Cohort Study; ALSPAC: Avon Longitudinal Study of Parents and Children; SPEEDY: Sport, Physical Activity and Eating Behaviour, Environmental Determinants in Young People; CHASE: Child Heart and Health Study; CHAMPS: Children's Health and Activity Monitoring Programme in Schools; cpm, counts per minute**.** Table footnote: *Accumulating ≥60 min/day MVPA. The exact measure used to define adherence to this guideline varies across the studies tabulated: in MCS, Gateshead, SPEEDY and ALSPAC studies a mean of 60 minutes or more was used; in the CHASE study only the first day of measurement, and in the Health Survey only a subsample with seven days of observation each with at least 60 minutes of MVPA per day."

In none were children measured in population-based samples from all four UK countries. The Millennium Gateshead Study measured children of a similar age to those in our study, although in a much smaller sample. The results show wide variation in the proportions reported to adhere to recommended guidelines on activity but are consistent with our finding that boys are more active than girls. These differences may in part be due to the use of different MVPA intensity thresholds, ranging from 2000 to 3600 cpm. We examined this by recalculating our

For peer review only - http://bmjopen.bmj.com/site/about/guidelines.xhtml

data using different thresholds: the percentage of children meeting recommendations increased to 80.4% (standard error of proportion: 0.010) and 59.4% (0.011) for boys and girls, respectively, when applying a threshold of 2000 cpm and fell to 13.7% (0.008) and 0.4% (0.005) when applying a higher threshold of 3000 cpm. Clearly, therefore, our estimates are similar to some of these other studies[41;42] when a similar threshold of 2000 cpm is applied. However, other differences may reflect variation in regions, sampling methods and settings for recruitment as well as in approaches to adjustment for missing data. Further work is needed to clearly define the best thresholds for different intensities of activity in children incorporating novel statistical techniques.[47] Clarity over thresholds will be important for routine surveillance measures to monitor the achievement of policy objectives relating to physical activity. Differences in estimates of sedentary time and numbers of steps between studies may reflect measurement period, while decisions made about removal of zero values may also influence sedentary time. These parameters are not consistently reported in published reports.

Only one other large-scale study in the UK has examined ethnic differences in objectively measured physical activity. Owen et [al41] reported lower levels in South Asian children than European whites and black African-Caribbeans, with 54%, 70% and 69% respectively meeting the recommendations. We also found that children of Bangladeshi origin were the least likely to meet these recommendations. Further research is however needed to explore the different cultural and social factors that determine these ethnic differences in activity levels.

Although activity levels differed between children from different socioeconomic circumstances, with those less advantaged slightly more active, we did not find a strong social gradient; this is consistent with other published evidence.[45;48] This is the only study to report country and regional differences across the UK and so direct comparisons with other studies cannot be made.

The children in our study engaged in slightly less sedentary time than observed in older children in other UK-based accelerometry studies. For example, in the Avon Longitudinal Study of Parents and Children (ALSPAC) 7.0 and 7.3 hours of daily sedentary time were reported in

For peer review only - http://bmjopen.bmj.com/site/about/guidelines.xhtml

12-year old boys and girls respectively, increasing at older ages.[49] In a cross sectional study of English ten year olds, Steele et al[42] reported 7.5 and 7.7 hours for boys and girls, respectively.

Tudor-Locke et al[50] recommend that school-aged boys and girls should be encouraged to undertake 13,000-15,000 and 11,000-12,000 steps/day, respectively. The children in this study accumulated fewer steps than this (boys: 10732; girls: 9675); however, our estimates are very similar to those reported by Owen et al[41] (boys: 10570; girls: 9123) who also reported higher step numbers in white compared to black and Asian children, which is consistent with our findings.

Unanswered questions and future research

We are currently examining influences on physical activity and sedentary time in this representative cohort of school-aged children in more detail using longitudinal data to examine their prior determinants. Once follow up data become available from subsequent contacts with cohort members we propose to examine the consequences for health in later childhood and adolescence. Objective measures of physical activity and sedentary time can allow these influences to be characterised with greater precision and to be compared with subjective and proxy reports of engagement in physical activity and sedentary behaviours, also collected within this cohort. We have documented the accelerometer data and derived variables, and ~~are making~~ these accessible to other researchers through the UK Data Archive.

Implications for practice and policy

Last year the London 2012 Olympic Games promised to inspire a generation to take part in sport. The UK sporting success in August 2012 provided a platform for the Government's plans for an Olympic and Paralympic sporting legacy,[51] to encourage and enable pupils to engage in competitive sports and activities. The results of our study provide a useful baseline and strongly suggest that contemporary UK children are insufficiently active, implying that effort is needed to boost physical activity among young people to the level appropriate for good health. This is likely to require population-wide interventions across the range of physical activity domains.

For peer review only - http://bmjopen.bmj.com/site/about/guidelines.xhtml

For peer review only

There are many opportunities to increase physical activity through sport but also in other areas.[52] The journey to school has been recognised as an important opportunity for increasing total as well as more intense physical activity. Urban environments are also important for children's physical activity,[53] and factors from available green space to perceptions of safety all impact on children's activity. The full potential to boost physical activity will only be realised with a comprehensive policy response that increases time spent in more intense physical activity and decreases the time spent being sedentary. Investing in this area is a vital component to deliver the Olympic legacy and improve the short and long-term health of our children.

Declaration of interests: None

Authors Contributions

CR, MG, MCB, LJG, CD, FS and TP contributed to data processing. TP, MCB and FS conducted the data analysis and, with LJG and CD, interpreted the data and drafted the article. All authors contributed to the study conception/design, critically revised the article and reviewed the final draft of the article. LJG is guarantor.

Acknowledgements

The Centre for Paediatric Epidemiology and Biostatistics is supported in part by the Medical Research Council in its capacity as the MRC Centre of Epidemiology for Child Health (Grant reference G0400546). Research at the UCL Institute of Child Health and Great Ormond Street Hospital for Children receives a proportion of the funding from the Department of Health's National Institute for Health Research Biomedical Research Centres funding scheme. The fourth sweep of the Millennium Cohort Study was funded by grants to Professor Health Joshi, former director of the study, from the Economic and Social Research Council and a consortium of government funders. The current director is Professor Lucinda Platt. The co-operation of the participating families is gratefully acknowledged. Accelerometer data collection was funded by

For peer review only - http://bmjopen.bmj.com/site/about/guidelines.xhtml

the Wellcome Trust (principal investigator Professor Carol Dezateux, grant 084686/Z/08/A). The study sponsors played no part in the design, data analysis and interpretation of this study, the writing of the manuscript, or the decision to submit the paper for publication and the authors' work was independent of their funders.

Data sharing statement:

The MCS data for surveys 1 to 4 are currently available via the Economic and Social Data Service; the MCS accelerometer data will also shortly be made available.

For peer review only

For peer review only - http://bmjopen.bmj.com/site/about/guidelines.xhtml

FIGURES

Figure 1: Flow diagram of study participants

Figure 2: Median minutes per day of MVPA by gender and ethnicity adjusting by season of measurement

Figure 3: Prevalence of children meeting physical activity recommendation of ≥60 min/day MVPA (%)

For peer review only

For peer review only - http://bmjopen.bmj.com/site/about/guidelines.xhtml

Reference List

(1) Hills AP, Andersen LB, Byrne NM. Physical activity and obesity in children. *Br J Sports Med* 2011; 45(11):866-870.

(2) Owen CG, Nightingale CM, Rudnicka AR, Sattar N, Cook DG, Ekelund U et al. Physical activity, obesity and cardiometabolic risk factors in 9- to 10-year-old UK children of white European, South Asian and black African-Caribbean origin: the Child Heart And health Study in England (CHASE). *Diabetologia* 2010; 53(8):1620-1630.

(3) Tobias JH, Steer CD, Mattocks CG, Riddoch C, Ness AR. Habitual levels of physical activity influence bone mass in 11-year-old children from the United Kingdom: findings from a large population-based cohort. *J Bone Miner Res* 2007; 22(1):101-109.

(4) Ekeland E, Heian F, Hagen KB. Can exercise improve self esteem in children and young people? A systematic review of randomised controlled trials. *Br J Sports Med* 2005; 39(11):792-798.

(5) Ekelund U, Brage S, Froberg K, Harro M, Anderssen SA, Sardinha LB et al. TV viewing and physical activity are independently associated with metabolic risk in children: the European Youth Heart Study. *PLoS Med* 2006; 3(12):e488.

(6) Kwon S, Janz KF. Tracking of accelerometry-measured physical activity during childhood: ICAD pooled analysis. *Int J Behav Nutr Phys Act* 2012; 9(1):68.

(7) Biddle SJ, Pearson N, Ross GM, Braithwaite R. Tracking of sedentary behaviours of young people: a systematic review. *Prev Med* 2010; 51(5):345-351.

(8) U.S.Department of Health and Human Services. 2008 Physical Activity Guidelines for Americans. 2008. Washington, U.S. Department of Health and Human Services.

(9) Tremblay MS, Warburton DER, Janssen I, Paterson DH, Latimer AE, Rhodes RE et al. New Canadian Physical Activity Guidelines. *Appl Physiol Nutr Metab* 2011; 36(1):36-46.

(10) Department of Health. Start active, stay active: a report on physical activity from the four home countries' Chief Medical Officers. 11-7-0011.

(11) Harrell JS, McMurray RG, Baggett CD, Pennell ML, Pearce PF, Bangdiwala SI. Energy costs of physical activities in children and adolescents. *Med Sci Sports Exerc* 2005; 37(2):329-336.

(12) Reilly JJ, Ness AR, Sherriff A. Epidemiological and physiological approaches to understanding the etiology of pediatric obesity: finding the needle in the haystack. *Pediatr Res* 2007; 61(6):646-652.

(13) Troiano RP. Large-scale applications of accelerometers: new frontiers and new questions. *Med Sci Sports Exerc* 2007; 39(9):1501.

(14) Trost SG, McIver KL, Pate RR. Conducting accelerometer-based activity assessments in field-based research. *Med Sci Sports Exerc* 2005; 37(11 Suppl):S531-S543.

(15) Hamer M, Fisher A. Are interventions to promote physical activity in children a waste of time? *BMJ* 2012; 345:e6320.

For peer review only - http://bmjopen.bmj.com/site/about/guidelines.xhtml

(16) Hansen K. Millennium Cohort Study First, Second, Third and Fourth Surveys: A Guide to the Datasets (Sixth Edition). 2012. London, Centre for Longitudinal Studies, University of London.

(17) Ott AE, Pate RR, Trost SG, Ward DS, Saunders R. The use of uniaxial and triaxial accelerometers to measure children's "free-play" physical activity. *Pediatric Exercise Science* 2000; 12(4):360-370.

(18) Trost SG, Ward DS, Moorehead SM, Watson PD, Riner W, Burke JR. Validity of the computer science and applications (CSA) activity monitor in children. *Med Sci Sports Exerc* 1998; 30(4):629-633.

(19) Puyau MR, Adolph AL, Vohra FA, Butte NF. Validation and calibration of physical activity monitors in children. *Obes Res* 2002; 10(3):150-157.

(20) Ekelund U, Yngve A, Brage S, Westerterp K, Sjostrom M. Body movement and physical activity energy expenditure in children and adolescents: how to adjust for differences in body size and age. *Am J Clin Nutr* 2004; 79(5):851-856.

(21) Geraci M. pawacc: Physical activity with accelerometers. R package version 1.0. http://r-forge r-project org/projects/pawacc/ [2012

(22) Geraci M, Rich C, Sera F, Cortina-Borja M, Griffiths LJ, Dezateux C. (2012) Technical report on accelerometry data processing in the Millennium Cohort Study. http://discovery ucl ac uk/1361699/

(23) Rich C, Geraci M, Griffiths LJ, Sera F, Dezateux C, Cortina-Borja M. Quality control methods in accelerometer data processing: Defining minimum wear time. PLoS One In Press.

(24) Rich C, Cortina-Borja M, Dezateux C, Geraci M, Sera F, Calderwood L et al. Predictors of non-response in a UK-wide cohort study of children's accelerometer-determined physical activity using postal methods. *BMJ Open* 2013; 3(3).

(25) Pulsford RM, Cortina-Borja M, Rich C, Kinnafick FE, Dezateux C, Griffiths LJ. Actigraph accelerometer-defined boundaries for sedentary behaviour and physical activity intensities in 7 year old children. *PLoS One* 2011; 6(8):e21822.

(26) Evenson KR, Catellier DJ, Gill K, Ondrak KS, McMurray RG. Calibration of two objective measures of physical activity for children. *J Sports Sci* 2008; 26(14):1557-1565.

(27) Griffiths LJ, Rich C, Geraci M, Sera F, Cortina-Borja M, Pouliou T et al. (2013) Technical report on the enhancement of Millennium Cohort Study data with accelerometer-derived measures of physical activity and sedentary behaviour in seven year olds. http://www esds ac uk/doc/7238/mrdoc/pdf/mcs4_pa_technical_report pdf

(28) Office for National Statistics. Ethnic Group Statistics: A Guide for the Collection and Classification of Ethnicity Data. 2003. London, The Stationery Office.

(29) Rose D, Pevalin D. A Researcher's Guide to the National Statistics Socio-economic Classification. 2003. London, Sage Publications.

(30) R Core Team. R: A language and environment for statistical computing. Vienna, Austria.: R Foundation for Statistical Computing; 2012.

For peer review only - http://bmjopen.bmj.com/site/about/guidelines.xhtml

(31) Geraci M. Estimation of regression quantiles in complex surveys with data missing at random: An application to birthweight determinants. Statistical Methods in Medical Research In press. 2013.

(32) Koenker R. Quanteg: Quantile Regression R package version 4.79. http://CRAN R-project org/package=quanteg [2012 [cited 2012 Aug. 8];

(33) Benjamini Y, Hochberg Y. Controlling the False Discovery Rate: A Practical and Powerful Approach to Multiple Testing. *Journal of the Royal Statistical Society Series B (Methodological)* 1995; 57(1):289-300.

(34) Pollard KS, Gilbert HN, Ge Y, Taylor S, Dudoit S. Multtest: Resampling-based multiple hypothesis testing. 2012. R package version 2.12.0.

(35) Cliff AD, Ord JK. Spatial processes. London: Pion; 1981.

(36) Anselin L. Exploratory spatial data analysis and geographic information systems. In: Longley P, Brooks S, Macmillan B, McDonnell R, editors. GeoComputation: A primer. New York: Willey; 1994. 77-94.

(37) Department of Health. At least 5 a week: physical activity and health outcomes: a review of the Chief Medical Officer. 2004. London, Department of Health.

(38) Ekelund U, Tomkinson G, Armstrong N. What proportion of youth are physically active? Measurement issues, levels and recent time trends. *Br J Sports Med* 2011; 45(11):859-865.

(39) The NHS Information Centre. The Health Survey for England – 2008: Physical Activity and Fitness. 2009.

(40) Basterfield L, Adamson AJ, Frary JK, Parkinson KN, Pearce MS, Reilly JJ. Longitudinal study of physical activity and sedentary behavior in children. *Pediatrics* 2011; 127(1):e24-e30.

(41) Owen CG, Nightingale CM, Rudnicka AR, Cook DG, Ekelund U, Whincup PH. Ethnic and gender differences in physical activity levels among 9-10-year-old children of white European, South Asian and African-Caribbean origin: the Child Heart Health Study in England (CHASE Study). *Int J Epidemiol* 2009; 38(4):1082-1093.

(42) Steele RM, van Sluijs EM, Cassidy A, Griffin SJ, Ekelund U. Targeting sedentary time or moderate- and vigorous-intensity activity: independent relations with adiposity in a population-based sample of 10-y-old British children. *Am J Clin Nutr* 2009; 90(5):1185-1192.

(43) Gidlow CJ, Cochrane T, Davey R, Smith H. In-school and out-of-school physical activity in primary and secondary school children. *J Sports Sci* 2008; 26(13):1411-1419.

(44) Trost SG, Pate RR, Sallis JF, Freedson PS, Taylor WC, Dowda M et al. Age and gender differences in objectively measured physical activity in youth. *Med Sci Sports Exerc* 2002; 34(2):350-355.

(45) Riddoch CJ, Mattocks C, Deere K, Saunders J, Kirkby J, Tilling K et al. Objective measurement of levels and patterns of physical activity. *Arch Dis Child* 2007; 92(11):963-969.

For peer review only - http://bmjopen.bmj.com/site/about/guidelines.xhtml

(46) Mattocks C, Leary S, Ness A, Deere K, Saunders J, Tilling K et al. Calibration of an accelerometer during free-living activities in children. *Int J Pediatr Obes* 2007; 2(4):218-226.

(47) Crouter SE, Clowers KG, Bassett DR, Jr. A novel method for using accelerometer data to predict energy expenditure. *J Appl Physiol* 2006; 100(4):1324-1331.

(48) Ruiz JR, Ortega FB, Martinez-Gomez D, Labayen I, Moreno LA, De B, I et al. Objectively measured physical activity and sedentary time in European adolescents: the HELENA study. *Am J Epidemiol* 2011; 174(2):173-184.

(49) Mitchell JA, Pate RR, Dowda M, Mattocks C, Riddoch C, Ness AR et al. A Prospective Study of Sedentary Behavior in a Large Cohort of Youth. *Med Sci Sports Exerc* 2011.

(50) Tudor-Locke C, Craig CL, Beets MW, Belton S, Cardon GM, Duncan S et al. How many steps/day are enough? for children and adolescents. *Int J Behav Nutr Phys Act* 2011; 8:78.

(51) Department of Culture MaS. Beyond 2012 – The London 2012 Legacy Story. 2012. London, Department of Culture, Media and Sport.

(52) NICE. Promoting physical activity, active play and sport for pre-school and school-age children and young people in family, pre-school, school and community settings. 2009. London, NICE.

(53) Wheeler BW, Cooper AR, Page AS, Jago R. Greenspace and children's physical activity: a GPS/GIS analysis of the PEACH project. *Prev Med* 2010; 51(2):148-152.

For peer review only

How active are our children? Findings from the Millennium Cohort Study

Lucy J Griffiths (senior research fellow)[1], Mario Cortina-Borja (senior lecturer)[1], Francesco Sera (senior research associate)[1], Theodora Pouliou (research associate)[1], Marco Geraci (lecturer)[1], Carly Rich (research associate)[1], Tim J Cole (professor)[1], Catherine Law (professor)[1], Heather Joshi (professor)[2], Andrew R Ness (professor)[3], Susan A Jebb (head of diet and population health)[4] and Carol Dezateux (professor)[1]

[1] MRC Centre of Epidemiology for Child Health, UCL Institute of Child Health, 30 Guilford Street, London, WC1N 1EH.

[2] Quantitative Social Science, Institute of Education, University of London, 20 Bedford Way, London, WC1H 0AL.

[3] School of Oral and Dental Sciences, Bristol Dental School, Lower Maudlin Street, Bristol BS1 2LY.

[4] MRC Human Nutrition Research, Elsie Widdowson Laboratory, 120 Fulbourn Road, Cambridge, CB1 9NL.

Corresponding author: Lucy Griffiths; address as above; lucy.griffiths@ucl.ac.uk

Study design: Prospective cohort study

Keywords: physical activity, sedentary, accelerometry, children, cohort study

For peer review only

ABSTRACT

Objective To describe levels of physical activity, sedentary time and adherence to Chief Medical Officers (CMO) physical activity guidelines among primary school-aged children across the UK using objective accelerometer-based measurements.

Design Nationally representative prospective cohort study.

Setting Children born across the UK, between 2000 and 2002.

Participants 6497 7-8 year old singleton children for whom reliable accelerometer data were available for at least 10 hours a day for at least two days

Main outcome measures Physical activity in counts per minute (cpm); time spent in sedentary and moderate to vigorous intensity physical activity (MVPA); proportion of children meeting CMO guidelines (≥60 min/day MVPA); average daily steps.

Explanatory measures: Gender, ethnicity, maternal **current / most recent occupation** ~~socioeconomic circumstances~~, lone parenthood status, number of children in the household, and country / region of residence.

Results The median daily physical activity level was 595 cpm (interquartile range (IQR): **507** ~~504~~, 697). Children spent a median of **60** ~~61~~ minutes (IQR: 47-76) in MVPA per day and were sedentary for a median of six and a half hours per day (IQR: 6-7). Only 51% met CMO guidelines, with girls (38%) less active than boys (63%). Children took an average of **10229** ~~10980~~ (95% CI **(8777,11775)** ~~(8712, 11897)~~) steps each day. **Children of Indian ethnicity were significantly less active overall than all other ethnic groups.** Children of ~~Indian~~ **Bangladeshi** origin and those living in Northern Ireland were least likely to meet CMO guidelines.

Conclusions Only half of seven year old children in the UK achieve recommended levels of physical activity, with significant gender, ~~social, demographic~~ **ethnic** and geographic variations. **Longitudinal studies are needed to better understand the relevance of these (in)activity patterns for long term health and well-being.** ~~Population wide efforts are needed to boost physical activity among young people to the level appropriate for good health. This is likely to require a broad range of policy~~

For peer review only - http://bmjopen.bmj.com/site/about/guidelines.xhtml

interventions. In the meantime population-wide efforts to boost physical activity among young people are needed which are likely to require a broad range of policy interventions.

For peer review only

For peer review only - http://bmjopen.bmj.com/site/about/guidelines.xhtml

ARTICLE SUMMARY

Article focus

- This paper describes levels of physical activity, sedentary time and adherence to Chief Medical Officers (CMO) physical activity guidelines among primary school-aged children across the UK using objective accelerometer-based measurements.

Key messages

- 51% of seven year old UK children achieve current recommendations for daily physical activity; this is significantly lower in girls (38%) than in boys (63%). This is also lowest in children living in Northern Ireland.
- Half of all UK seven year olds are sedentary for six and a half hours or more each day.
- Social and demographic variations in physical (in) activity levels are otherwise smaller.
- A comprehensive policy response is needed to boost physical activity and decrease sedentary time among all young children to the levels appropriate for good health.

Strengths and limitations of this study

- This is the first UK-wide study of children's objectively measured physical (in) activity.
- Linkage of the accelerometer data to a range of social and demographic information enabled examination of ethnic, social and geographical variations in activity levels and adherence to policy recommendations.
- While accelerometers provide higher levels of measurement precision relative to self-report methods of physical activity, they can underestimate **certain** activities ~~that do not include vertical movement of the trunk (e.g. cycling) and aquatic activities (e.g.~~ **like** swimming.

For peer review only - http://bmjopen.bmj.com/site/about/guidelines.xhtml

- **The prevalence of adherence to current recommendations for physical activity is sensitive to the thresholds used to define moderate and vigorous physical activity.**

For peer review only

For peer review only - http://bmjopen.bmj.com/site/about/guidelines.xhtml

INTRODUCTION

The physical and psychological benefits of physical activity for children and adolescents include reduced adiposity[1] and cardiometabolic risk factors,[2] improvements in musculoskeletal health[3] and psychological well-being.[4] Conversely, and independently of physical activity levels, high levels of sedentary behaviours such as TV viewing may also predict a poor cardiometabolic risk profile.[5] As both physical activity and sedentary behaviour track over time,[6;7] children with low levels of activity may be at risk of continuing at this level in later life.

Guidelines for physical activity in children have recently been revised in the United States,[8] Canada[9] and the United Kingdom (UK).[10] The latter were launched in July 2011 as a UK-wide consensus from the four nations' Chief Medical Officers (CMO) on the amount and type of physical activity for good health at each stage of the lifecourse.[10] It is now recommended that all young people engage in moderate to vigorous intensity physical activity (MVPA) (Box) for at least 60 minutes and up to several hours every day. A greater emphasis is now placed on vigorous intensity activities and, for the first time, it is recommended that extended sedentary time should be reduced (although a daily limit is not specified).

Box Examples of sedentary, moderate and vigorous intensity physical activities

**Activity Level**	**Age-adjusted Metabolic equivalent (MET)* ~~Definition~~**	**Examples[10]**
**Sedentary**	**1.0 - 1.5**	**Lying, sitting, watching TV, playing video games**

For peer review only

For peer review only - http://bmjopen.bmj.com/site/about/guidelines.xhtml

**Moderate**	**3.0 – 5.9 METs**	**Bike riding and playground activities. These activities will cause children to get warmer and breathe harder and their hearts to beat faster, but they should still be able to carry on a conversation**
**Vigorous**	**6.0 - 8.9 METs**	**Fast running and swimming. These activities will cause children to get warmer, breathe much harder and their hearts to beat rapidly, making it more difficult to carry on a conversation.**

***MET: metabolic equivalent; 1 MET is defined as the energy expenditure of sitting quietly, equivalent in children to a resting oxygen intake of ~6 $mL.kg^{-1}.min^{-1}$ [11]**

~~Box Examples of sedentary, moderate and vigorous intensity physical activities~~

~~Activity Level~~	~~Definition~~	~~Examples~~[10]
~~Sedentary~~	~~1.0 MET*s~~	~~Lying, sitting, watching TV, playing video games~~
~~Moderate~~	~~3.0 – 5.9 METs~~	~~Bike riding and playground activities. These activities will cause children to get warmer and breathe harder and their hearts to beat faster, but they should still be able to carry on a conversation~~
~~Vigorous~~	~~6.0 - 8.9 METs~~	~~Fast running and swimming. These activities will cause children to get warmer, breathe much harder and their hearts to beat rapidly, making it more difficult to carry on a conversation.~~

~~*MET: metabolic equivalent; 1 MET of energy is used by the body at rest.~~

For peer review only

For peer review only - http://bmjopen.bmj.com/site/about/guidelines.xhtml

The development of accelerometer-based activity monitors has enabled the frequency, intensity and duration of free-living activity to be measured objectively in large scale population studies,[12-14] documenting the proportion of children meeting recommended guidelines.[15] To date however, accelerometers have not been used in a representative sample of children from all four UK countries.

We report on the time spent in physical activity and sedentary behaviour in a UK-wide representative sample of primary school-aged children participating in a cohort study. We also evaluate adherence to CMO guidelines and variation according to a range of socio-demographic characteristics.

METHODS

The Millennium Cohort Study (MCS) is a prospective study of the social, economic and health-related circumstances of children born in the UK between September 2000 and January 2002.[16] The original cohort comprised 18,818 children (72% of those approached) whose parents were first interviewed at home when their child was aged nine months. Since then data have been collected when the children were aged three, five and seven years, with follow-up currently being conducted at 11 years and planned for 14 years and beyond. This study received research ethics approval from the Northern and Yorkshire Research Ethics Committee (07/MRE03/32).

Physical activity data

Physical activity and sedentary time were measured using the Actigraph GT1M accelerometer (Actigraph, Pensacola, Florida), which has been demonstrated to measure physical activity reliably in children when compared to measures derived

For peer review only - http://bmjopen.bmj.com/site/about/guidelines.xhtml

from heart rate monitoring,[17] indirect and room calorimetry [18;19] and doubly labelled water.[20]

A total of 14,043 children (13,681 singletons) were interviewed at age seven years and invited to participate in the accelerometry study. Those who consented were subsequently sent an accelerometer in the post, programmed to use a 15 second sampling epoch and to record activity as counts and steps. Participants were instructed to start wearing their accelerometer, on an elastic belt round the waist, the morning after they received it and to continue doing so during waking hours for seven consecutive days. They were asked to remove the accelerometer when bathing or during other aquatic activities.

Data were collected between May 2008 and August 2009. Accelerometers were returned by 9772 singleton children. Data were downloaded using Actigraph software version 3.8.3 (Actigraph, Pensacola, Florida) and subsequently processed in-house,[21] according to pre-determined processing criteria.[22] Non-wear time, defined as time periods of consecutive zero activity counts of 20 minutes or more, was excluded from analysis. Extreme count values above a threshold of ≥11,715 cpm were removed. **A study was performed to discriminate between days when the accelerometer was worn and days when it was likely not worn (e.g., during postal delivery) on the basis of the minimum number of valid minutes per day. The threshold was set to ≥ 150 minutes per day. The first day with registered time exceeding the threshold was set as start date. The last day with registered time above the threshold was set as end date.[22]** Participants with registered time of ≥10 hours on at least ≥2 days were included in analyses; only days with ≥10 hours of recorded time were included **as this has been shown to produce reliable estimates of PA.[23]** This resulted in a final sample size of 6497 singleton children

For peer review only - http://bmjopen.bmj.com/site/about/guidelines.xhtml

(3176 boys) (Figure 1) with a total of 36,309 days (nearly 449000 hours) of observation.

Small differences were found in the demographic characteristics of the sample of children with reliable accelerometer data relative to the whole cohort sample (*n*=13681) interviewed at age seven years (eTable1). **In particular, boys, children living in Wales, those belonging to the Indian, Pakistan/Bangladeshi, and Black Caribbean/African ethnic groups, as well as children belonging to families with a single respondent at the interview showed a lower probability of having a valid accelerometer file.** Information on predictors of reliable data acquisition in this study is available elsewhere.[24]

For each child the following variables were derived: total physical activity (average counts per minute (cpm)) over the period worn, mean daily steps and minutes of sedentary time (defined as <100 cpm) and MVPA (>2241 cpm). **These cut-off values were established to identify sedentary behaviour and light, moderate and vigorous physical activity using linear discriminant analysis and evaluated using receiver operating characteristic curve analysis, and were taken from a calibration study in seven year olds, carried out specifically for this larger study.**[25] **They are similar to those reported by Evenson et al.**[26] ~~**These cut-offs were chosen to optimise the discrimination between activity levels and were taken from a calibration study in seven year olds, carried out specifically for this larger study**~~ ~~For the mean daily steps and minutes of sedentary time and MVPA, which were related to the wear time period of the accelerometer, the summary measures for each child were calculated as a weighted mean of the daily values, with weight calculated as the powered inverse of the ratio between observed wear time and standard wear time. Standard wear time was fixed at 735 minutes, which was the mean wear time across all reliable days. The power values were~~

For peer review only - http://bmjopen.bmj.com/site/about/guidelines.xhtml

chosen to minimize the correlation between summary variables and daily wear time. **The start and end dates were detected based on a specified amount of daily waking time (defined as between 07:00 and 21:59) required to be different from zero counts: the threshold for the observational period was set to at least 150 minutes per day. Total registered time is not constant across days. As a result, the total amount of counts and wearing time for each child will depend on how long the accelerometer was worn. These measures were standardised by introducing the notion of a standard day with equal duration (i.e., 735 minutes, equal to mean wear time across all reliable days).[27] For each child time spent in MVPA was used to determine adherence to the CMO physical activity guideline (≥60 minutes daily)** and was **based on the standardised average minutes/day spent in MVPA, where the objective of standardisation was to standardise wearing time across days.**

Explanatory variables

We examined the association of the following social and demographic factors, collected in the home-based interview at seven years: child's gender; age; ethnicity;[28] maternal **current or most recent occupation by child age 7** socioeconomic status;[29] family structure (lone parent and number of children); and country of residence (England, Scotland, Wales, Northern Ireland) and (within England) Government Office Region of residence**. Yearly seasons of measurement were defined astronomically, i.e. spring (21st March –20th June), summer (21st June – 20th September), autumn (21st September – 20th December), winter (21st December – 20th March).**

Statistical analyses

Analyses were conducted using STATA/SE 11.2 (Stata Corporation, TX) and the R software environment for statistical computing version 2.15.0.[30;31] Sampling weights

For peer review only - http://bmjopen.bmj.com/site/about/guidelines.xhtml

were used to account for the stratified clustered design of the MCS. Weights were adjusted for attrition between contacts at successive MCS sweeps and for missing accelerometer data. **Details on the adjustment for non-response and non-compliance are given elsewhere.[27] Prevalences of the children meeting the CMO current physical activity guideline** ~~Percentages~~ **were derived, as were medians and interquartile ranges for derived activity variables (given their non-normal distributions). Quantile regression models for medians and quartiles were fitted using the R package `quantreg`[32] to examine differences between socio-demographic groups in terms of time spent in physical (in)activity adjusting for season of measurement.** ~~**These nonparametric models provide a flexible framework to analyse skewed distributions.**~~ **These models do not make assumptions about the shape of the distribution. Therefore they are robust to departures from normality, as for example skewness, heavy-tailedness and heteroscedasticity, which affected all PA outcomes in this study.** The Benjamini-Hochberg[33] correction for false discovery rate in multiple comparisons was also applied setting the point-wise threshold for the correction at 0.05. We used the R package `multtest`[34] to adjust *p*-values for multiple comparisons using this correction. Multivariable logistic regression models were fitted to analyse differences between socio-demographic groups for prevalences of children meeting the MVPA guideline. **Prevalences were adjusted for gender, ethnicity, maternal current or most recent occupation, lone parenthood status, number of children in the household, country of residence, and season of measurement.** Moran's *I* test[35] was used to assess spatial autocorrelation among regions within England using a weighted matrix with a second-order nearest neighbourhood structure.[36]

RESULTS

For peer review only - http://bmjopen.bmj.com/site/about/guidelines.xhtml

The majority of children in the sample (85%) were white (eTable1), 49% were boys, 88% were living in households including at least one other child and 65% lived in England. The **current or most recent occupation of** mothers **by child age 7 years was** of 35% of children were in semi-routine and routine **in 36%;** occupations, and 22% **of mothers** were lone parents. Mean age when wearing the accelerometer was 7.5 years.

Seasonally-adjusted medians of derived physical activity variables are summarised in Table 1.

Median physical activity was 5954 (interquartile range (IQR): 507, 696) cpm. Median daily minutes spent in MVPA was 60. (IQR: 47.1, 76.2) and fifty per cent of children were sedentary for 6.4 hours (IQR: 5.9, 6.9) or more each day. Children took a median of 10229 (IQR: 8777, 11775) daily steps. We estimated that half of all children (50.8%) achieved ≥60 minutes of daily MVPA.

Girls engaged less in total physical activity and MVPA, and took fewer steps than boys. They were also more sedentary and were less likely than boys to meet the daily MVPA recommendation (38% versus 63%).

Overall activity levels were lowest in children of Indian origin, who also recorded the least MVPA, were among the most sedentary and took the fewest steps. MVPA levels were lower in girls than in boys across all ethnic groups (Figure 2). **Bangladeshi children were the least likely (32.8%) to meet the MVPA recommendation.** Black children were the most likely (57%), and children of Indian origin the least likely (36%), to meet the MVPA recommendation; the difference between these two groups was significant in unadjusted (*p*=0.013) but not in adjusted analyses (*p*=0.176). **In pairwise comparisons children of Indian ethnicity were significantly less active overall than all other ethnic groups.** Children with mothers in lower supervisory and technical occupations had the lowest overall physical activity levels and took the least steps. Children whose mothers were small employers and own account workers recorded the least daily MVPA and were least

For peer review only - http://bmjopen.bmj.com/site/about/guidelines.xhtml

~~likely to meet the MVPA recommendation.~~ Those children whose mothers have never worked or had been long-term unemployed were the most likely to meet recommended levels of daily physical activity and had the lowest median of daily hours of sedentary behaviour.

Children from couple parent families engaged in less total physical activity and daily MVPA than those whose mothers did not live with partners. They also took the least steps and were least likely to meet the MVPA recommendation. ~~Both groups engaged in similar amounts of sedentary time.~~

Little variation was found in physical activity levels according to the number of children living in the household, although lone children in the household were slightly more ~~**active**~~ sedentary.

~~Significant differences between UK countries were found: those living in Northern Ireland engaged in the least total physical activity, daily MVPA and took the fewest steps. Children in Northern Ireland and Scotland were more sedentary than those in England and Wales. 52% of children in Wales and England, and 42% in Northern Ireland, achieved the recommended amount of daily MVPA. Though spatial autocorrelation was not statistically significant~~ ~~**(*I* = 0.05, *p* = 0.822)**~~ ~~**(I = 0.09, p = 0.249)**~~ ~~some interesting regional differences within England were also identified (Figure 3); children in the North West were the most active (60% met the recommended level) and those in the Midlands the least active (47%).~~

There were significant differences between UK countries: those living in Northern Ireland engaged in the least total physical activity, daily MVPA and took the fewest steps. Children in Scotland were the least sedentary, Children in Northern Ireland had the lowest prevalence of the recommended amount of daily MVPA (43.4%), whilst 50.9% of those living in England, 51.7% of those in Wales, and 52.5% of those in Scotland, achieved the recommended amount of daily MVPA. Though spatial autocorrelation was not statistically significant (*I* = 0.05, *p* = 0.822) ~~**(I = 0.09, p = 0.249)**~~ **some regional differences within England**

For peer review only

were also identified (Figure 3); children in the North West were the most likely (57.8%) and those in the Midlands the least likely (46%) to meet the CMO guideline.

For peer review only

For peer review only - http://bmjopen.bmj.com/site/about/guidelines.xhtml

Table 1: Description of physical activity (PA) measures; weighted median and interquartile range, unless otherwise stated (*n* = 6497)

	Overall activity (counts/min)	Sedentary (hours/day)	Moderate and vigorous (min/day)	Steps / day	% of children meeting recommended PA (standard error of proportions)
All children	594<br>504, 697	6.5<br>6.0, 7.1	60.6<br>47.2, 76.2	10180<br>8712, 11897	50.9<br>(0.009)
**Child's gender**					
Male	629[a]<br>537, 736	6.4[a]<br>5.8, 6.9	67.4[a]<br>53.4, 84.0	10732[a]<br>9125, 12495	63.2[a]<br>(0.012)
Female	556[a]<br>477, 657	6.7[a]<br>6.1, 7.2	54.0[a]<br>42.7, 67.2	9675[a]<br>8283, 11127	38.1[a]<br>(0.011)
**Child's ethnicity**					
White	597[a,b]<br>506, 702	6.5[a,b]<br>6.0, 7.1	60.6[a]<br>47.4, 76.4	10294[a,b,c,d,e]<br>8828, 12051	51.0[a]<br>(0.009)

For peer review only - http://bmjopen.bmj.com/site/about/guidelines.xhtml

Mixed	571[a,c]<br>507, 671	6.8[a,c]<br>6.3, 7.4	62.8[b]<br>51.3, 70.7	9895[f]<br>8553, 11167	54.5<br>(0.040)
Indian	527[b,c,d,e,f]<br>448, 632	6.8[b]<br>6.4, 7.3	52.2[a,b,c,d]<br>40.3, 66.9	8728[a,f]<br>7554, 9991	36.4[a]<br>(0.050)
Pakistani	604[d]<br>501, 693	6.4[c]<br>5.8, 7.0	60.7[c]<br>46.8, 75.2	9441[b]<br>7895, 11212	53.2<br>(0.051)
Bangladeshi	546<br>423, 671	6.5<br>5.8, 7.2	53.2<br>40.1, 74.6	8825[c]<br>6709, 10331	40.7<br>(0.068)
Black	596[e]<br>515, 711	6.5<br>5.9, 7.0	65.7[d]<br>49.4, 86.1	9114[d]<br>8112, 10784	56.6<br>(0.057)
Other	588[f]<br>480, 655	6.5<br>5.9, 7.4	61.4<br>46.1, 74.8	9582[e]<br>8486, 11310	50.9<br>(0.068)
**Maternal socioeconomic circumstances**					
Managerial & professional occupations	587[a]<br>498, 689	6.6<br>6.0, 7.1	59.2[a]<br>46.9, 75.9	10101<br>8655, 11667	48.9[a,b]<br>(0.013)

For peer review only

Intermediate occupations	591[b]<br>509, 700	6.6<br>6.0, 7.1	59.2[b]<br>46.7, 75.9	10151<br>8818, 11771	48.8[c,d]<br>(0.018)
Small employers & own account workers	585[e]<br>499, 672	6.6<br>6.0, 7.1	58.5[c]<br>47.6, 69.9	10124<br>8701, 11561	48.0[e]<br>(0.030)
Lower supervisory & technical occupations	573[c,d]<br>504, 682	6.6<br>6.1, 7.1	59.8<br>48.6, 71.4	9845[a]<br>8598, 11440	49.9<br>(0.036)
Semi-routine & routine occupations	608[a,b,c,e]<br>506, 711	6.5<br>5.9, 7.0	61.8[a,b,c]<br>47.1, 77.9	10371[a]<br>8752, 12181	53.1[a,c]<br>(0.015)
Never worked & long-term unemployed	601[d]<br>512, 688	6.4<br>5.8, 6.9	62.8<br>47.9, 76.6	10112<br>8593, 12148	56.3[b,d,e]<br>(0.042)
**Lone parenthood status**					
No	589[a]<br>500, 693	6.5<br>6.0, 7.1	59.7[a]<br>46.6, 75.6	10097[a]<br>8655, 11795	49.3[a]<br>(0.009)
Yes	607[a]<br>520, 710	6.5<br>5.9, 7.0	63.4[a]<br>49.6, 78.8	10418[a]<br>8946, 12280	56.6[a]<br>(0.020)
**No. of children in the household**					

For peer review only - http://bmjopen.bmj.com/site/about/guidelines.xhtml

Only child	596<br>499, 691	6.6[a]<br>6.1, 7.2	61.6<br>46.3, 74.8	10155<br>8775, 11962	52.1<br>(0.025)
At least one other child	594<br>505, 699	6.5[a]<br>6.0, 7.1	60.5<br>47.3, 76.3	10182<br>8699, 11892	50.7<br>(0.009)
**Country**					
England	597[a,b]<br>506, 700	6.5[a,b,c]<br>6.0, 7.1	61.0[a,b]<br>47.5, 76.4	10206[a,b]<br>8746, 11949	51.8[a,b]<br>(0.010)
Wales	597[c,d]<br>507, 711	6.4[a,d,e]<br>5.9, 6.9	61.4[c,d]<br>46.4, 77.9	10369[c,d]<br>8791, 12122	52.0[c,d]<br>(0.020)
Scotland	575[a,c]<br>494, 680	6.6[b,d]<br>6.1, 7.1	57.3[a,c]<br>45.8, 73.2	9911[a,c]<br>8563, 11285	44.1[a,c]<br>(0.021)
Northern Ireland	558[b,d]<br>475, 662	6.6[c,e]<br>6.1, 7.1	55.9[b,d]<br>43.1, 72.1	9545[b,d]<br>8048, 11057	41.2[b,d]<br>(0.020)

Table footnote: As there are many multiple comparisons among levels of factors included in the models, this table does not show any *p*-values and only indicates those pairwise differences which were significantly different after applying the Benjamini-Hochberg correction for false discovery rate in multiple comparisons. We have used super indices (a-e) to indicate pairs of groups within a factor that differed significantly (p<0.05). Percentage of children meeting recommended PA column: differences are adjusted for gender, ethnicity, maternal socioeconomic circumstances, lone parenthood status, no. of children in the household, and country.

For peer review only - http://bmjopen.bmj.com/site/about/guidelines.xhtml

Table 1: Description of physical activity (PA) measures; adjusted[(1)] median and interquartile range, unless otherwise stated (*n* = 6497)

	n	Overall activity (counts/min)	Sedentary (hours/day)	Moderate and vigorous (min/day)	Steps / day	% of children meeting recommended PA (standard error [(2)]
All children	6497	595<br>507, 697	6.4<br>5.9, 6.9	60.1<br>47.1, 76.2	10229<br>8777, 11775	50.8<br>(0.009)
	**Child's gender**					
Male	3176	630[a (3)]<br>541, 757	6.4[a]<br>5.9, 6.9	67.1[a]<br>53.9, 83.7	10739[a]<br>9213, 12499	63.3[a]<br>(0.012)
Female	3321	561[a]<br>481, 656	6.5[a]<br>6.0, 7.2	54.4[a]<br>44.4, 67.1	9699[a]<br>8389, 11063	37.8[a]<br>(0.011)
	**Child's ethnicity [(4)]**					
White	5710	597[a]<br>512, 701	6.5[a,b]<br>6.0, 7.1	60.2[a]<br>47.8, 75.9	10343[a,b,c,d,e,f]<br>8926, 11940	51.4<br>(0.010)
Mixed	168	572[b]<br>505, 643	6.8[a]<br>6.3, 7.3	62.2[b]<br>49.9, 66.7	10071[a,g,h,i]<br>8553, 11167	51.5<br>(0.040)

For peer review only - http://bmjopen.bmj.com/site/about/guidelines.xhtml

Indian	139	511[a,b,,c,d,e]<br>437, 680	6.9[b,c,d,e,f]<br>6.4, 7.4	52.6[a,b,c,d]<br>40.9, 66.7	8699[b,g]<br>7477, 9667	40.0<br>(0.049)
Pakistani	177	563[c]<br>484, 680	6.4[c]<br>5.9, 7.0	58.2<br>46.4, 74.8	9419[c,h]<br>7906, 11120	45.2<br>(0.058)
Bangladeshi	70	538<br>450, 639	6.5[d]<br>6.0, 7.0	52.9<br>42.1, 70.6	8894[d,i]<br>6889, 10013	32.8<br>(0.087)
Black	142	598[d]<br>517, 737	6.5[e]<br>6.0, 7.0	63.1[c]<br>52.6, 86.3	9468[d]<br>7878, 10857	52.0<br>(0.054)
Other	90	581[e]<br>476, 650	6.5[f]<br>6.1, 7.7	58.2<br>45.8, 74.1	9814[f]<br>8270, 10798	53.9<br>(0.081)
	**Maternal current or most recent occupation by child age 7**					
Managerial & professional occupations	2190	585<br>502, 690	6.6[a]<br>6.0, 7.1	59.4<br>46.8, 74.6	10135<br>8685, 11662	48.8[a]<br>(0.013)
Intermediate occupations	1202	597<br>511, 697	6.5[b]<br>6.1, 7.0	58.39.2<br>47.8, 75.8	10239<br>8843, 11722	48.7[b]<br>(0.017)

For peer review only - http://bmjopen.bmj.com/site/about/guidelines.xhtml

Small employers & own account workers	442	596<br>516, 671	6.6<br>6.1, 7.0	60.5[c]<br>48.3, 73.6	10237<br>8901, 11598	48.6[c]<br>(0.031)
Lower supervisory & technical occupations	288	598<br>505, 675	6.6[c]<br>6.1, 7.0	60.5<br>48.3, 73.6	10152<br>8596, 11710440	51.0<br>(0.033)
Semi-routine & routine occupations	1865	597<br>508, 710	6.5[d]<br>5.9, 7.0	61.5<br>47.3, 77.2	10380<br>8891, 12127	52.3<br>(0.015)
Never worked & long-term unemployed	268	603<br>529,, 607	6.3[a,b,c,d]<br>5.9, 6.9	62.2<br>49.1, 77.0	10116<br>8702, 11017	59.7[a,b,c]<br>(0.040)
	**Lone parenthood status**					
No	5536	589[a]<br>504, 693	6.54[a]<br>6.0, 7.1	59.6 [a]<br>47.1, 75.0	10127[a]<br>8683, 11703	49.7[a]<br>(0.010)
Yes	961	606[a]<br>521, 712	6.48[a]<br>5.9, 7.0	63.0[a]<br>50.3, 78.2	10471[a]<br>9026, 12242	54.7[a]<br>(0.021)
	**No. of children in the household**					

For peer review only - http://bmjopen.bmj.com/site/about/guidelines.xhtml

Only child	726	594<br>510, 679	6.6[a]<br>6.1, 7.1	61.9<br>48.0, 74.9	10259<br>8920, 11950	51.9<br>(0.023)
At least one other child	5771	593<br>507, 697	6.5[a]<br>6.0, 7.0	60.0<br>47.5, 75.9	10222<br>8761, 11762	50.7<br>(0.009)
	**Country**					
England	4204	589[a,b]<br>504, 692	6.6[a,b]<br>6.0, 7.1	60.6[a]<br>46.9, 75.3	10147[a,b]<br>8743, 11763	50.9[a]<br>(0.011)
Wales	898	606[a]<br>509, 711	6.5[a]<br>5.9, 7.0	61.6[b]<br>47.0, 77.8	10357[a,c]<br>8931, 11922	51.7[b]<br>(0.021)
Scotland	761	615[b,c]<br>578, 715	6.4[b,c]<br>5.9, 6.9	61.8[c]<br>50.5, 78.1	10521[b,d]<br>9022, 12027	52.5[c]<br>(0.024)
Northern Ireland	634	586[c]<br>486, 617	6.6[c]<br>6.0, 7.1	57.6[a,b,c]<br>45.5, 73.4	9895[c,d]<br>8031, 11351	43.4[a,b,c]<br>(0.021)

(1) **Medians and IQR adjusted by seasons of measurement**
(2) **The prevalences of children meeting the recommended MVPA guidelines are adjusted for gender, ethnicity, maternal current or most recent occupation, lone parenthood status, number of children in the household, country of residence, and season of measurement.**
(3) **This variable was based on a derived variable from the 2001 Census ethnicity categories indicating the ethnic identity of the children in the MCS data. Aggregate groupings were imposed on these categories to create a smaller manageable number of categories. The names of the groups were also adjusted for reporting. The final ethnic groups were: white (including white-British, -Irish, -Welsh, -Scottish and other white background); Mixed; Indian;**

For peer review only - http://bmjopen.bmj.com/site/about/guidelines.xhtml

Pakistani; Bangladeshi; Black (including Black-Caribbean, Black-African and Black- British); and other (including other Asian, other Black, Chinese and other).

(4) As there are many multiple comparisons among levels of factors included in the models, this table does not show any *p*-values and only indicates those pairwise differences which were significantly different after applying the Benjamini-Hochberg correction for false discovery rate. We used superindices (a-i) to indicate pairs of groups within a factor that differed significantly pairwise (p<0.05). For example medians of minutes of MVPA significantly differed pairwise between England and Wales (a), England and Scotland (b), and Scotland and Northern Ireland (c).

For peer review only - http://bmjopen.bmj.com/site/about/guidelines.xhtml

DISCUSSION

Statement of principal findings

This is the first population-based UK-wide study of objectively measured physical activity and sedentary time in primary-school aged children. We found that when measured in 2008 - 2009 51% of children met the CMO recommendation of 60 minutes or more of daily MVPA.[10,37] More than half of all children spent a substantial part of the day being sedentary.

This study highlights social and demographic variation in UK children's physical activity and inactivity. Girls were more sedentary than boys and less active across all intensities of physical activity. Ethnic differences were observed with children of Indian origin generally the least active across all **outcomes and Bangladeshi children were least likely to achieve recommended levels of physical activity**. ~~and Black children were more likely than other groups to meet the MVPA recommendation.~~ There were no clear socioeconomic gradients in physical activity levels and adherence to guidelines, although ~~some it appeared that~~ **there was a tendency** for ~~less advantaged~~ children **whose mothers were never employed or unemployed** ~~were~~ **to be** slightly more active than ~~more advantaged~~ children **whose mothers were employed.** Children of lone parents were **slightly** more active and **more** likely to meet the MVPA recommendation than those from couple parent families. There were differences between UK countries ~~across all sedentary and physical activity outcomes~~ with children in Northern Ireland less active and least likely to achieve recommended activity levels than those from other countries. Nevertheless, with the exception of gender, the group differences observed in this study were relatively small and the most striking finding is the low level of activity across all groups.

Strengths and limitations

The strengths of this study include the large UK-wide and representative sample, the use of accelerometers to provide objective measures of physical activity and sedentary time, and the data processing undertaken to exclude any data that might be unreliable. As there is limited consensus on data reduction strategies,[38] most commonly relating to the best threshold with

For peer review only - http://bmjopen.bmj.com/site/about/guidelines.xhtml

which to define MVPA, we derived the thresholds used in our study from a calibration study we conducted specifically in this age group and consider the thresholds used to be reliable.[25] The ability to link accelerometer data to a rich range of social and demographic information has enabled examination, for the first time, of ethnic, social and geographical variations in adherence to policy recommendations. **Acknowledged limitations include potential underestimation, of physical activity as a result of the accelerometers used, the position in which they are worn, or their removal during some activities, such as aquatic activities or contact sports.** ~~Acknowledged limitations include underestimation, as a result of the accelerometers used, of activities that do not include vertical movement of the trunk, like cycling, and aquatic activities, such as swimming.~~

Comparison with literature

Findings from large (defined as ≥400 participants) UK-based accelerometry studies reporting adherence to physical activity guidelines are summarised in Table 2.

Table 2: Summary of accelerometry studies in UK-based school-aged children.

Reference and study name / size	Setting	Year measured	Mean age (Years)	Threshold used to define MVPA	Prevalence sufficiently active* (%)	
					Boys	Girls
National Probability Samples						
MCS (current study) (*n*=6497)	England, Scotland, Wales and Northern Ireland	2008-09	7.5	2240 cpm[25]	63	38

For peer review only

HSE 2008[39] (*n*=1286)	England	2008	9.9	2802 cpm[39]	33	21
Regional Samples						
Basterfield et al[40] Gateshead Millennium Study (*n*=405)	England (Gateshead)	2006-07 and 2008-09	7.4 and 9.3	3200 cpm[19]	7yrs: 10.1 9yrs: 8.6	7yrs: 2.9 9yrs: 2.9
Owen et al[41] CHASE (*n*=2071)	England (London, Birmingham and Leicester)	2006-07	9.9	2000 cpm[18]	76	53
Steele et al[42] SPEEDY	England (Norfolk)	2007	10.2	2000 cpm[18]	81.5	59.4

For peer review only - http://bmjopen.bmj.com/site/about/guidelines.xhtml

(*n*=1862)						
Gidlow et al[43]<br>CHAMPS UK<br>(*n*=503)	England (Stoke-on-Trent)	2006-07	10.4	Age-specific thresholds[44] and 3200 cpm[19]	94[44]<br>5.2[19]	89[44]<br>1.4[19]
~~Wheeler et al~~<br>~~PEACH~~<br>~~(*n*= 1053)~~	~~England (Bristol)~~	~~2006-09~~	~~10.9~~	~~3200 cpm[19]~~	~~6.8~~	~~5.3~~
Riddoch et al[45]<br>ALSPAC<br>(*n*=5595)	England (Bristol)	2003-05	11	3600 cpm[46]	5.1	0.4

~~Table footnote: *Accumulating ≥60 min/day MVPA.~~ Table abbreviations: MCS: Millennium Cohort Study; ALSPAC: Avon Longitudinal Study of Parents and Children; SPEEDY: Sport, Physical Activity and Eating Behaviour, Environmental Determinants in Young People; CHASE:

For peer review only

For peer review only - http://bmjopen.bmj.com/site/about/guidelines.xhtml

Child Heart and Health Study; CHAMPS: Children's Health and Activity Monitoring Programme in Schools; **PEACH project: Personal and Environmental Associations with Children's Health;** cpm, counts per minute**. Table footnote: *Accumulating ≥60 min/day MVPA. The exact measure used to define adherence to this guideline varies across the studies tabulated: in MCS, Gateshead, SPEEDY and ALSPAC studies a mean of 60 minutes or more was used; in the CHASE study only the first day of measurement, and in the Health Survey only a subsample with seven days of observation each with at least 60 minutes of MVPA per day."**

In none were children measured in population-based samples from all four UK countries. The Millennium Gateshead Study measured children of a similar age to those in our study, although in a much smaller sample. The results show wide variation in the proportions reported to adhere to recommended guidelines on activity but are consistent with our finding that boys are more active than girls. These differences may in part be due to the use of different MVPA intensity thresholds, ranging from 2000 to 3600 cpm. We examined this by recalculating our data using different thresholds: the percentage of children meeting recommendations increased to 80.4% (standard error of proportion: 0.010) and 59.4% (0.011) for boys and girls, respectively, when applying a threshold of 2000 cpm and fell to 13.7% (0.008) and 0.4% (0.005) when applying a higher threshold of 3000 cpm. Clearly, therefore, our estimates are similar to some of these other studies[41;42] when a similar threshold of 2000 cpm is applied. However, other differences may reflect variation in regions, sampling methods and settings for recruitment as well as in approaches to adjustment for missing data. Further work is needed to clearly define the best thresholds for different intensities of activity in children incorporating novel statistical techniques.[47] Clarity over thresholds will be important for routine surveillance measures to monitor the achievement of policy objectives relating to physical activity. Differences in estimates of sedentary time and numbers of steps between studies may reflect measurement period, while decisions made about removal of zero values may also influence sedentary time. These parameters are not consistently reported in published reports.

For peer review only - http://bmjopen.bmj.com/site/about/guidelines.xhtml

Only one other large-scale study in the UK has examined ethnic differences in objectively measured physical activity. Owen et al[41] reported lower levels in South Asian children than European whites and black African-Caribbeans, with 54%, 70% and 69% respectively meeting the recommendations. We also found that children of ~~Indian and~~ Bangladeshi origin were the least~~, and those of Black ethnic origin among the most,~~ likely to meet these recommendations. **Further research is however needed to explore the different cultural and social factors that determine these ethnic differences in activity levels.**

Although activity levels differed between children from different socioeconomic circumstances, with those less advantaged slightly more active, we did not find a strong social gradient; this is consistent with other published evidence.[45;48] This is the only study to report country and regional differences across the UK and so direct comparisons with other studies cannot be made.

The children in our study engaged in slightly less sedentary time than observed in older children in other UK-based accelerometry studies. For example, in the Avon Longitudinal Study of Parents and Children (ALSPAC) 7.0 and 7.3 hours of daily sedentary time were reported in 12-year old boys and girls respectively, increasing at older ages.[49] In a cross sectional study of English ten year olds, Steele et al[42] reported 7.5 and 7.7 hours for boys and girls, respectively.

Tudor-Locke et al[50] recommend that school-aged boys and girls should be encouraged to undertake 13,000-15,000 and 11,000-12,000 steps/day, respectively. The children in this study accumulated fewer steps than this (boys: 10732; girls: 9675); however, our estimates are very similar to those reported by Owen et al[41] (boys: 10570; girls: 9123) who also reported higher step numbers in white compared to black and Asian children, which is consistent with our findings.

Unanswered questions and future research

We are currently examining influences on physical activity and sedentary time in this representative cohort of school-aged children in more detail using longitudinal data to examine their prior determinants. Once follow up data become available from subsequent contacts with

For peer review only - http://bmjopen.bmj.com/site/about/guidelines.xhtml

cohort members we propose to examine the consequences for health in later childhood and adolescence. Objective measures of physical activity and sedentary time can allow these influences to be characterised with greater precision and to be compared with subjective and proxy reports of engagement in physical activity and sedentary behaviours, also collected within this cohort. We have documented the accelerometer data and derived variables, and ~~are making~~ these accessible to other researchers through the UK Data Archive.

Implications for practice and policy

Last year the London 2012 Olympic Games promised to inspire a generation to take part in sport. The UK **sporting** success in August **2012** provide**d** a platform for the Government's plans for an Olympic and Paralympic sporting legacy,[51] to encourage and enable pupils to engage in competitive sports and activities. The results of our study provide a useful baseline and **strongly suggest** that contemporary UK children are insufficiently active, implying that effort is needed to boost physical activity among young people to the level appropriate for good health. This is likely to require population-wide interventions across the range of physical activity domains. There are many opportunities to increase physical activity through sport but also in other areas.[52] The journey to school has been recognised as an important opportunity for increasing total as well as more intense physical activity. Urban environments are also important for children's physical activity,[53] and factors from available green space to perceptions of safety all impact on children's activity. The full potential to boost physical activity will only be realised with a comprehensive policy response that increases time spent in more intense physical activity and decreases the time spent being sedentary. Investing in this area is a vital component to deliver the Olympic legacy and improve the short and long-term health of our children.

Declaration of interests: None

For peer review only - http://bmjopen.bmj.com/site/about/guidelines.xhtml

Authors Contributions

CR, MG, MCB, LJG, CD, FS and TP contributed to data processing. TP, MCB and FS conducted the data analysis and, with LJG and CD, interpreted the data and drafted the article. All authors contributed to the study conception/design, critically revised the article and reviewed the final draft of the article. LJG is guarantor.

Acknowledgements

The Centre for Paediatric Epidemiology and Biostatistics is supported in part by the Medical Research Council in its capacity as the MRC Centre of Epidemiology for Child Health (Grant reference G0400546). Research at the UCL Institute of Child Health and Great Ormond Street Hospital for Children receives a proportion of the funding from the Department of Health's National Institute for Health Research Biomedical Research Centres funding scheme. The fourth sweep of the Millennium Cohort Study was funded by grants to Professor Health Joshi, former director of the study, from the Economic and Social Research Council and a consortium of government funders. The current director is Professor Lucinda Platt. The co-operation of the participating families is gratefully acknowledged. Accelerometer data collection was funded by the Wellcome Trust (principal investigator Professor Carol Dezateux, grant 084686/Z/08/A). The study sponsors played no part in the design, data analysis and interpretation of this study, the writing of the manuscript, or the decision to submit the paper for publication and the authors' work was independent of their funders.

Data sharing statement:

The MCS data for surveys 1 to 4 are currently available via the Economic and Social Data Service; the MCS accelerometer data will also shortly be made available.

For peer review only

FIGURES

Figure 1: Flow diagram of study participants

~~**Figure 2** Median minutes per day of moderate to vigorous physical activity by gender (1: boys; 2: girls) and ethnicity~~

Figure 2: Median minutes per day of MVPA by gender and ethnicity adjusting by season of measurement

Figure 3: Prevalence of children meeting physical activity recommendation of ≥60 min/day MVPA (%)

For peer review only

For peer review only - http://bmjopen.bmj.com/site/about/guidelines.xhtml

Reference List

(1) Hills AP, Andersen LB, Byrne NM. Physical activity and obesity in children. *Br J Sports Med* 2011; 45(11):866-870.

(2) Owen CG, Nightingale CM, Rudnicka AR, Sattar N, Cook DG, Ekelund U et al. Physical activity, obesity and cardiometabolic risk factors in 9- to 10-year-old UK children of white European, South Asian and black African-Caribbean origin: the Child Heart And health Study in England (CHASE). *Diabetologia* 2010; 53(8):1620-1630.

(3) Tobias JH, Steer CD, Mattocks CG, Riddoch C, Ness AR. Habitual levels of physical activity influence bone mass in 11-year-old children from the United Kingdom: findings from a large population-based cohort. *J Bone Miner Res* 2007; 22(1):101-109.

(4) Ekeland E, Heian F, Hagen KB. Can exercise improve self esteem in children and young people? A systematic review of randomised controlled trials. *Br J Sports Med* 2005; 39(11):792-798.

(5) Ekelund U, Brage S, Froberg K, Harro M, Anderssen SA, Sardinha LB et al. TV viewing and physical activity are independently associated with metabolic risk in children: the European Youth Heart Study. *PLoS Med* 2006; 3(12):e488.

(6) Kwon S, Janz KF. Tracking of accelerometry-measured physical activity during childhood: ICAD pooled analysis. *Int J Behav Nutr Phys Act* 2012; 9(1):68.

(7) Biddle SJ, Pearson N, Ross GM, Braithwaite R. Tracking of sedentary behaviours of young people: a systematic review. *Prev Med* 2010; 51(5):345-351.

(8) U.S.Department of Health and Human Services. 2008 Physical Activity Guidelines for Americans. 2008. Washington, U.S. Department of Health and Human Services.

(9) Tremblay MS, Warburton DER, Janssen I, Paterson DH, Latimer AE, Rhodes RE et al. New Canadian Physical Activity Guidelines. *Appl Physiol Nutr Metab* 2011; 36(1):36-46.

(10) Department of Health. Start active, stay active: a report on physical activity from the four home countries' Chief Medical Officers. 11-7-0011.

(11) Harrell JS, McMurray RG, Baggett CD, Pennell ML, Pearce PF, Bangdiwala SI. Energy costs of physical activities in children and adolescents. *Med Sci Sports Exerc* 2005; 37(2):329-336.

(12) Reilly JJ, Ness AR, Sherriff A. Epidemiological and physiological approaches to understanding the etiology of pediatric obesity: finding the needle in the haystack. *Pediatr Res* 2007; 61(6):646-652.

(13) Troiano RP. Large-scale applications of accelerometers: new frontiers and new questions. *Med Sci Sports Exerc* 2007; 39(9):1501.

(14) Trost SG, McIver KL, Pate RR. Conducting accelerometer-based activity assessments in field-based research. *Med Sci Sports Exerc* 2005; 37(11 Suppl):S531-S543.

(15) Hamer M, Fisher A. Are interventions to promote physical activity in children a waste of time? *BMJ* 2012; 345:e6320.

For peer review only - http://bmjopen.bmj.com/site/about/guidelines.xhtml

(16) Hansen K. Millennium Cohort Study First, Second, Third and Fourth Surveys: A Guide to the Datasets (Sixth Edition). 2012. London, Centre for Longitudinal Studies, University of London.

(17) Ott AE, Pate RR, Trost SG, Ward DS, Saunders R. The use of uniaxial and triaxial accelerometers to measure children's "free-play" physical activity. *Pediatric Exercise Science* 2000; 12(4):360-370.

(18) Trost SG, Ward DS, Moorehead SM, Watson PD, Riner W, Burke JR. Validity of the computer science and applications (CSA) activity monitor in children. *Med Sci Sports Exerc* 1998; 30(4):629-633.

(19) Puyau MR, Adolph AL, Vohra FA, Butte NF. Validation and calibration of physical activity monitors in children. *Obes Res* 2002; 10(3):150-157.

(20) Ekelund U, Yngve A, Brage S, Westerterp K, Sjostrom M. Body movement and physical activity energy expenditure in children and adolescents: how to adjust for differences in body size and age. *Am J Clin Nutr* 2004; 79(5):851-856.

(21) Geraci M. pawacc: Physical activity with accelerometers. R package version 1.0. http://r-forge r-project org/projects/pawacc/ [2012

(22) Geraci M, Rich C, Sera F, Cortina-Borja M, Griffiths LJ, Dezateux C. (2012) Technical report on accelerometry data processing in the Millennium Cohort Study. http://discovery ucl ac uk/1361699/

(23) Rich C, Geraci M, Griffiths LJ, Sera F, Dezateux C, Cortina-Borja M. Quality control methods in accelerometer data processing: Defining minimum wear time. PLoS One In Press.

(24) Rich C, Cortina-Borja M, Dezateux C, Geraci M, Sera F, Calderwood L et al. Predictors of non-response in a UK-wide cohort study of children's accelerometer-determined physical activity using postal methods. *BMJ Open* 2013; 3(3).

(25) Pulsford RM, Cortina-Borja M, Rich C, Kinnafick FE, Dezateux C, Griffiths LJ. Actigraph accelerometer-defined boundaries for sedentary behaviour and physical activity intensities in 7 year old children. *PLoS One* 2011; 6(8):e21822.

(26) Evenson KR, Catellier DJ, Gill K, Ondrak KS, McMurray RG. Calibration of two objective measures of physical activity for children. *J Sports Sci* 2008; 26(14):1557-1565.

(27) Griffiths LJ, Rich C, Geraci M, Sera F, Cortina-Borja M, Pouliou T et al. (2013) Technical report on the enhancement of Millennium Cohort Study data with accelerometer-derived measures of physical activity and sedentary behaviour in seven year olds. http://www esds ac uk/doc/7238/mrdoc/pdf/mcs4_pa_technical_report pdf

(28) Office for National Statistics. Ethnic Group Statistics: A Guide for the Collection and Classification of Ethnicity Data. 2003. London, The Stationery Office.

(29) Rose D, Pevalin D. A Researcher's Guide to the National Statistics Socio-economic Classification. 2003. London, Sage Publications.

(30) R Core Team. R: A language and environment for statistical computing. Vienna, Austria.: R Foundation for Statistical Computing; 2012.

For peer review only - http://bmjopen.bmj.com/site/about/guidelines.xhtml

(31) Geraci M. Estimation of regression quantiles in complex surveys with data missing at random: An application to birthweight determinants. Statistical Methods in Medical Research In press. 2013.

(32) Koenker R. Quanteg: Quantile Regression R package version 4.79. http://CRAN R-project org/package=quanteg [2012 [cited 2012 Aug. 8];

(33) Benjamini Y, Hochberg Y. Controlling the False Discovery Rate: A Practical and Powerful Approach to Multiple Testing. *Journal of the Royal Statistical Society Series B (Methodological)* 1995; 57(1):289-300.

(34) Pollard KS, Gilbert HN, Ge Y, Taylor S, Dudoit S. Multtest: Resampling-based multiple hypothesis testing. 2012. R package version 2.12.0.

(35) Cliff AD, Ord JK. Spatial processes. London: Pion; 1981.

(36) Anselin L. Exploratory spatial data analysis and geographic information systems. In: Longley P, Brooks S, Macmillan B, McDonnell R, editors. GeoComputation: A primer. New York: Willey; 1994. 77-94.

(37) Department of Health. At least 5 a week: physical activity and health outcomes: a review of the Chief Medical Officer. 2004. London, Department of Health.

(38) Ekelund U, Tomkinson G, Armstrong N. What proportion of youth are physically active? Measurement issues, levels and recent time trends. *Br J Sports Med* 2011; 45(11):859-865.

(39) The NHS Information Centre. The Health Survey for England – 2008: Physical Activity and Fitness. 2009.

(40) Basterfield L, Adamson AJ, Frary JK, Parkinson KN, Pearce MS, Reilly JJ. Longitudinal study of physical activity and sedentary behavior in children. *Pediatrics* 2011; 127(1):e24-e30.

(41) Owen CG, Nightingale CM, Rudnicka AR, Cook DG, Ekelund U, Whincup PH. Ethnic and gender differences in physical activity levels among 9-10-year-old children of white European, South Asian and African-Caribbean origin: the Child Heart Health Study in England (CHASE Study). *Int J Epidemiol* 2009; 38(4):1082-1093.

(42) Steele RM, van Sluijs EM, Cassidy A, Griffin SJ, Ekelund U. Targeting sedentary time or moderate- and vigorous-intensity activity: independent relations with adiposity in a population-based sample of 10-y-old British children. *Am J Clin Nutr* 2009; 90(5):1185-1192.

(43) Gidlow CJ, Cochrane T, Davey R, Smith H. In-school and out-of-school physical activity in primary and secondary school children. *J Sports Sci* 2008; 26(13):1411-1419.

(44) Trost SG, Pate RR, Sallis JF, Freedson PS, Taylor WC, Dowda M et al. Age and gender differences in objectively measured physical activity in youth. *Med Sci Sports Exerc* 2002; 34(2):350-355.

(45) Riddoch CJ, Mattocks C, Deere K, Saunders J, Kirkby J, Tilling K et al. Objective measurement of levels and patterns of physical activity. *Arch Dis Child* 2007; 92(11):963-969.

For peer review only - http://bmjopen.bmj.com/site/about/guidelines.xhtml

(46) Mattocks C, Leary S, Ness A, Deere K, Saunders J, Tilling K et al. Calibration of an accelerometer during free-living activities in children. *Int J Pediatr Obes* 2007; 2(4):218-226.

(47) Crouter SE, Clowers KG, Bassett DR, Jr. A novel method for using accelerometer data to predict energy expenditure. *J Appl Physiol* 2006; 100(4):1324-1331.

(48) Ruiz JR, Ortega FB, Martinez-Gomez D, Labayen I, Moreno LA, De B, I et al. Objectively measured physical activity and sedentary time in European adolescents: the HELENA study. *Am J Epidemiol* 2011; 174(2):173-184.

(49) Mitchell JA, Pate RR, Dowda M, Mattocks C, Riddoch C, Ness AR et al. A Prospective Study of Sedentary Behavior in a Large Cohort of Youth. *Med Sci Sports Exerc* 2011.

(50) Tudor-Locke C, Craig CL, Beets MW, Belton S, Cardon GM, Duncan S et al. How many steps/day are enough? for children and adolescents. *Int J Behav Nutr Phys Act* 2011; 8:78.

(51) Department of Culture MaS. Beyond 2012 – The London 2012 Legacy Story. 2012. London, Department of Culture, Media and Sport.

(52) NICE. Promoting physical activity, active play and sport for pre-school and school-age children and young people in family, pre-school, school and community settings. 2009. London, NICE.

(53) Wheeler BW, Cooper AR, Page AS, Jago R. Greenspace and children's physical activity: a GPS/GIS analysis of the PEACH project. *Prev Med* 2010; 51(2):148-152.

For peer review only

For peer review only - http://bmjopen.bmj.com/site/about/guidelines.xhtml

Figure 1 Flow diagram of study participants

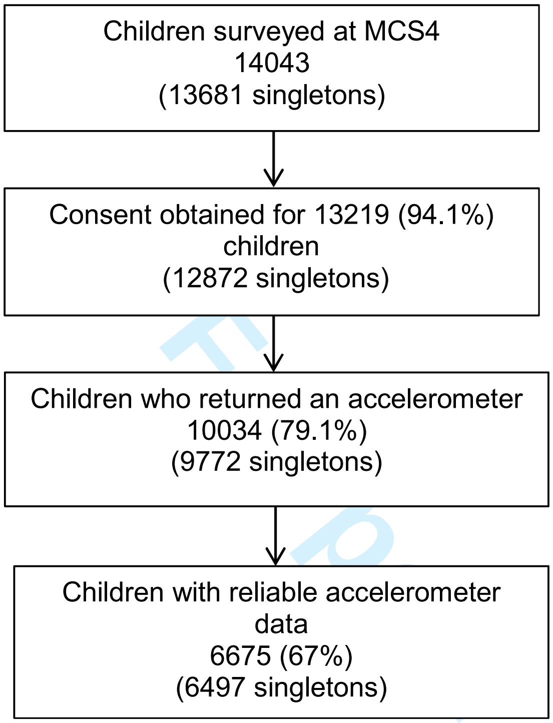

For peer review only

eTable1 Description of sample; percentages, unless otherwise stated

	All singleton children in MCS4 N (weighted %)			Singleton children with reliable accelerometer data (≥2 days for at least ≥10 hours per day) N (weighted %)		
	**Male n=6950**	**Female n=6731**	**Total n=13681**	**Male n=3176**	**Female n=3321**	**Total n=6497**
Child's ethnic group						
White	5828 (85.6)	5610 (85.3)	11438 (85.4)	2795 (85.0)	2915 (85.3)	5710 (85.2)
Mixed	178 (3.1)	194 (3.3)	372 (3.3)	85 (3.5)	83 (3.0)	168 (3.2)
Indian	179 (2.1)	162 (1.8)	341 (1.9)	61 (1.8)	78 (2.2)	139 (2.0)
Pakistani	308 (3.4)	321 (3.7)	629 (3.5)	93 (4.2)	84 (3.4)	177 (3.8)
Bangladeshi	113 (1.1)	141 (1.3)	254 (1.2)	30 (1.2)	40 (1.4)	70 (1.3)
Black	221 (3.2)	193 (2.8)	414 (3.0)	71 (2.9)	71 (2.8)	142 (2.8)
Other ethnic group	121 (1.6)	106 (1.7)	227 (1.6)	41 (1.4)	49 (1.9)	90 (1.6)
Maternal socioeconomic circumstances						
Managerial and professional occupations	1942 (28.1)	1906 (29.6)	3848 (28.8)	1098 (28.2)	1092 (27.8)	2190 (28.0)

For peer review

Intermediate occupations	1229 (18.7)	1115 (17.9)	2344 (18.3)	592 (18.6)	610 (17.9)	1202 (18.3)
Small employers and own account workers	422 (7.0)	403 (6.5)	825 (6.7)	217 (7.0)	225 (6.7)	442 (6.8)
Lower supervisory and technical occupations	293 (4.5)	296 (4.6)	589 (4.6)	138 (5.0)	150 (5.5)	288 (5.2)
Semi-routine and routine occupations	2263 (35.8)	2197 (34.8)	4460 (35.3)	893 (34.7)	972 (35.2)	1865 (34.9)
Never worked and long-term unemployed	426 (6.0)	466 (6.6)	892 (6.3)	118 (6.5)	150 (7.0)	268 (6.7)
Lone parenthood						
No	5530 (77.9)	5375 (78.9)	10905 (78.4)	2718 (78.3)	2818 (77.7)	5536 (78.0)
Yes	1420 (22.1)	1356 (21.1)	2776 (21.6)	458 (21.7)	503 (22.3)	961 (22.0)
No. children in the household						
Only child	903 (13.2)	868 (13.4)	1771 (13.3)	340 (11.7)	386 (13.0)	726 (12.4)
At least one other child	6047 (86.8)	5863 (86.6)	11910 (86.7)	2836 (88.3)	2935 (87.0)	5771 (87.6)
Country of residence						
England	4451 (63.9)	4335 (64.6)	8786 (64.2)	2064 (64.7)	2140 (65.2)	4204 (65.0)

For peer review only

Wales	1015 (14.8)	930 (13.6)	1945 (14.2)	450 (14.8)	448 (13.3)	898 (14.0)
Scotland	809 (11.3)	790 (12.0)	1599 (11.6)	363 (11.1)	398 (11.9)	761 (11.5)
Northern Ireland	675 (10.0)	676 (9.8)	1351 (9.9)	299 (9.4)	335 (9.6)	634 (9.5)

Missing data: Child's ethnic group, 6 for all children in MCS4 and 1 for children with activity data; Maternal socioeconomic class, 723 for all children in MCS4 and 259 for children with activity data.

For peer review only

Figure 2

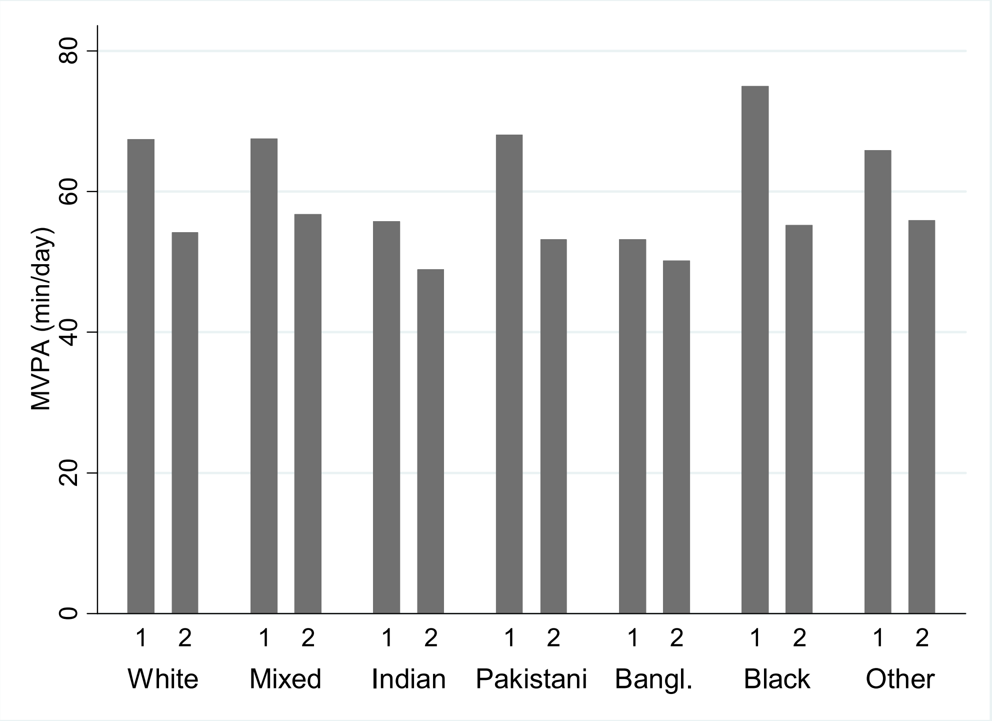

For peer review only

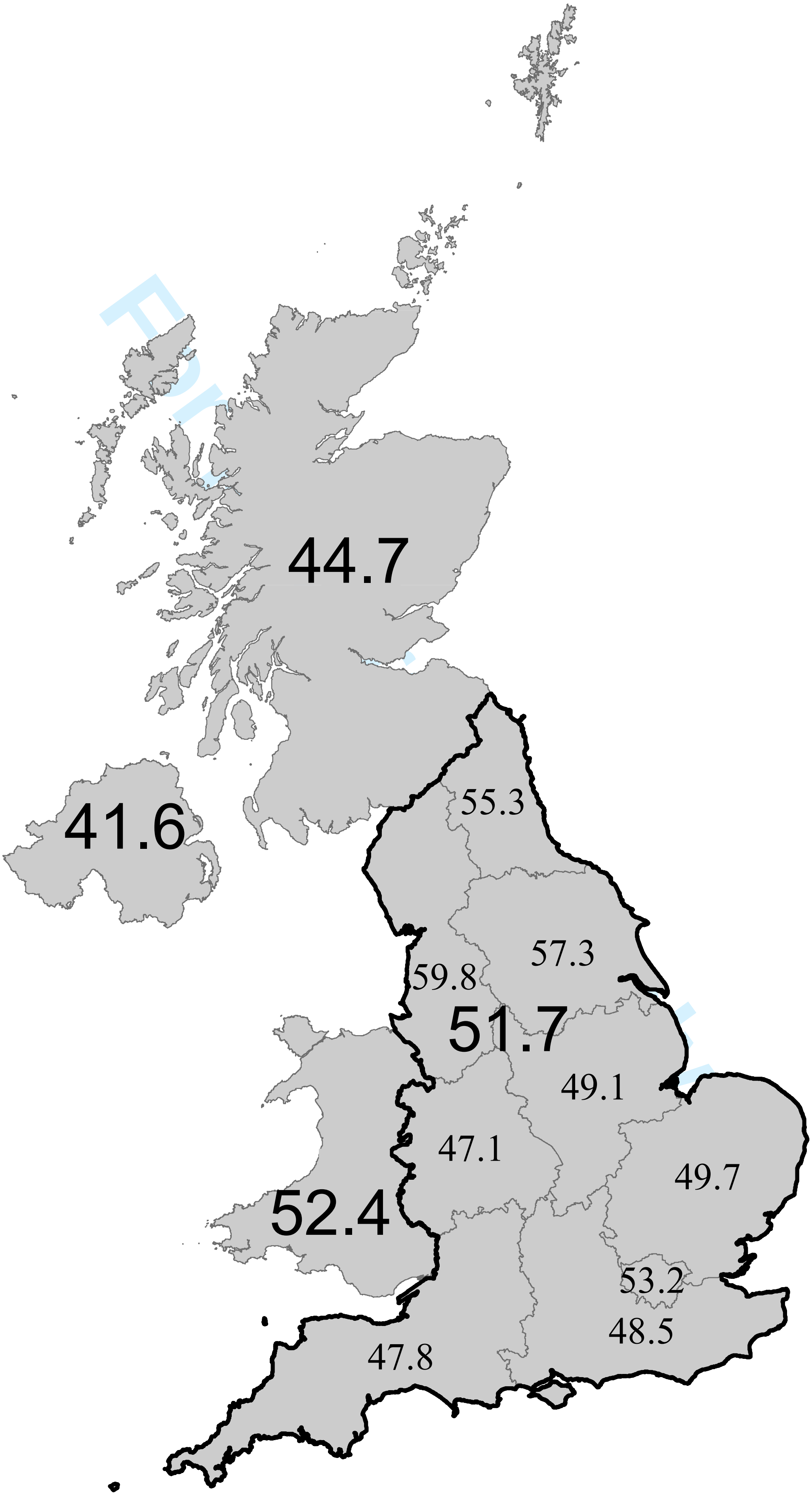
44.7
41.6
55.3
57.3
59.8
51.7
49.1
47.1
49.7
52.4
53.2
48.5
47.8

STROBE Statement—Checklist of items that should be included in reports of ***cohort studies***

	Item No	Recommendation
**Title and abstract**	1 √	(*a*) Indicate the study's design with a commonly used term in the title or the abstract
		(*b*) Provide in the abstract an informative and balanced summary of what was done and what was found
**Introduction**		
Background/rationale	2 √	Explain the scientific background and rationale for the investigation being reported
Objectives	3 √	State specific objectives, including any prespecified hypotheses
**Methods**		
Study design	4 √	Present key elements of study design early in the paper
Setting	5 √	Describe the setting, locations, and relevant dates, including periods of recruitment, exposure, follow-up, and data collection
Participants	6 √	(*a*) Give the eligibility criteria, and the sources and methods of selection of participants. Describe methods of follow-up
		(*b*) For matched studies, give matching criteria and number of exposed and unexposed
Variables	7 √	Clearly define all outcomes, exposures, predictors, potential confounders, and effect modifiers. Give diagnostic criteria, if applicable
Data sources/ measurement	8* √	For each variable of interest, give sources of data and details of methods of assessment (measurement). Describe comparability of assessment methods if there is more than one group
Bias	9 √	Describe any efforts to address potential sources of bias
Study size	10 √	Explain how the study size was arrived at
Quantitative variables	11 √	Explain how quantitative variables were handled in the analyses. If applicable, describe which groupings were chosen and why
Statistical methods	12 √	(*a*) Describe all statistical methods, including those used to control for confounding
		(*b*) Describe any methods used to examine subgroups and interactions
		(*c*) Explain how missing data were addressed
		(*d*) If applicable, explain how loss to follow-up was addressed
		(*e*) Describe any sensitivity analyses
**Results**		
Participants	13* √	(a) Report numbers of individuals at each stage of study—eg numbers potentially eligible, examined for eligibility, confirmed eligible, included in the study, completing follow-up, and analysed
		(b) Give reasons for non-participation at each stage
		(c) Consider use of a flow diagram
Descriptive data	14* √	(a) Give characteristics of study participants (eg demographic, clinical, social) and information on exposures and potential confounders
		(b) Indicate number of participants with missing data for each variable of interest
		(c) Summarise follow-up time (eg, average and total amount)
Outcome data	15* √	Report numbers of outcome events or summary measures over time
Main results	16 √	(*a*) Give unadjusted estimates and, if applicable, confounder-adjusted estimates and their precision (eg, 95% confidence interval). Make clear which confounders were adjusted for and why they were included
		(*b*) Report category boundaries when continuous variables were categorized
		(*c*) If relevant, consider translating estimates of relative risk into absolute risk for a meaningful time period

For peer review only - http://bmjopen.bmj.com/site/about/guidelines.xhtml

Other analyses	17	Report other analyses done—eg analyses of subgroups and interactions, and sensitivity analyses
**Discussion**		
Key results	18 √	Summarise key results with reference to study objectives
Limitations	19 √	Discuss limitations of the study, taking into account sources of potential bias or imprecision. Discuss both direction and magnitude of any potential bias
Interpretation	20 √	Give a cautious overall interpretation of results considering objectives, limitations, multiplicity of analyses, results from similar studies, and other relevant evidence
Generalisability	21 √	Discuss the generalisability (external validity) of the study results
**Other information**		
Funding	22 √	Give the source of funding and the role of the funders for the present study and, if applicable, for the original study on which the present article is based

*Give information separately for exposed and unexposed groups.

Note: An Explanation and Elaboration article discusses each checklist item and gives methodological background and published examples of transparent reporting. The STROBE checklist is best used in conjunction with this article (freely available on the Web sites of PLoS Medicine at http://www.plosmedicine.org/, Annals of Internal Medicine at http://www.annals.org/, and Epidemiology at http://www.epidem.com/). Information on the STROBE Initiative is available at http://www.strobe-statement.org.

For peer review only

For peer review only - http://bmjopen.bmj.com/site/about/guidelines.xhtml

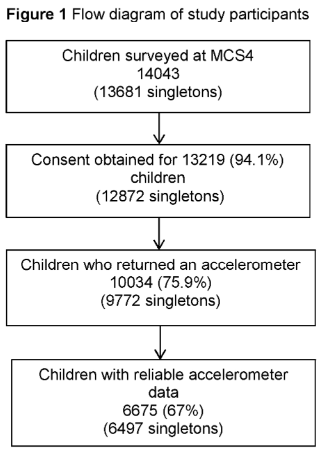

Flow diagram of study participants
209x297mm (300 x 300 DPI)

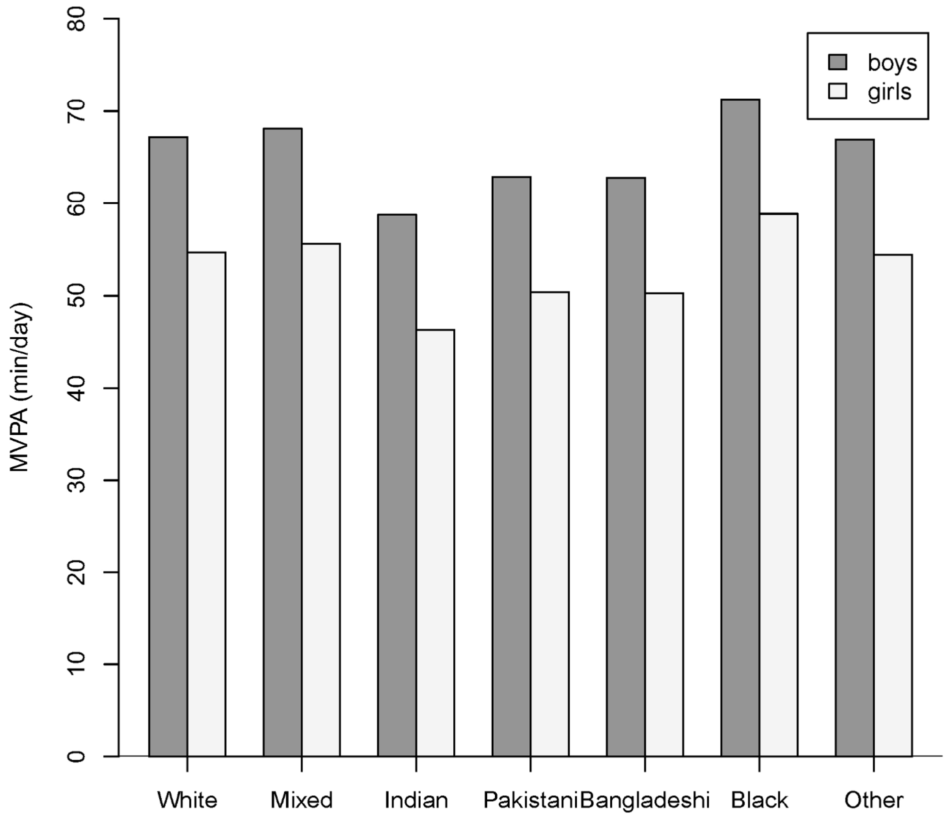

Median minutes per day of MVPA by gender and ethnicity adjusting by season of measurement
177x177mm (300 x 300 DPI)

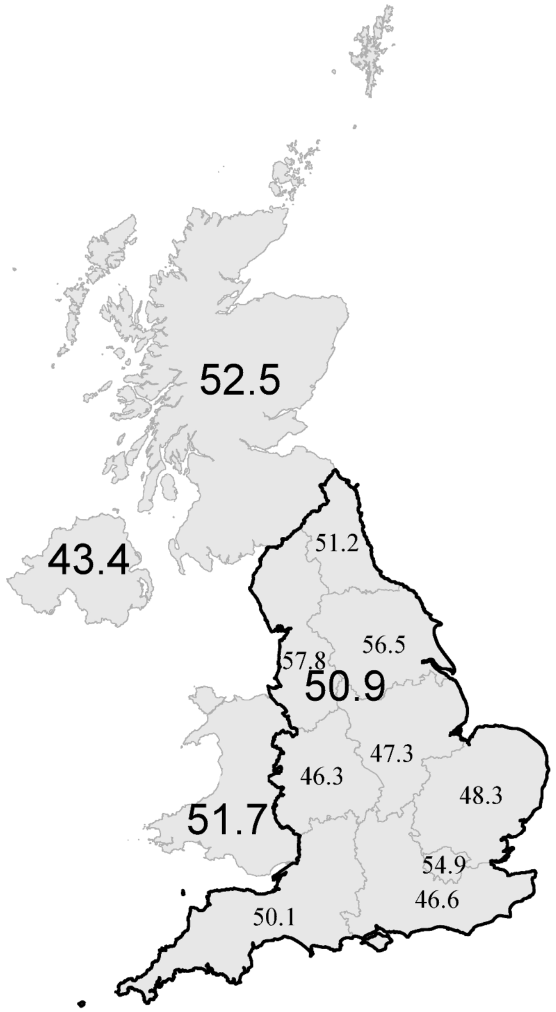

Prevalence of children meeting physical activity recommendation of ≥60 min/day MVPA (%)
210x297mm (300 x 300 DPI)

Manuscript ID bmjopen-2013-002893 entitled "How active are our children? Findings from the Millennium Cohort Study"

Response to Reviewer(s)' Comments to Author:

We thank the reviewers for their constructive comments on our paper, and respond to points made below indicating as appropriate any changes in the manuscript.

Reviewer 1

1. *There are problems related to a comparison of physical activity guidelines derived from self-reported physical activity and levels of PA derived from accelerometer assessed activity, which should be discussed in more detail. The main conclusion is based on this comparison. My main concern is the quantification of children who do not fulfil PA guidelines, because I do not believe half of the population is insufficiently active in a way that compromise their health. The scientific community needs to be more critical with our recommendations if we want to be taken serious, so a thorough discussion of number of children with impaired health that could be attributed to lack of physical activity is needed. The discrepancy could be caused by unrealistic PA guidelines or the cut-point for MVPA you have used. A change in cut-point will change prevalence of sedentary dramatically. The critique may sound severe, but I do think this data is worth publishing, and the study is interesting.*

Response:
We recognise that the national PA recommendations are based on the best available evidence and there is considerable uncertainty as to what the optimum level of PA should be. PA is thought to be important for building cardiovascular fitness as well as for concurrent health. Therefore recommendations for PA reflect estimated optimum levels for current and future health. Furthermore PA should not be thought of as something which simply prevents impaired health but as a health behaviour with potential to promote social, mental and physical wellbeing.

In common with many other accelerometer based studies, we are have reported physical activity and sedentary behaviour using objective rather than proxy reported measures and appraised the adherence to CMO guidelines recognising that the evidence on which the CMO drew comprised proxy report as well as direct measurement. At present the CMO guidelines represent a policy based on best available evidence and we consider it appropriate to appraise adherence to the guideline and any related characteristics of children or their families.

We thank the reviewer for their positive comments regarding our validation study of MVPA and sedentary behaviour in the target age range. We agree that the prevalence of MVPA is sensitive to the threshold used and in our manuscript have reported results for sensitivity analyses for MVPA thresholds of 2000 and 3000 cpm. We agree with the reviewer and had pointed out in the manuscript, that different thresholds do change prevalence estimates of guideline recommendations (page 16 L36-48).

2. *Authors calculate prevalence of participants that fulfill UK guidelines of PA and conclude that population-wide initiatives should be taken to improve PA levels, because half of the population had too little PA. I believe data do not allow such conclusion. The number of children not fulfilling guidelines depends highly on the cutpoint chosen to define MVPA. According to their table 2,*

For peer review only - http://bmjopen.bmj.com/site/about/guidelines.xhtml

prevalenses defer between 0.4% and 94%. I know a validation study has been conducted, but I do not believe that half of the population has a PA level, which cause impaired health, so either guidelines are wrong or cutpoints are wrong. Guidelines have been formed from a different type of data, and conclusions cannot be drawn comparing accelerometer derived MVPA with guidelines formed from self-reported data. Studies have shown that about 10-15% of children have clustering of CVD risk factors, so this does not support that 50% should be inactive. It would make more sense to describe the PA level these kids had in order to recommend how active the population should be. This is not a critique against the present paper, but I think they should refraise their main conclusion. They could also make sensitivity analyses showing how prevalence of sedentary children changed with different MVPA cutpoints such as 2000 cpm and 3000 cpm. All other analyses of associations are still reliable and are not affected by this problem.

Response:

This point has been addressed under (1) above.

In addition we have added a comment in the abstract and key messages sections to emphasise the findings from our original sensitivity analyses as follows:

Page 2: **“Comparison with other similar studies was limited by variation in thresholds used to define MVPA.”**

And

“Longitudinal studies are needed to better understand the relevance of these (in)activity patterns for long term health and well-being.”

And

Page 4 article summary

“• The prevalence of adherence to current recommendations for physical activity is sensitive to the thresholds used to define moderate and vigorous physical activity.”

3. Is it possible in UK to get register data such as SES for all subjects? If this is possible, authors should describe dropout, because a main strength of this type of study is representativeness. In Denmark, we can get access to income, living conditions and much more for all individuals including dropout, but I don’t know enough about UK registers and rules.

Response:
These data are available for cohort participants including those who have been lost to follow up since inception. Please note that survey weights are adjusted for nonresponse and non-compliance to protocol (ie, < 2 days with 10 hours). The variables used to define the weights included household income.

The text has also been amended to provide a reference to a more detailed technical account. (Page 8, paragraph 1; new text in **bold**):

For peer review only - http://bmjopen.bmj.com/site/about/guidelines.xhtml

For peer review only

"Sampling weights were used to account for the stratified clustered design of the MCS. Weights were adjusted for attrition between contacts at successive MCS sweeps and for missing accelerometer data. **Details on the adjustment for non-response and non-compliance are given elsewhere [32]**.

4. P 7, line 25: I am not sure how weighed mean was calculated. The amount of MVPA is highly related to valid observation time, and some children have only been observed for 10h/d while others exceed 16h/d. We don't know if non-wear time is sedentary or MVPA, so we may assume that it could include MVPA (such as swimming). Number of minutes of MVPA should therefore be upgraded in children with short wear-time if data are used to calculate number of children fulfilling guidelines. Another problem related to processing of data is night time. Authors write that children were informed to take the accelerometer off during the night. However, our experience is that many don't do that. It can be solved by visual inspection or by cutting the night away. Including night time will bias counts per minutes but does not affect MVPA.

Response:

The text has been amended and now reads (Page 7, 1st paragraph):

"The start and end dates were detected based on a specified amount of daily waking time (defined as between 07:00 and 21:59) required to be different from zero counts: the threshold for the observational period was set to at least 150 minutes per day. Total registered time is not constant across days. As a result, the total amount of counts and wearing time for each child will depend on how long the accelerometer was worn. These measures were standardised by introducing the notion of a standard day with equal duration (i.e., 735 minutes, equal to mean wear time across all reliable days) (Griffiths et al. 2013)."

Reviewer 2: Claire Nightingale

1. Please state the reasons why only maternal (and not paternal) socioeconomic status was considered in the analysis. In terms of the child's circumstances at home, it would be sensible to consider the socioeconomic status of both parents/guardians unless it is a single parent family. In addition, if only maternal socioeconomic status is available, some comment should be made about potential limitations. For instance, women who chose to stay at home to look after their children would presumably be in the 'never worked/long term unemployed category', however, if the father has a higher socioeconomic status, then the circumstances of the child could potentially be very different to children where both parents are in this category. Therefore if paternal socioeconomic status is available then highest of the maternal or paternal socioeconomic status would be better or if it is not available then please comment on the potential limitations in the discussion.

Response:
This variable is based on the National Statistics Socio-economic Classification (NS-SEC) and is an occupationally based classification. The variable used is maternal current or most recent occupation by child age 7 and the tables and text have been amended accordingly throughout the manuscript.

For peer review only - http://bmjopen.bmj.com/site/about/guidelines.xhtml

Around 6% of mothers are long-term unemployed / never worked and a further 500 mothers are housewives/ stay-at-home-mums at child age 7 (~40% of whom were not employed at MCS4). When data for MCS4 were missing, information was replaced with information from previous sweeps. Among those with both parents currently working at child age 7 there is a tendency for each member of the couple to have jobs at the same general end of the occupational structure. For example, 38% of the dual earners each have jobs within the top two bands. In about one third of cases the NS-SECs are the same, and in the remainder the NS-SEC categories are adjacent. We consider this to be a reasonable indicator of the family's as well as the woman's occupational class situation due to the similarity of partner's NS-SEC.

For more details, please see Table 11.11, Hansen et al. (2010)

http://www.cls.ioe.ac.uk/page.aspx?&sitesectionid=866&sitesectiontitle=The+age+7+survey+of+the+MCS+(2008)

2. *There seems to be some confusion between ethnicity and race in the definition of ethnic groups here. White and black are examples of race/colour and not of ethnicity. Black African, black Caribbean or black British and white British or white European are examples of ethnic groups. Please define ethnic groups appropriately.*

Response:

A footnote on the table defining the ethnic groups included in each category has been added as follows:

"This variable was based on a derived variable from the 2001 Census ethnicity categories indicating the ethnic identity of the children in the MCS data. Aggregate groupings were imposed on these categories to create a smaller manageable number of categories. The names of the groups were also adjusted for reporting. The final ethnic groups were: white (including white-British, -Irish, -Welsh, -Scottish and other white background); Mixed; Indian; Pakistani; Bangladeshi; Black (including Black-Caribbean, Black-African and Black- British); and other (including other Asian, other Black, Chinese and other)."

3. *It is stated in the methods that "Small differences were found in the demographic characteristics of the sample if children with reliable accelerometer data relative to the whole cohort sample". Since this is only a supplementary table it would be helpful to say in the text which characteristics in particular differ if any.*

Response:

The text has been amended to address the reviewers comment and now reads:

"In particular, boys, children living in Wales, those belonging to the Indian, Pakistan/Bangladeshi, and Black Caribbean/African ethnic groups, as well as children belonging to families with a single respondent at the interview showed a lower probability of having a valid accelerometer file".

4. *When examining the association between physical activity variables and socioeconomic status would it seems it would be more appropriate to have a test of heterogeneity and then a test for trend rather than a series of pairwise comparisons since NS-SEC is supposed to have some sort of ordering.*

Response:

For peer review only - http://bmjopen.bmj.com/site/about/guidelines.xhtml

Testing for heterogeneity would produce a significant result and in our view would not add anything to the analyses, since the logical following step would be to perform pairwise comparisons. A pairwise comparison between categories which includes 'extreme' categories is implicitly a test for trend. Adjusted pairwise comparisons allow further tests than those afforded by a set of contrasts, e.g. one baseline category against the rest.

5. *When examining differences between ethnic groups, multiple pairwise comparisons have been carried out with correction for multiple testing. However, since the majority group in this population sample is white (85%), the comparisons between the smaller groups could be underpowered. It may be more appropriate to use the white group as a baseline group for comparisons, since it is the majority in the population.*

Response:

Since these are pairwise comparisons, there is no baseline as such. We did not use the White group as a baseline as this set of contrasts would not allow analysis of all comparisons between categories within each factor. To do this we considered pairwise multiple comparisons adjusted using the Benjamini-Hochberg procedure. This is as stated in the footnote to Table 1, and an example explaining this notation has been added.

P18 footnote to Table 1

"(4) As there are many multiple comparisons among levels of factors included in the models, this table does not show any p-values and only indicates those pairwise differences which were significantly different after applying the Benjamini-Hochberg correction for false discovery rate. We used superindices (a-i) to indicate pairs of groups within a factor that differed significantly pairwise (p<0.05). For example medians of minutes of MVPA differed pairwise between England and Wales (a), England and Scotland (b), and Scotland and Northern Ireland (c)."

6. *In the Results section there is some comment about unadjusted and adjusted analysis but it does not say what was adjusted for. Please state which variables were adjusted for in the methods. Presumably all differences should be adjusted for gender considering there are important differences in physical activity between boys and girls.*

Response

The following text from the footnote to Table 1 has been added to the statistical analysis section (page 9) to make clear which variables were adjusted for.

"Quantile regression models for medians and quartiles were fitted using the R package quantreg[29] to examine differences between socio-demographic groups in terms of time spent in physical (in)activity adjusting for season of measurement."

and

"Prevalences were adjusted for gender, ethnicity, maternal current or most recent occupation, lone parenthood status, number of children in the household, country of residence and season of measurement."

For peer review only - http://bmjopen.bmj.com/site/about/guidelines.xhtml

7. *In Table 1 it would be helpful to have the number of participants in each group in the first column for example the numbers in each ethnic group otherwise the reader has to look at a supplementary table to obtain this information.*

Response

We have updated Table 1 based on reviewer's comment and included a column with the number of participants in each group.

8. *There seems to be a mistake in the percentages given in Figure 1 where it states the number of children who returned an accelerometer was 10034 (79.1%), if this is the response rate for those approached i.e. 10034/14043 then this should be 71.5% or if it is the percentage who returned the accelerometer i.e. 10034/13219 then it should be 75.9%. Please check and correct the numbers in the flow chart.*

Response:

Thank you for noting this error. The absolute figures are correct; the percentage has been corrected in Figure 1 to 75.9%.

Reviewer 3: Ashley Cooper

Page 4/5 *Box: Although the box is helpful in describing what activities are relevant for each intensity level, the MET values are incorrect, since these are given for adults. It is know that the resting energy expenditure of children is higher than adults, so using a value of 3.5mL.kg-1.min-1 is incorrect (the subscript needs to be fully, correctly, defined) and the energy cost of the activities is also wrong (see Harrell et al., MSSE 37 (2), 329-336, 2005).*

Response

We thank the reviewer for this suggestion and have amended the data in the box accordingly and referenced Harrell et al.

Page 6, line 25: *accelerometers were sent and returned in the post. What were the procedures to remove data recorded whilst in transit?*

Response:

The text has been amended to address the reviewer's request to clarify the procedures followed to remove data recorded whilst in transit. It now reads (Page 8):

"A study was performed to discriminate between days when the accelerometer was worn and days when it was likely not to have been worn (e.g. during postal delivery) on the basis of the minimum number of valid minutes per day. The threshold was set to ≥ 150 minutes per day. The first day with registered time exceeding the threshold was set as the start date. The last day with registered time above the threshold was set as end date."

Page 6, line 50: *10 hours of data each day is generally accepted as sufficient to record habitual physical activity. Since journeys to and from school, and physical activity in the period after school*

For peer review only - http://bmjopen.bmj.com/site/about/guidelines.xhtml

contribute substantially to children's overall physical activity, are the results different if only participants with data spanning these periods are included?

Response

We have conducted a detailed reliability analysis with this dataset to define minimum wear time by looking at changes in the intra-class correlation coefficients for different combinations of minimum number of days/minimum number of hours and distinguishing between weekends and weekdays. This paper has been accepted for publication in PLoS One (Rich, C., Geraci, M., Griffiths, L.J., Sera, F., Dezateux, C., Cortina-Borja, M. (2013) Quality control methods in accelerometer data processing: Defining minimum wear time. PLoS One (In press).) In this paper we demonstrated that using accelerometer data from children with measurements from at least two days lasting at least 10 hours/day should provide reliable estimates of PA, and that it is unnecessary to include only children with accelerometer data collected during weekends in analyses.

Page 6, line 50: *although the data are unclear at present, weekday and weekend values for physical activity may differ substantially. How did you address whether the 2 or more days approach included weekend days?*

See response to comment immediately preceding this.

Page 7, line 17: *"optimise the discrimination between activity levels". I am not sure what this means – please clarify.*

Response

The cut-off values referred to in the text were taken from the paper published by Pulsford et al in PLoS One (2010) and were established to identify sedentary behaviour and light, moderate and vigorous physical activity using linear discriminant analysis and evaluated using receiver operating characteristic (ROC) curve analysis.

We have changed the text to clarify this as:

"These cut-off values were established to identify sedentary behaviour and light, moderate and vigorous physical activity using linear discriminant analysis and evaluated using receiver operating characteristic curve analysis, and were taken from a calibration study in seven year olds, carried out specifically for this larger study [25]."

Page 7, line 34: *The authors state that time spent in MVPA was used to determine adherence to the CMO physical activity guidelines. These require at least 60 minutes of MVPA on each day of the week. Given that relatively few children provided 7 days of data, what decisions were made to operationalise these guidelines? Was it 60 minutes of MVPA on each measurement day? A mean of 60 minutes over several days? Please explain in detail your decisions and justification, since the conclusions you draw may be highly dependent on these.*

Response

For each child the dichotomous outcome (does or does not meet CMO physical activity guidelines) was based on the standardised average minutes/day spent in MVPA, where the objective of standardisation was to standardise wearing time across days. This approach is similar to that used in the Gateshead, SPEEDY and ALSPAC studies, presented in table 2. Our rationale was similar to that cited in the ALSPAC study (Riddoch et al 2007), namely that this is a more valid approach than

For peer review only - http://bmjopen.bmj.com/site/about/guidelines.xhtml

scrutinising each individual day as this would disadvantage children who achieved well in excess of 60 min on one day but who may have narrowly failed to achieve 60 min on another day.

This has been added to the statistical methods section as follows:

"For each child time spent in MVPA was used to determine adherence to the CMO physical activity guideline (≥60 minutes daily) and was based on the standardised average minutes/day spent in MVPA, where the objective of standardisation was to standardise wearing time across days."

Page 7, line 44: *Since BMI and season are known to influence children's physical activity, why were these not included as explanatory variables.*

Response

We thank the reviewer for highlighting this important point. Our study confirms, as reported by ourselves (Rich et al 2012) and others, that season of year is an important determinant of PA and SB behaviour with higher levels of PA observed in Spring and Summer with respect to Winter and Autumn (p<0.05). The following table shows the sample sizes, medians, IQRs and prevalences with their standard errors by season.

Season	*n*					
Winter	872	545<br>461, 647	6.7<br>6.2, 7.3	52.8<br>42.1,<br>68.1	9491<br>7982,<br>10728	38.8<br>(0.029)
Spring	656	651<br>563, 759	6.3<br>5.8, 6.8	67.6<br>54.4,<br>84.1	11178<br>9745,<br>12718	65.6<br>(0.023)
Summer	2790	618<br>522, 727	6.4<br>5.8, 6.9	63.3<br>49.2,<br>79.7	10533<br>8909,<br>12408	55.8<br>(0.012)
Autumn	2179	558<br>483, 650	6.7<br>6.2, 7.2	56.7<br>44.5,<br>70.8	9709<br>8471,<br>11244	43.5<br>(0.013)

Due to the order in which the fieldwork was carried out, distribution of season of measurement was not equally distributed across all four UK countries. Children living in Scotland and Northern Ireland were mostly measured during the winter and autumn seasons (84% and 53% of the measurements, respectively), while children living in England and Wales were mostly measured during spring and

For peer review only - http://bmjopen.bmj.com/site/about/guidelines.xhtml

summer seasons (60% and 54% of the measurements, respectively). As a result season of measurement is confounded with country of residence in the Millennium Cohort Study. We have now adjusted by season of measurement the medians and IQRs reported in Table 1. The main change to the data occurring as a consequence of this adjustment is that estimates of PA and SB in Scotland which were previously reported as significant with respect to England are now no longer significant. Table 1 and the text have been changed accordingly.

The following text has been inserted on page 8

Yearly seasons of measurement were defined astronomically, i.e. spring (21st March –20th June), summer (21st June – 20th September), autumn (21st September – 20th December), winter (21st December – 20th March).

Page 8, line 11: *Percentages of what were derived?*

Response

The text has been amended (page 9) and now reads:

"Prevalences of the children meeting the CMO current physical activity guideline were derived, as were medians and interquartile ranges for derived activity variables (given their non-normal distributions)."

Page 8, line 13: *I am surprised that accelerometer cpm were not Normally distributed. I would expect MVPA to be skewed, but my experience is that cpm are usually Normal.*

Response

The sentence about skewness has been amended to clarify the use of median regression. Skewness is only one violation of normality. Heavy tails and heteroscedasticity also represent other violations. All PA outcomes, including cpm, violated normality for one reason or another. The distribution of MVPA and cpm variables were not normal according the Shapiro-Francia *W* test for normality (p<0.05). Median regression is a robust approach since inference does not depend on distributional assumptions. It is also robust to outliers.

The text has been amended to read:

"These models do not make assumptions about the shape of the distribution. Therefore they are robust to departures from normality, as for example skewness, heavy-tailedness and heteroscedasticity, which affected all PA outcomes in this study."

Page 9, line 16: *there are relatively few children in some of the ethnic groups. How confident are you that you can make robust claims about the association between ethnicity and physical activity.*

Response

We considered all comparisons between ethnic categories using pairwise multiple comparisons adjusted using the Benjamini-Hochberg procedure as stated in the footnote to Table 1. We feel confident that the associations observed between ethnicity and physical activity are robust.

For peer review only - http://bmjopen.bmj.com/site/about/guidelines.xhtml

For peer review only

Page 15, line 58: *The derived threshold for MVPA is similar to that of Evenson et al, recently shown to have to have the best classification accuracy for MVPA by Trost et al (MSSE 2011). This may be worth citing to support use of your own threshold.*

We thank the reviewer for this suggestion. Evenson suggests a threshold for MVPA of ≥2296cpm which is indeed similar to that derived in our study. This paper has now been cited in support of the threshold we derived:

"They are similar to those reported by Evenson et al [26]."

Page 16, line 9: *the underestimation in cycling is not a consequence of the accelerometers used, but rather the positioning of the accelerometers ie worn on a belt around the waist. Given that cycling is a rather infrequent activity, the (likely) small underestimation is probably of little consequence – this could be discussed. Possibly of more importance is the possible removal or under-reporting during other activities such as gym or contact sports.*

Response

We thank the reviewer for this correction and have added on page 16 the following phrase (shown in **bold**):

"Acknowledged limitations include potential underestimation of physical activity, as a result of the accelerometers used, the position in which they are worn, or their removal during some activities such as aquatic activities or contact sports."

Page 18, table: *the Wheeler reference is the percentage of time spent in MVPA, not the proportion meeting guidelines.*

We thank the reviewer for this correction and have removed reference to this paper from the Table.

Page 18, table footnote: *do you mean a mean of 60 or more minutes of MVPA per day?*

Response

We thank the reviewer for this observation. The exact measure used in defining adherence to the 60 minute guideline varies across the studies tabulated: MCS, Gateshead, SPEEDY and ALSPAC used a mean of 60 minutes or more, the CHASE study just the first day of measurement, and the Health Survey calculating on a subsample with 7 days of observation, and defined as at least of 60 minutes of MVPA per day on all seven days of observation. We have added the following comment to the Table 2 footnote (p 21):

"Table footnote: *Accumulating ≥60 min/day MVPA The exact measure used in defining adherence to the 60 minute guideline varies across the studies tabulated: MCS, Gateshead, SPEEDY and ALSPAC used a mean of 60 minutes or more, the CHASE study just the first day of measurement, and the Health Survey included a subsample with seven days of observation each with at least 60 minutes of MVPA per day."

Page 19, line 38: *discussion around the possible causes of the ethnic and socioeconomic differences is quite minimal. Why, for example, should the less advantaged children be slightly more active? Are*

For peer review only - http://bmjopen.bmj.com/site/about/guidelines.xhtml

country/regional differences actually due to the different environments (urban vs rural). This is touched upon on page 21, but maybe could have been addressed here?

We are unsure of the reasons for the ethnic differences observed in this study but now suggest that future research is required to explore different cultural reasons, for example, for these.

Page 20, line 3: *estimates of sedentary time will depend upon the measurement period and decisions made about removal of zero values. Discussion here could be extended to compare the parameters you used with other studies – this may explain discrepancies.*

We agree with the reviewer however previous authors have not always clearly stated the approach used. We have added the following text to page 23 as a comment to acknowledge this issue:

"Differences in estimates of sedentary time and numbers of steps between studies may reflect measurement period while decisions made about removal of zero values may also influence sedentary time. These parameters are not consistently reported in published reports."

Page 20, line 17: *similarly, steps recorded will depend on the measurement period.*

See response to immediately preceding comment.

For peer review only

For peer review only - http://bmjopen.bmj.com/site/about/guidelines.xhtml
