# Supplementary material for: How active are our children? Findings from the Millennium Cohort Study
Source: BMJ Open. 2013 Jul 25;3(8):e002893. doi: 10.1136/bmjopen-2013-002893 (PMC3752053; doi:10.1136/bmjopen-2013-002893)
Supplement: Reviewer comments [file bmjopen-2013-002893.reviewer_comments.pdf]

## PEER REVIEW HISTORY

BMJ Open publishes all reviews undertaken for accepted manuscripts. Reviewers are asked to complete a checklist review form (see an example) and are provided with free text boxes to elaborate on their assessment. These free text comments are reproduced below. Some articles will have been accepted based in part or entirely on reviews undertaken for other BMJ Group journals. These will be reproduced where possible.

## ARTICLE DETAILS

| | |
|---|---|
| **TITLE (PROVISIONAL)** | How active are our children? Findings from the Millennium Cohort Study |
| **AUTHORS** | Griffiths, Lucy; Cortina Borja, Mario; Sera, Francesco; Pouliou, Theodora; Geraci, Marco; Rich, Carly; Cole, Tim; Law, Catherine; Joshi, Heather; Ness, Andy; Jebb, Susan; Dezateux, Carol |

## VERSION 1 - REVIEW

| | |
|---|---|
| **REVIEWER** | Lars Bo Andersen<br>Professor and Head of Research Unit, Institute of Sports Science and Clinical Biomechanics<br><br>No competing Interests |
| **REVIEW RETURNED** | 24-Mar-2013 |

| | |
|---|---|
| **THE STUDY** | There are problems related to a comparison of physical activity guidelines derived from self-reported physical activity and levels of PA derived from accelerometer assessed activity, which should be discussed in more detail. The main conclusion is based on this comparison. See my more specific comments below |
| **RESULTS & CONCLUSIONS** | I have attached more detailed information below.<br>My main concern is the quantification of children who do not fulfill PA guidelines, because I do not believe half of the population is insufficiently active in a way that compromise their health. The scientific community needs to be more critical with our recommendations if we want to be taken serious, so a thorough discussion of number of children with impaired health that could be attributed to lack of physical activity is needed. The discrepancy could be caused by unrealistic PA guidelines or the cutpoint for MVPA you have used. A change in cutpoint will change prevalense of sedentary dramatically. The critique may sound severe, but I do think this data is worth publishing, and the study is interesting. |
| **GENERAL COMMENTS** | This is a large cohort study describing accelerometer measured physical activity levels in 7-yr old children. It contains valuable information about associations between PA level and different background variables such as ethnicity, sex, SES and geography. The study is in general well conducted and authors should be acknowledged for a good validation study has been performed earlier to define MVPA in the target age group. However, there are a number of concerns that need to be discussed in more detail and data does not allow to draw the main conclusion.<br>Authors calculate prevalence of participants that fulfill UK guidelines of PA and conclude that population-wide initiatives should be taken to improve PA levels, because half of the population had too little PA. I believe data do not allow such conclusion. The number of children not fulfilling guidelines depends highly on the cutpoint chosen to define MVPA. According to their table 2, prevalenses defer between 0.4% and 94%. I know a validation study has been |

| | |
|---|---|
| | conducted, but I do not believe that half of the population has a PA level, which cause impaired health, so either guidelines are wrong or cutpoints are wrong. Guidelines have been formed from a different type of data, and conclusions cannot be drawn comparing accelerometer derived MVPA with guidelines formed from self-reported data. Studies have shown that about 10-15% of children have clustering of CVD risk factors, so this does not support that 50% should be inactive. It would make more sense to describe the PA level these kids had in order to recommend how active the population should be. This is not a critique against the present paper, but I think they should refraise their main conclusion. They could also make sensitivity analyses showing how prevalence of sedentary children changed with different MVPA cutpoints such as 2000 cpm and 3000 cpm. All other analyses of associations are still reliable and are not affected by this problem.<br>Is it possible in UK to get register data such as SES for all subjects? If this is possible, authors should describe dropout, because a main strength of this type of study is representativeness. In Denmark, we can get access to income, living conditions and much more for all individuals including dropout, but I don't know enough about UK registers and rules.<br>P 7, line 25: I am not sure how weighed mean was calculated. The amount of MVPA is highly related to valid observation time, and some children have only been observed for 10h/d while others exceed 16h/d. We don't know if non-wear time is sedentary or MVPA, so we may assume that it could include MVPA (such as swimming). Number of minutes of MVPA should therefore be upgraded in children with short wear-time if data are used to calculate number of children fulfilling guidelines. Another problem related to processing of data is night time. Authors write that children were informed to take the accelerometer off during the night. However, our experience is that many don't do that. It can be solved by visual inspection or by cutting the night away. Including night time will bias counts per minutes but does not affect MVPA. |

| | |
|---|---|
| **REVIEWER** | Claire Nightingale,<br>NIHR Doctoral Research Fellow.<br>St. George's, University of London,<br>United Kingdom. |
| **REVIEW RETURNED** | 28-Mar-2013 |

| | |
|---|---|
| **THE STUDY** | 1. Please state the reasons why only maternal (and not paternal) socioeconomic status was considered in the analysis. In terms of the child's circumstances at home, it would be sensible to consider the socioeconomic status of both parents/guardians unless it is a single parent family. In addition, if only maternal socioeconomic status is available, some comment should be made about potential limitations. For instance, women who chose to stay at home to look after their children would presumably be in the 'never worked/long term unemployed category', however, if the father has a higher socioeconomic status, then the circumstances of the child could potentially be very different to children where both parents are in this category. Therefore if paternal socioeconomic status is available then highest of the maternal or paternal socioeconomic status would be better or if it is not available then please comment on the potential limitations in the discussion.<br><br>2. There seems to be some confusion between ethnicity and race in |

| | |
|---|---|
| | the definition of ethnic groups here. White and black are examples of race/colour and not of ethnicity. Black African, black Caribbean or black British and white British or white European are examples of ethnic groups. Please define ethnic groups appropriately.<br><br>3. It is stated in the methods that "Small differences were found in the demographic characteristics of the sample if children with reliable accelerometer data relative to the whole cohort sample". Since this is only a supplementary table it would be helpful to say in the text which characteristics in particular differ if any. |
| **RESULTS & CONCLUSIONS** | 4. When examining the association between physical activity variables and socioeconomic status would it seems it would be more appropriate to have a test of heterogeneity and then a test for trend rather than a series of pairwise comparisons since NS-SEC is supposed to have some sort of ordering.<br><br>5. When examining differences between ethnic groups, multiple pairwise comparisons have been carried out with correction for multiple testing. However, since the majority group in this population sample is white (85%), the comparisons between the smaller groups could be underpowered. It may be more appropriate to use the white group as a baseline group for comparisons, since it is the majority in the population.<br><br>6. In the Results section there is some comment about unadjusted and adjusted analysis but it does not say what was adjusted for. Please state which variables were adjusted for in the methods. Presumably all differences should be adjusted for gender considering there are important differences in physical activity between boys and girls.<br><br>7. In Table 1 it would be helpful to have the number of participants in each group in the first column for example the numbers in each ethnic group otherwise the reader has to look at a supplementary table to obtain this information.<br><br>8. There seems to be a mistake in the percentages given in Figure 1 where it states the number of children who returned an accelerometer was 10034 (79.1%), if this is the response rate for those approached i.e. 10034/14043 then this should be 71.5% or if it is the percentage who returned the accelerometer i.e. 10034/13219 then it should be 75.9%. Please check and correct the numbers in the flow chart. |
| **GENERAL COMMENTS** | This paper describes levels of objectively measured physical activity (and inactivity) in 7-8 year-old UK children who took part in the Millennium Cohort Study. It also examines differences in physical activity (and inactivity) between boys and girls, UK countries, ethnic groups, socioeconomic groups and other demographic characteristics. The main findings were that a high proportion of children are not meeting the CMO recommendations for time spent in moderate to vigorous activity and are spending large amounts of time being sedentary. In addition boys were more active than girls. This is a large study which has the advantage of having objectively measured physical activity using accelerometers rather than self-reported physical activity as well good coverage across the UK. The paper is well written throughout; however I have minor comments about the methodology and statistical analysis which are outlined below. |

| | |
|---|---|
| **REVIEWER** | Ashley Cooper<br>Professor of Physical Activity and Public Health<br>Centre for Exercise, Nutrition and Health Sciences<br>School for Policy Studies<br>University of Bristol<br><br>I confirm that I have no competing interests. |
| **REVIEW RETURNED** | 16-Apr-2013 |

| | |
|---|---|
| **GENERAL COMMENTS** | Page 4/5 Box: Although the box is helpful in describing what activities are relevant for each intensity level, the MET values are incorrect, since these are given for adults. It is know that the resting energy expenditure of children is higher than adults, so using a value of 3.5mL.kg-1.min-1 is incorrect (the subscript needs to be fully, correctly, defined) and the energy cost of the activities is also wrong (see Harrell et al., MSSE 37 (2), 329-336, 2005).<br><br>Page 6, line 25: accelerometers were sent and returned in the post. What were the procedures to remove data recorded whilst in transit?<br><br>Page 6, line 50: 10 hours of data each day is generally accepted as sufficient to record habitual physical activity. Since journeys to and from school, and physical activity in the period after school contribute substantially to children's overall physical activity, are the results different if only participants with data spanning these periods are included?<br><br>Page 6, line 50: although the data are unclear at present, weekday and weekend values for physical activity may differ substantially. How did you address whether the 2 or more days approach included weekend days?<br><br>Page 7, line 17: "optimise the discrimination between activity levels". I am not sure what this means – please clarify.<br><br>Page 7, line 34: The authors state that time spent in MVPA was used to determine adherence to the CMO physical activity guidelines. These require at least 60 minutes of MVPA on each day of the week. Given that relatively few children provided 7 days of data, what decisions were made to operationalise these guidelines? Was it 60 minutes of MVPA on each measurement day? A mean of 60 minutes over several days? Please explain in detail your decisions and justification, since the conclusions you draw may be highly dependent on these.<br><br>Page 7, line 44: Since BMI and season are known to influence children's physical activity, why were these not included as explanatory variables.<br><br>Page 8, line 11: Percentages of what were derived?<br><br>Page 8, line 13: I am surprised that accelerometer cpm were not Normally distributed. I would expect MVPA to be skewed, but my experience is that cpm are usually Normal.<br><br>Page9, line 16: there are relatively few children in some of the ethnic groups. How confident are you that you can make robust claims about the association between ethnicity and physical activity. |

| | |
|---|---|
| | Page 15, line 58: The derived threshold for MVPA is similar to that of Evenson et al, recently shown to have to have the best classification accuracy for MVPA by Trost et al (MSSE 2011). This may be worth citing to support use of your own threshold.<br><br>Page 16, line 9: the underestimation in cycling is not a consequence of the accelerometers used, but rather the positioning of the accelerometers ie worn on a belt around the waist. Given that cycling is a rather infrequent activity, the (likely) small underestimation is probably of little consequence – this could be discussed. Possibly of more importance is the possible removal or under-reporting during other activities such as gym or contact sports.<br><br>Page 18, table: the Wheeler reference is the percentage of time spent in MVPA, not the proportion meeting guidelines.<br><br>Page 18, table footnote: do you mean a mean of 60 or more minutes of MVPA per day?<br><br>Page 19, line 38: discussion around the possible causes of the ethnic and socioeconomic differences is quite minimal. Why, for example, should the less advantaged children be slightly more active? Are country/regional differences actually due to the different environments (urban vs rural). This is touched upon on page 21, but maybe could have been addressed here?<br><br>Page 20, line 3: estimates of sedentary time will depend upon the measurement period and decisions made about removal of zero values. Discussion here could be extended to compare the parameters you used with other studies – this may explain discrepancies.<br><br>Page 20, line 17: similarly, steps recorded will depend on the measurement period. |

**VERSION 1 – AUTHOR RESPONSE**

Reviewer 1
1. There are problems related to a comparison of physical activity guidelines derived from self-reported physical activity and levels of PA derived from accelerometer assessed activity, which should be discussed in more detail. The main conclusion is based on this comparison. My main concern is the quantification of children who do not fulfil PA guidelines, because I do not believe half of the population is insufficiently active in a way that compromise their health. The scientific community needs to be more critical with our recommendations if we want to be taken serious, so a thorough discussion of number of children with impaired health that could be attributed to lack of physical activity is needed. The discrepancy could be caused by unrealistic PA guidelines or the cut-point for MVPA you have used. A change in cut-point will change prevalence of sedentary dramatically. The critique may sound severe, but I do think this data is worth publishing, and the study is interesting.
Response:
We recognise that the national PA recommendations are based on the best available evidence and there is considerable uncertainty as to what the optimum level of PA should be. PA is thought to be important for building cardiovascular fitness as well as for concurrent health. Therefore recommendations for PA reflect estimated optimum levels for current and future health. Furthermore PA should not be thought of as something which simply prevents impaired health but as a health behaviour with potential to promote social, mental and physical wellbeing.
In common with many other accelerometer based studies, we are have reported physical activity and

sedentary behaviour using objective rather than proxy reported measures and appraised the adherence to CMO guidelines recognising that the evidence on which the CMO drew comprised proxy report as well as direct measurement. At present the CMO guidelines represent a policy based on best available evidence and we consider it appropriate to appraise adherence to the guideline and any related characteristics of children or their families.
We thank the reviewer for their positive comments regarding our validation study of MVPA and sedentary behaviour in the target age range. We agree that the prevalence of MVPA is sensitive to the threshold used and in our manuscript have reported results for sensitivity analyses for MVPA thresholds of 2000 and 3000 cpm. We agree with the reviewer and had pointed out in the manuscript, that different thresholds do change prevalence estimates of guideline recommendations (page 16 L36-48).
2. Authors calculate prevalence of participants that fulfill UK guidelines of PA and conclude that population-wide initiatives should be taken to improve PA levels, because half of the population had too little PA. I believe data do not allow such conclusion. The number of children not fulfilling guidelines depends highly on the cutpoint chosen to define MVPA. According to their table 2, prevalenses defer between 0.4% and 94%. I know a validation study has been conducted, but I do not believe that half of the population has a PA level, which cause impaired health, so either guidelines are wrong or cutpoints are wrong. Guidelines have been formed from a different type of data, and conclusions cannot be drawn comparing accelerometer derived MVPA with guidelines formed from self-reported data. Studies have shown that about 10-15% of children have clustering of CVD risk factors, so this does not support that 50% should be inactive. It would make more sense to describe the PA level these kids had in order to recommend how active the population should be. This is not a critique against the present paper, but I think they should refraise their main conclusion. They could also make sensitivity analyses showing how prevalence of sedentary children changed with different MVPA cutpoints such as 2000 cpm and 3000 cpm. All other analyses of associations are still reliable and are not affected by this problem.
Response:
This point has been addressed under (1) above.
In addition we have added a comment in the abstract and key messages sections to emphasise the findings from our original sensitivity analyses as follows:
Page 2: “Comparison with other similar studies was limited by variation in thresholds used to define MVPA.”
And
“Longitudinal studies are needed to better understand the relevance of these (in)activity patterns for long term health and well-being.”
And
Page 4 article summary
“• The prevalence of adherence to current recommendations for physical activity is sensitive to the thresholds used to define moderate and vigorous physical activity.”
3. Is it possible in UK to get register data such as SES for all subjects? If this is possible, authors should describe dropout, because a main strength of this type of study is representativeness. In Denmark, we can get access to income, living conditions and much more for all individuals including dropout, but I don’t know enough about UK registers and rules.

Response:
These data are available for cohort participants including those who have been lost to follow up since inception. Please note that survey weights are adjusted for nonresponse and non-compliance to protocol (ie, < 2 days with 10 hours). The variables used to define the weights included household income.

The text has also been amended to provide a reference to a more detailed technical account. (Page 8, paragraph 1; new text in bold):

“Sampling weights were used to account for the stratified clustered design of the MCS. Weights were adjusted for attrition between contacts at successive MCS sweeps and for missing accelerometer data. Details on the adjustment for non-response and non-compliance are given elsewhere [32].

4. P 7, line 25: I am not sure how weighed mean was calculated. The amount of MVPA is highly related to valid observation time, and some children have only been observed for 10h/d while others exceed 16h/d. We don’t know if non-wear time is sedentary or MVPA, so we may assume that it could include MVPA (such as swimming). Number of minutes of MVPA should therefore be upgraded in children with short wear-time if data are used to calculate number of children fulfilling guidelines. Another problem related to processing of data is night time. Authors write that children were informed to take the accelerometer off during the night. However, our experience is that many don’t do that. It can be solved by visual inspection or by cutting the night away. Including night time will bias counts per minutes but does not affect MVPA.

Response:
The text has been amended and now reads (Page 7, 1st paragraph):
“The start and end dates were detected based on a specified amount of daily waking time (defined as between 07:00 and 21:59) required to be different from zero counts: the threshold for the observational period was set to at least 150 minutes per day. Total registered time is not constant across days. As a result, the total amount of counts and wearing time for each child will depend on how long the accelerometer was worn. These measures were standardised by introducing the notion of a standard day with equal duration (i.e., 735 minutes, equal to mean wear time across all reliable days) (Griffiths et al. 2013).”
Reviewer 2: Claire Nightingale
1. Please state the reasons why only maternal (and not paternal) socioeconomic status was considered in the analysis. In terms of the child’s circumstances at home, it would be sensible to consider the socioeconomic status of both parents/guardians unless it is a single parent family. In addition, if only maternal socioeconomic status is available, some comment should be made about potential limitations. For instance, women who chose to stay at home to look after their children would presumably be in the ‘never worked/long term unemployed category’, however, if the father has a higher socioeconomic status, then the circumstances of the child could potentially be very different to children where both parents are in this category. Therefore if paternal socioeconomic status is available then highest of the maternal or paternal socioeconomic status would be better or if it is not available then please comment on the potential limitations in the discussion.
Response:
This variable is based on the National Statistics Socio-economic Classification (NS-SEC) and is an occupationally based classification. The variable used is maternal current or most recent occupation by child age 7 and the tables and text have been amended accordingly throughout the manuscript.

Around 6% of mothers are long-term unemployed / never worked and a further 500 mothers are housewives/ stay-at-home-mums at child age 7 (~40% of whom were not employed at MCS4). When data for MCS4 were missing, information was replaced with information from previous sweeps. Among those with both parents currently working at child age 7 there is a tendency for each member of the couple to have jobs at the same general end of the occupational structure. For example, 38% of the dual earners each have jobs within the top two bands. In about one third of cases the NS-SECs are the same, and in the remainder the NS-SEC categories are adjacent. We consider this to be a reasonable indicator of the family's as well as the woman's occupational class situation due to the similarity of partner's NS-SEC.
For more details, please see Table 11.11, Hansen et al. (2010)
http://www.cls.ioe.ac.uk/page.aspx?&sitesectionid=866&sitesectiontitle=The+age+7+survey+of+the+MCS+(2008)
2. There seems to be some confusion between ethnicity and race in the definition of ethnic groups

here. White and black are examples of race/colour and not of ethnicity. Black African, black Caribbean or black British and white British or white European are examples of ethnic groups. Please define ethnic groups appropriately.
Response:
A footnote on the table defining the ethnic groups included in each category has been added as follows:
"This variable was based on a derived variable from the 2001 Census ethnicity categories indicating the ethnic identity of the children in the MCS data. Aggregate groupings were imposed on these categories to create a smaller manageable number of categories. The names of the groups were also adjusted for reporting. The final ethnic groups were: white (including white-British, -Irish, -Welsh, -Scottish and other white background); Mixed; Indian; Pakistani; Bangladeshi; Black (including Black-Caribbean, Black-African and Black- British); and other (including other Asian, other Black, Chinese and other)."
3. It is stated in the methods that "Small differences were found in the demographic characteristics of the sample if children with reliable accelerometer data relative to the whole cohort sample". Since this is only a supplementary table it would be helpful to say in the text which characteristics in particular differ if any.
Response:

The text has been amended to address the reviewers comment and now reads:

"In particular, boys, children living in Wales, those belonging to the Indian, Pakistan/Bangladeshi, and Black Caribbean/African ethnic groups, as well as children belonging to families with a single respondent at the interview showed a lower probability of having a valid accelerometer file".

4. When examining the association between physical activity variables and socioeconomic status would it seems it would be more appropriate to have a test of heterogeneity and then a test for trend rather than a series of pairwise comparisons since NS-SEC is supposed to have some sort of ordering.
Response:
Testing for heterogeneity would produce a significant result and in our view would not add anything to the analyses, since the logical following step would be to perform pairwise comparisons. A pairwise comparison between categories which includes 'extreme' categories is implicitly a test for trend. Adjusted pairwise comparisons allow further tests than those afforded by a set of contrasts, e.g. one baseline category against the rest.
5. When examining differences between ethnic groups, multiple pairwise comparisons have been carried out with correction for multiple testing. However, since the majority group in this population sample is white (85%), the comparisons between the smaller groups could be underpowered. It may be more appropriate to use the white group as a baseline group for comparisons, since it is the majority in the population.
Response:
Since these are pairwise comparisons, there is no baseline as such. We did not use the White group as a baseline as this set of contrasts would not allow analysis of all comparisons between categories within each factor. To do this we considered pairwise multiple comparisons adjusted using the Benjamini-Hochberg procedure. This is as stated in the footnote to Table 1, and an example explaining this notation has been added.
P18 footnote to Table 1
"(4) As there are many multiple comparisons among levels of factors included in the models, this table does not show any p-values and only indicates those pairwise differences which were significantly different after applying the Benjamini-Hochberg correction for false discovery rate. We used superindices (a-i) to indicate pairs of groups within a factor that differed significantly pairwise ($p<0.05$). For example medians of minutes of MVPA differed pairwise between England and Wales (a), England

and Scotland (b), and Scotland and Northern Ireland (c)."

6. In the Results section there is some comment about unadjusted and adjusted analysis but it does not say what was adjusted for. Please state which variables were adjusted for in the methods. Presumably all differences should be adjusted for gender considering there are important differences in physical activity between boys and girls.

Response

The following text from the footnote to Table 1 has been added to the statistical analysis section (page 9) to make clear which variables were adjusted for.

"Quantile regression models for medians and quartiles were fitted using the R package quantreg29 to examine differences between socio-demographic groups in terms of time spent in physical (in)activity adjusting for season of measurement."

and

"Prevalences were adjusted for gender, ethnicity, maternal current or most recent occupation, lone parenthood status, number of children in the household, country of residence and season of measurement."

7. In Table 1 it would be helpful to have the number of participants in each group in the first column for example the numbers in each ethnic group otherwise the reader has to look at a supplementary table to obtain this information.

Response

We have updated Table 1 based on reviewer's comment and included a column with the number of participants in each group.

8. There seems to be a mistake in the percentages given in Figure 1 where it states the number of children who returned an accelerometer was 10034 (79.1%), if this is the response rate for those approached i.e. 10034/14043 then this should be 71.5% or if it is the percentage who returned the accelerometer i.e. 10034/13219 then it should be 75.9%. Please check and correct the numbers in the flow chart.

Response:

Thank you for noting this error. The absolute figures are correct; the percentage has been corrected in Figure 1 to 75.9%.

Reviewer 3: Ashley Cooper

Page 4/5 Box: Although the box is helpful in describing what activities are relevant for each intensity level, the MET values are incorrect, since these are given for adults. It is know that the resting energy expenditure of children is higher than adults, so using a value of 3.5mL.kg-1.min-1 is incorrect (the subscript needs to be fully, correctly, defined) and the energy cost of the activities is also wrong (see Harrell et al., MSSE 37 (2), 329-336, 2005).

Response

We thank the reviewer for this suggestion and have amended the data in the box accordingly and referenced Harrell et al.

Page 6, line 25: accelerometers were sent and returned in the post. What were the procedures to remove data recorded whilst in transit?

Response:

The text has been amended to address the reviewer's request to clarify the procedures followed to remove data recorded whilst in transit. It now reads (Page 8):

"A study was performed to discriminate between days when the accelerometer was worn and days when it was likely not to have been worn (e.g. during postal delivery) on the basis of the minimum number of valid minutes per day. The threshold was set to ≥ 150 minutes per day. The first day with registered time exceeding the threshold was set as the start date. The last day with registered time above the threshold was set as end date."

Page 6, line 50: 10 hours of data each day is generally accepted as sufficient to record habitual physical activity. Since journeys to and from school, and physical activity in the period after school contribute substantially to children's overall physical activity, are the results different if only participants with data spanning these periods are included?

Response
We have conducted a detailed reliability analysis with this dataset to define minimum wear time by looking at changes in the intra-class correlation coefficients for different combinations of minimum number of days/minimum number of hours and distinguishing between weekends and weekdays. This paper has been accepted for publication in PLoS One (Rich, C., Geraci, M., Griffiths, L.J., Sera, F., Dezateux, C., Cortina-Borja, M. (2013) Quality control methods in accelerometer data processing: Defining minimum wear time. PLoS One (In press). ) In this paper we demonstrated that using accelerometer data from children with measurements from at least two days lasting at least 10 hours/day should provide reliable estimates of PA, and that it is unnecessary to include only children with accelerometer data collected during weekends in analyses.
Page 6, line 50: although the data are unclear at present, weekday and weekend values for physical activity may differ substantially. How did you address whether the 2 or more days approach included weekend days?
See response to comment immediately preceding this.
Page 7, line 17: “optimise the discrimination between activity levels”. I am not sure what this means – please clarify.
Response
The cut-off values referred to in the text were taken from the paper published by Pulsford et al in PLoS One (2010) and were established to identify sedentary behaviour and light, moderate and vigorous physical activity using linear discriminant analysis and evaluated using receiver operating characteristic (ROC) curve analysis.
We have changed the text to clarify this as:
“These cut-off values were established to identify sedentary behaviour and light, moderate and vigorous physical activity using linear discriminant analysis and evaluated using receiver operating characteristic curve analysis, and were taken from a calibration study in seven year olds, carried out specifically for this larger study [25].”
Page 7, line 34: The authors state that time spent in MVPA was used to determine adherence to the CMO physical activity guidelines. These require at least 60 minutes of MVPA on each day of the week. Given that relatively few children provided 7 days of data, what decisions were made to operationalise these guidelines? Was it 60 minutes of MVPA on each measurement day? A mean of 60 minutes over several days? Please explain in detail your decisions and justification, since the conclusions you draw may be highly dependent on these.
Response
For each child the dichotomous outcome (does or does not meet CMO physical activity guidelines) was based on the standardised average minutes/day spent in MVPA, where the objective of standardisation was to standardise wearing time across days. This approach is similar to that used in the Gateshead, SPEEDY and ALSPAC studies, presented in table 2. Our rationale was similar to that cited in the ALSPAC study (Riddoch et al 2007), namely that this is a more valid approach than scrutinising each individual day as this would disadvantage children who achieved well in excess of 60 min on one day but who may have narrowly failed to achieve 60 min on another day.
This has been added to the statistical methods section as follows:
“For each child time spent in MVPA was used to determine adherence to the CMO physical activity guideline (≥60 minutes daily) and was based on the standardised average minutes/day spent in MVPA, where the objective of standardisation was to standardise wearing time across days.”
Page 7, line 44: Since BMI and season are known to influence children’s physical activity, why were these not included as explanatory variables.
Response
We thank the reviewer for highlighting this important point. Our study confirms, as reported by ourselves (Rich et al 2012) and others, that season of year is an important determinant of PA and SB behaviour with higher levels of PA observed in Spring and Summer with respect to Winter and Autumn ($p<0.05$). The following table shows the sample sizes, medians, IQRs and prevalences with their standard errors by season.

Season n
Winter 872 545
461, 647 6.7
6.2, 7.3 52.8
42.1, 68.1 9491
7982, 10728 38.8
(0.029)
Spring 656 651
563, 759 6.3
5.8, 6.8 67.6
54.4, 84.1 11178
9745, 12718 65.6
(0.023)
Summer 2790 618
522, 727 6.4
5.8, 6.9 63.3
49.2, 79.7 10533
8909, 12408 55.8
(0.012)
Autumn 2179 558
483, 650 6.7
6.2, 7.2 56.7
44.5, 70.8 9709
8471, 11244 43.5
(0.013)

Due to the order in which the fieldwork was carried out, distribution of season of measurement was not equally distributed across all four UK countries. Children living in Scotland and Northern Ireland were mostly measured during the winter and autumn seasons (84% and 53% of the measurements, respectively), while children living in England and Wales were mostly measured during spring and summer seasons (60% and 54% of the measurements, respectively). As a result season of measurement is confounded with country of residence in the Millennium Cohort Study. We have now adjusted by season of measurement the medians and IQRs reported in Table 1. The main change to the data occurring as a consequence of this adjustment is that estimates of PA and SB in Scotland which were previously reported as significant with respect to England are now no longer significant. Table 1 and the text have been changed accordingly.
The following text has been inserted on page 8
Yearly seasons of measurement were defined astronomically, i.e. spring (21st March –20th June), summer (21st June – 20th September), autumn (21st September – 20th December), winter (21st December – 20th March).
Page 8, line 11: Percentages of what were derived?
Response
The text has been amended (page 9) and now reads:
"Prevalences of the children meeting the CMO current physical activity guideline were derived, as were medians and interquartile ranges for derived activity variables (given their non-normal distributions)."
Page 8, line 13: I am surprised that accelerometer cpm were not Normally distributed. I would expect MVPA to be skewed, but my experience is that cpm are usually Normal.
Response
The sentence about skewness has been amended to clarify the use of median regression. Skewness is only one violation of normality. Heavy tails and heteroscedasticity also represent other violations. All PA outcomes, including cpm, violated normality for one reason or another. The distribution of

MVPA and cpm variables were not normal according the Shapiro-Francia W test for normality ($p<0.05$). Median regression is a robust approach since inference does not depend on distributional assumptions. It is also robust to outliers.
The text has been amended to read:
"These models do not make assumptions about the shape of the distribution. Therefore they are robust to departures from normality, as for example skewness, heavy-tailedness and heteroscedasticity, which affected all PA outcomes in this study."
Page 9, line 16: there are relatively few children in some of the ethnic groups. How confident are you that you can make robust claims about the association between ethnicity and physical activity.
Response
We considered all comparisons between ethnic categories using pairwise multiple comparisons adjusted using the Benjamini-Hochberg procedure as stated in the footnote to Table 1. We feel confident that the associations observed between ethnicity and physical activity are robust.
Page 15, line 58: The derived threshold for MVPA is similar to that of Evenson et al, recently shown to have to have the best classification accuracy for MVPA by Trost et al (MSSE 2011). This may be worth citing to support use of your own threshold.
We thank the reviewer for this suggestion. Evenson suggests a threshold for MVPA of ≥2296cpm which is indeed similar to that derived in our study. This paper has now been cited in support of the threshold we derived:
"They are similar to those reported by Evenson et al [26]."
Page 16, line 9: the underestimation in cycling is not a consequence of the accelerometers used, but rather the positioning of the accelerometers ie worn on a belt around the waist. Given that cycling is a rather infrequent activity, the (likely) small underestimation is probably of little consequence – this could be discussed. Possibly of more importance is the possible removal or under-reporting during other activities such as gym or contact sports.
Response
We thank the reviewer for this correction and have added on page 16 the following phrase (shown in bold):
"Acknowledged limitations include potential underestimation of physical activity, as a result of the accelerometers used, the position in which they are worn, or their removal during some activities such as aquatic activities or contact sports."
Page 18, table: the Wheeler reference is the percentage of time spent in MVPA, not the proportion meeting guidelines.
We thank the reviewer for this correction and have removed reference to this paper from the Table.
Page 18, table footnote: do you mean a mean of 60 or more minutes of MVPA per day?
Response
We thank the reviewer for this observation. The exact measure used in defining adherence to the 60 minute guideline varies across the studies tabulated: MCS, Gateshead, SPEEDY and ALSPAC used a mean of 60 minutes or more, the CHASE study just the first day of measurement, and the Health Survey calculating on a subsample with 7 days of observation, and defined as at least of 60 minutes of MVPA per day on all seven days of observation. We have added the following comment to the Table 2 footnote (p 21):
"Table footnote: *Accumulating ≥60 min/day MVPA The exact measure used in defining adherence to the 60 minute guideline varies across the studies tabulated: MCS, Gateshead, SPEEDY and ALSPAC used a mean of 60 minutes or more, the CHASE study just the first day of measurement, and the Health Survey included a subsample with seven days of observation each with at least 60 minutes of MVPA per day."
Page 19, line 38: discussion around the possible causes of the ethnic and socioeconomic differences is quite minimal. Why, for example, should the less advantaged children be slightly more active? Are country/regional differences actually due to the different environments (urban vs rural). This is touched upon on page 21, but maybe could have been addressed here?
We are unsure of the reasons for the ethnic differences observed in this study but now suggest that

future research is required to explore different cultural reasons, for example, for these.
Page 20, line 3: estimates of sedentary time will depend upon the measurement period and decisions made about removal of zero values. Discussion here could be extended to compare the parameters you used with other studies – this may explain discrepancies.
We agree with the reviewer however previous authors have not always clearly stated the approach used. We have added the following text to page 23 as a comment to acknowledge this issue: "Differences in estimates of sedentary time and numbers of steps between studies may reflect measurement period while decisions made about removal of zero values may also influence sedentary time. These parameters are not consistently reported in published reports."
Page 20, line 17: similarly, steps recorded will depend on the measurement period.
See response to immediately preceding comment.

**VERSION 2 – REVIEW**

| | |
|---|---|
| **REVIEWER** | Lars Bo Andersen, prof at University of Southern Denmark, Denmark<br>I have no conflict of interest |
| **REVIEW RETURNED** | 13-Jun-2013 |

| | |
|---|---|
| **GENERAL COMMENTS** | I believe there are serious problems translating accelerometer counts into percentage of participants fulfilling PA guidelines and we should probably be more critical and not try to recommend on public health based on this uncertain evidence. Who will believe us when the same population can be analyzed so only 0.4% of girls fulfil guideline in one analysis and almost all in another. When this is said, I believe you have handled data appropriately and done the best possible. I just don't think this type of data is suited to make calculations of how many that are sufficiently active. |
